# Supplementary material for: Trends and country-level variation in age at first sex in sub-Saharan Africa among birth cohorts entering adulthood between 1985 and 2020
Source: BMC Public Health. 2022 Jun 4;22:1120. doi: 10.1186/s12889-022-13451-y (PMC9167540; doi:10.1186/s12889-022-13451-y)
Supplement: Supplementary file 1 — Additional file 1. [file 12889_2022_13451_MOESM1_ESM.pdf]

Supplemental Materials:

Trends and country-level variation in age at first sex in sub-Saharan Africa among birth cohorts entering adulthood between 1985 and 2020

Van Kính Nguyen PhD[1], Jeffrey W Eaton PhD[1]

[1] MRC Centre for Global Infectious Disease Analysis, School of Public Health, Imperial College London, London, United Kingdom

Corresponding author:

Van Kính Nguyen
MRC Centre for Global Infectious Disease Analysis, School of Public Health
Imperial College London
St. Mary's Hospital Campus
Norfolk Place
London
W2 1PG
k.nguyen@imperial.ac.uk

Conflicts of Interest: There are no conflict of interests
Keywords: sexual debut, age at first sex, sub-Saharan Africa

Table of contents

Table of contents 1

Supplemental Text 1. Model of age at first sex distribution. 2

Supplemental Figure 1. Estimated AFS's shape and skewness parameters 7

Supplemental Figure 2. Percentage of ever had sex under age 15 7

Supplemental table 1. Survey data characteristics. 8

Supplemental table 2. Proportion ever had sex by country, sex, and year of birth................18

Supplemental table 3. Parameter estimates by country and sex..22

Supplemental table 4. ISO A3 and country names...24

Supplemental Text S1. Model of age at first sex distribution.

Let $T_i, i = 1, \dots, n$ the AFS of individual i, and $d_i = 1$ the indicator of a reported AFS; those who answered never had a sexual intercourse were assumed right censored with $d_i = 0$, that is the individual will eventually have sex at one point in the future; discarding these data will result in lost of valuable information on the time remaining virgin. In these cases, T_i was the age of the individual at the date of interview. We interpreted the reported AFS as the completed year after the last birthday. Thus, reported AFS was not treated as exact records but only represent an interval surrounding the true AFS, i.e., $P(T_i \leq T_i \leq T_i + 1)$. This can be described by an interval censored survival model with the likelihood reads as

$$l(\boldsymbol{\theta}|\mathbf{T}, \mathbf{d}) \propto \prod_{i:d_i=1} w_i^* \left[S(T_i) - S(T_i + 1)\right] \prod_{i:d_i=0} w_i^* \, S(T_i),$$

in which $f(.)$ the probability density function (pdf), and $S(.)$ the survival function of a selected distribution for AFS. The likelihood is weighted by the inverse selection probability from the survey sampling design $\boldsymbol{w}^*$; here, the weight for observation i in survey k was scaled as $w_{ik}^* = w_{ik} \, \mathrm{n_k} / \sum_i w_{ik}$ such that the weights sum to the total sample size n_k of each survey.

To allow nonmonotonic hazard function for the sexual debut rate by age and allow for possible skewness in the data, the log of AFS, $\log(x)$, was assumed to follow a skew-logistic distribution, which we have shown elsewhere to be a more appropriate representation of the AFS distribution than other common survival distributions (Nguyen and Eaton 2021). The log-skew-logistic distribution was defined by the probability density function

$$f(x) = \gamma p \exp(\mu - x) \left(1 + \exp\left(p(\mu - x)\right)\right)^{-\gamma - 1}$$

and cumulative distribution

$$F(x) = \left[1 + \exp\left(p(\mu - x)\right)\right]^{-\gamma}.$$

The parameters $\mu, p > 0, \gamma > 0$ determine the scale, shape, and skewness of the distribution, respectively; the distribution reduces to a logistic distribution when $\gamma = 1$. Illustration of the density distribution can be found in Figure 1.

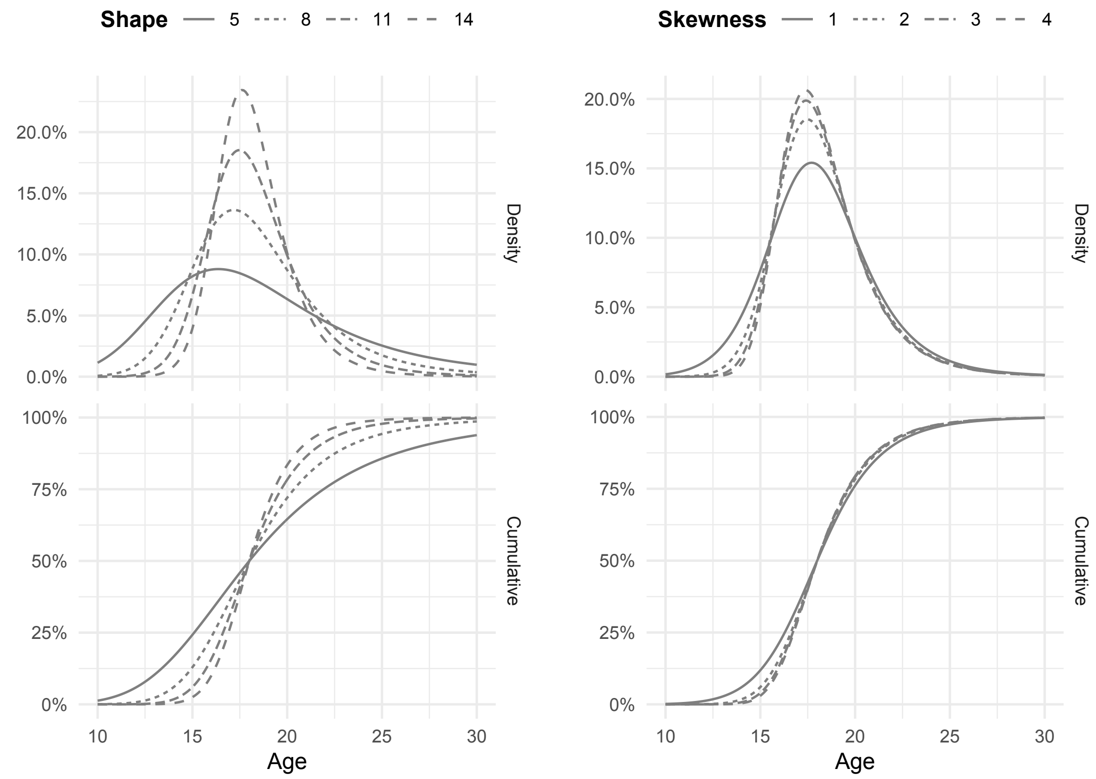

Figure 1. Illustration of the density (top) and cumulative distribution function (bottom) for the skew log-logistic distribution with different shape and skewness parameters. All parameters correspond to a median age at first sex of 18 years. A skewness value of one equal to no skewness and is the same as the logistic distribution. The density and cumulative panel on the right illustrate the relative effect of changing the respective parameter on the distribution while keeping other parameters fixed (skewness=2 and shape=11 for the shape and skewness variation, respectively; the scale parameter was solved to have the same median at eighteen for comparison).

Parameterize the model's linear predictor as $\mu = -\log(\lambda) = -\boldsymbol{X\beta}$ and perform a change of variable to T, the reported AFS, we have

$$S(T) = 1 - (1 + (\lambda T)^{-p})^{-\gamma}$$

and

$$f(T) = T^{-1}\gamma p(\lambda T)^{-p}(1 + (\lambda T)^{-p})^{-\gamma-1};$$

these two were used to evaluate the model likelihood.

The 'hazard' of having sexual debut at age T is

$$h(T) = -\partial \ln S(T)/\partial T = -T^{-1}\gamma p\big((1 + (\lambda T)^{p})((1 + (\lambda T)^{-p})^{-\gamma}) - 1\big)^{-1}$$

and the median is

$$\lambda^{-1}\big(2^{\gamma^{-1}} - 1\big)^{-p^{-1}}.$$

The distribution IQR can be computed using the quantile function defined as

$$AFS = \frac{1}{\lambda}\left(Q^{-\gamma^{-1}} - 1\right)^{-p^{-1}}$$

where Q the quantile of interest.

The model was fitted separately by sex but simultaneously for all countries. For each country $c, c = 1, \dots, C$, the shape and skewness parameters p_c, γ_c were estimated independently. The linear predictor was modelled on the scale parameter $\mu = -\log(\lambda) = -\boldsymbol{X\beta}$. This was log-link to the model's linear predictor and was formulated as

$$\eta_{cta} = \beta_0 + \theta_c + \alpha_t + \delta_{ct} + \phi_a + \omega_{ca}$$

where β_0 the intercept, $\theta_c, \alpha_t, \delta_{ct}, \phi_a, \omega_{ca}$ are the spatial, birth cohort, country-birth cohort interaction, age at report effect, country-age interaction each has its corresponding precision parameter τ. The birth cohort and age at report main effects were structured as a second-order autoregressive (AR2) models and a first-order random walk (RW1) for which we prefer a smoother variation of trend compared to a more flexible age at report bias. The Intrinsic Conditional Auto-Regressive (ICAR) (Besag and Kooperberg 1995) was used to model the spatial effect on the distribution's shape parameter; its density defined as

$$\pi(\theta_c|\tau_c) \propto \exp\left(0.5\tau_c \sum_{i\sim i\prime}(\theta_i - \theta_{i'})^2\right),$$

where $i \sim i'$denotes all the i'neighbours of the location i. We assumed interactions that allow the temporal trend and age effect to vary independently between countries. These interactions were required to address our primary research question of whether trends in AFS were different across countries, allowing for potentially differential reporting biases across countries and between sexes. The space-time prior density was defined using a separable density between and IID and AR2 for country-birth cohort interaction and IID and RW1 for country-age at report bias interaction, each having their own correlation parameter vector (for the AR2 dimension) and precision parameter (for the RW1 dimension).

Sum to zero constraints were imposed on each of the main random effect parameters, $\sum\theta_c = \sum\alpha_t = \sum\phi_a = 0$. On the interaction terms, additional sum to zero constraints were put on the direction of birth cohort and age for each country in order to estimate the main

country effect, i.e., $\sum_t \delta_{ct} = 0, \sum_a \omega_{ca} = 0$, as well as across countries within each birth cohort and age, i.e., $\sum_c \delta_{ct} = \sum_c \omega_{ca} = 0$. Since several countries (four male datasets and three female datasets) had only one survey with AFS, to estimate age at report effect an additional linear constraint were imposed on the country and age at report interaction. Consequently, while the birth cohort effect was allowed to have different trends among countries, the age at report effect was only allowed to be deviated from the regional trend if they there are more than one survey dataset to inform its pattern.

Priors for all the precision parameters was specified using the penalized-complexity prior (Simpson et al. 2017) with small variances across the hyperparameters, i.e., $\tau_c \sim PC(1, 0.01)$, $\tau_a \sim PC(1,\ 0.01)$, $\tau_p \sim PC(1,\ 0.01)$, $\tau_\gamma \sim PC(1,\ 0.01)$. Based on initial exploration of the distribution parameters, we found that the shape and skewness parameters are highly correlated; thus we used the identified parameter estimates to reparametrize $\gamma_c^* = \log \gamma_c + 1.1 \log p_c$ and independent informative priors were put on $\log p_c \sim N(2.2{,}0.25)$ and $\gamma_c^* \sim \mathrm{N}(2.8{,}0.35)$ such that the priors cover the identified range of the shape and skewness parameters. The main birth cohort AR2 and interaction between birth cohort with country each has two correlation parameters $\boldsymbol{\phi}$. To constraint the correlation parameters within their valid region, these parameters were reparametrized using the partial autocorrelation function

$$\psi_i = 2 \times \frac{\exp(\theta_i)}{1 + \exp(\theta_i)} - 1$$

where $\phi_2 = \psi_2$, $\phi_1 = \psi_1(1 - \phi_2)$. A multivariate standard normal distribution $MVN(\mathbf{0}, \mathbf{1})$ was used as prior for the parameters $\boldsymbol{\theta}$. Model parameters were estimated with empirical Bayes using Template Model Builder (Kristensen et al. 2016). The code is available at https://github.com/kklot/dbtmb.

References

Besag, Julian, and Charles Kooperberg. 1995. ‘On Conditional and Intrinsic Autoregression’. *Biometrika* 82 (4): 733–46. https://doi.org/10.2307/2337341.

Kristensen, Kasper, Anders Nielsen, Casper W. Berg, Hans Skaug, and Bradley M. Bell. 2016. ‘TMB: Automatic Differentiation and Laplace Approximation’. *Journal of Statistical Software* 70. https://doi.org/10.18637/jss.v070.i05.

Nguyen, Van Kinh, and Jeffrey W Eaton. 2021. ‘A Model for Reconstructing Trends and Distribution in Age at First Sex from Multiple Household Surveys with Reporting Biases’. *MedRxiv*, January, 2021.12.14.21267770. https://doi.org/10.1101/2021.12.14.21267770.

Simpson, Daniel, Håvard Rue, Andrea Riebler, Thiago G. Martins, and Sigrunn H. Sørbye. 2017. ‘Penalising Model Component Complexity: A Principled, Practical Approach to Constructing Priors’. *Statistical Science* 32 (1): 1–28. https://doi.org/10.1214/16-STS576.

Supplemental Figure S1. Estimated AFS's shape and skewness parameters

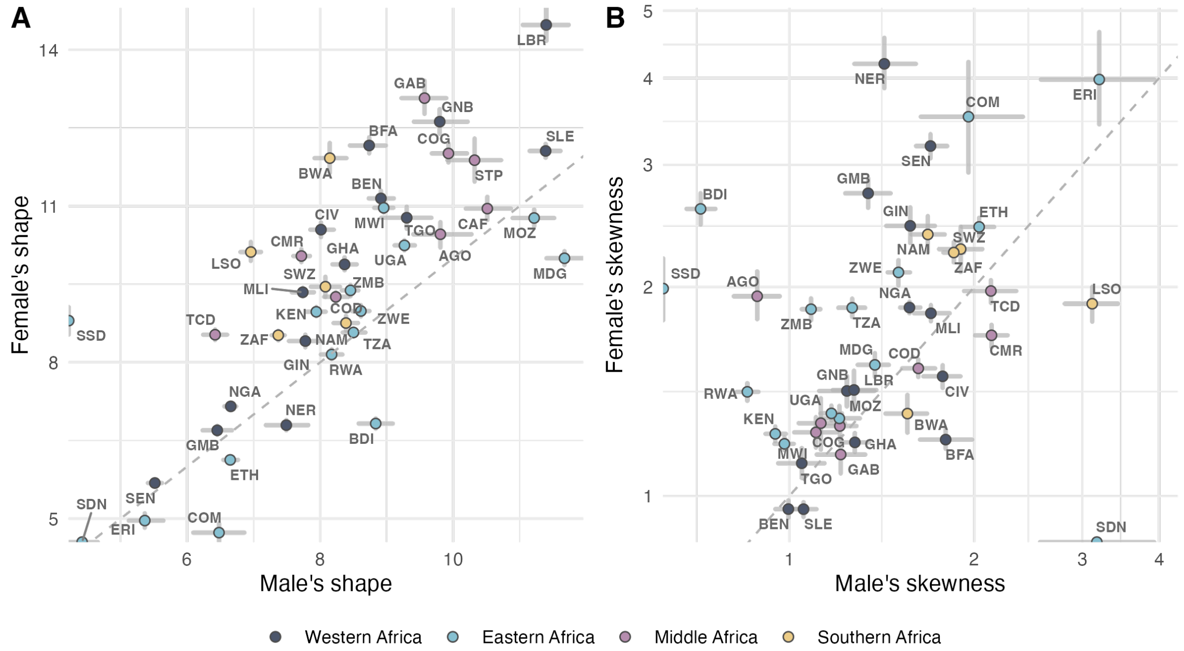

Estimates of the skew logistic shape and skewness parameter by country and sex. The crossed lines in panel A and B show the associated uncertainty interval (UI) in the direction of each axis. Sudan and South-Sudan has only one of the sexes data and are shown with the UI in only one direction and was put arbitrarily on the axes.

Supplemental Figure S2. Percentage of ever had sex under age 15

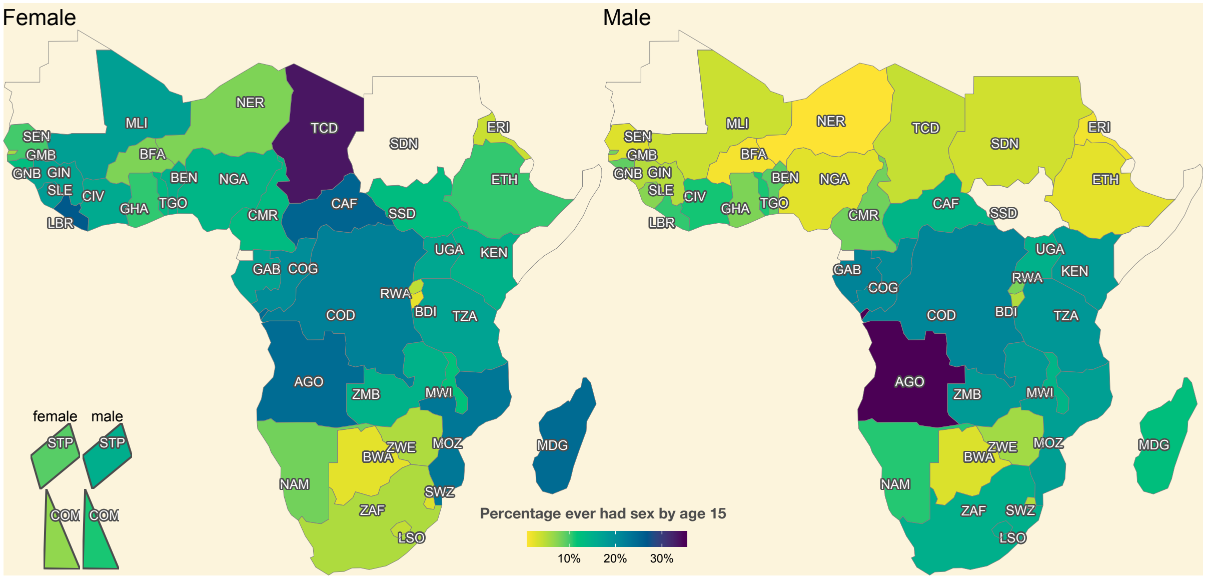

Spatial variation of proportion ever had sex under fifteen of the birth cohort turning age 15 in 2015 in Sub-Saharan Africa. The two countries Comoros and São Tomé & Príncipe are represented in the bottom left. Countries without data are colored the same as the background.

Supplemental Table S1. Survey data characteristics.

The table reports the total number of respondents in each survey (Sample Size) and the percentage (%) of cases removed due to each exclusion criteria: missing AFS outcome, missing age or date of birth (DOB), AFS > Age, AFS < 7 years.

ISO_A3	: ISO 3166-1 alpha-3 code for countries
AFS	: Age at first sex
DHS	: Demographic and Health Surveys
MICS	: Multiple Indicators Cluster Surveys
PMA	: Performance Monitoring and Accountability
AIS	: AIDS Indicators Survey
PHIA	: Population-based HIV Impact Assessment
HSRC	: Human Sciences Research Council
SBS	: Sexual Behavior Survey
-	: Zero or less than 0.05%

The column "Sample size" refers to the count of the data at reading with non-missing and valid coded sex (1 or 2). "Missing AFS" refers to all observations those did not have a record of AFS in the dataset due to either non-answered or ineligibility in the survey's context (e.g., excluded from the questionnaire's sexual behaviour module because aged older than 24 in MICS, recorded as "Not applicable" in HSRC), cases flagged as incorrect depending on survey, e.g., in DHS and MICS data, AFS coded as 97 is inconsistent report such that respondent's AFS exceeds current age, more than one year after the conception of her first child, and others (e.g., don't know coded as 98) [1]; this also counted the missing data when the missing data was coded as a number, such as 99 in MICS. "Missing age/DOB" refers to cases when year of birth cannot be calculated due to missing both age and time of birth. "Ever sex only" refers to cases answered to have ever had sex, but did not answer a particular age, such as refusing, "don't know" (coded as 9, 88, 98 in BAIS, KAIS, DHS).

1. ICF. 2018. Demographic and Health Surveys Standard Recode Manual for DHS7. The Demographic and Health Surveys Program. Rockville, Maryland, U.S.A.: ICF

ISO_A3	Sex	Year	Survey	Sample Size	Missing AFS	Missing age/DOB	AFS > age	AFS < 7	'Ever sex' only
AGO	Female	2015-16	DHS	14379	-	-	-	-	5.6
AGO	Male	2015-16	DHS	5684	-	-	-	-	0.7
BDI	Female	2010-11	DHS	9389	-	-	-	-	3.2
BDI	Female	2016-17	DHS	34538	-	-	-	-	0.9
BDI	Male	2010-11	DHS	4280	-	-	-	-	4.1
BDI	Male	2016-17	DHS	15104	-	-	-	-	1.3
BEN	Female	1996	DHS	5491	0.2	-	-	-	2.1
BEN	Female	2001	DHS	6219	0.3	-	-	-	1.9
BEN	Female	2006	DHS	17794	0.1	-	-	-	6.5
BEN	Female	2011-12	DHS	16599	-	-	-	-	11.4
BEN	Female	2014	MICS	16348	3.8	3.3	-	0.1	-
BEN	Female	2017-18	DHS	15928	-	-	-	-	0.2
BEN	Male	1996	DHS	1535	0.7	-	-	-	0.3
BEN	Male	2001	DHS	2709	0.2	-	-	-	-
BEN	Male	2006	DHS	5321	0.1	-	-	-	-
BEN	Male	2011-12	DHS	5180	-	-	-	-	2.8
BEN	Male	2014	MICS	4577	5.1	4.5	-	-	-
BEN	Male	2017-18	DHS	7595	-	-	-	-	-
BFA	Female	1992-93	DHS	6354	3.5	-	-	-	1.4

ISO_A3	Sex	Year	Survey	Sample Size	Missing AFS	Missing age/DOB	AFS > age	AFS < 7	'Ever sex' only
BFA	Female	1998-99	DHS	6445	0.1	-	-	-	6.5
BFA	Female	2003	DHS	12477	0.3	-	-	-	4.8
BFA	Female	2010	DHS	17087	0.1	-	-	-	2.4
BFA	Female	2014-15	PMA	2219	10	-	-	1.7	11.4
BFA	Female	2015	PMA	9657	78.3	77.7	-	-	1.9
BFA	Female	2016	PMA	3493	5	3.7	-	-	4
BFA	Female	2016-17	PMA	3413	5	4.5	-	0.1	1
BFA	Female	2017-18	PMA	3659	2.9	2.5	-	0.1	1.1
BFA	Female	2018-19	PMA	3510	3.1	2.6	-	-	0.8
BFA	Male	1998-99	DHS	2641	0.3	-	-	-	5.3
BFA	Male	2003	DHS	3605	0.2	-	-	-	-
BFA	Male	2010	DHS	7307	0.1	-	-	-	1.9
BWA	Female	2001	AIS	2362	0.1	-	-	-	3.6
BWA	Female	2004	AIS	8691	-	-	-	-	6.6
BWA	Female	2008	AIS	7783	0.8	-	-	-	5.7
BWA	Male	2001	AIS	2030	0.3	0.1	-	-	5.8
BWA	Male	2004	AIS	7143	-	-	-	-	7.4
BWA	Male	2008	AIS	6424	0.8	-	-	-	7
CAF	Female	1994-95	DHS	5884	0.2	-	-	-	0.6
CAF	Female	2010	MICS	12507	8.3	-	-	0.1	-
CAF	Male	1994-95	DHS	1729	0.2	-	-	-	0.5
CAF	Male	2010	MICS	6128	13.6	13.3	0.1	-	-
CIV	Female	1994	DHS	8099	0.1	-	-	-	0.9
CIV	Female	1998-99	DHS	3040	0.2	-	-	-	1.3
CIV	Female	2005	DHS	9686	0.2	-	-	-	1.1
CIV	Female	2011-12	DHS	10060	0.2	-	-	-	5.7
CIV	Female	2016	MICS	12463	6	5.5	-	-	-
CIV	Female	2017	PMA	2877	3.8	3.1	-	-	3.5
CIV	Female	2017-18	PHIA	12313	28.6	-	-	-	-
CIV	Female	2018	PMA	2864	2.6	2.3	-	0.1	0.2
CIV	Male	1998-99	DHS	886	0.1	-	-	-	-
CIV	Male	2011-12	DHS	5135	0.3	-	-	-	1.5
CIV	Male	2016	MICS	5919	9.3	8.7	-	-	-
CIV	Male	2017-18	PHIA	11988	24.8	-	-	-	-
CMR	Female	1991	DHS	3871	0.3	-	-	-	6.2
CMR	Female	1998	DHS	5501	0.1	-	-	-	2.3
CMR	Female	2004	DHS	10656	0.2	-	-	-	3.5
CMR	Female	2011	DHS	15426	0.1	-	-	-	5.8
CMR	Female	2014	MICS	10447	5.8	-	-	-	-
CMR	Female	2017-18	PHIA	17394	13.8	-	-	-	-
CMR	Female	2018-19	DHS	14677	-	-	-	-	1.7
CMR	Male	1998	DHS	2562	0.1	-	-	-	-
CMR	Male	2004	DHS	5280	0.3	-	-	-	0.1
CMR	Male	2011	DHS	7191	0.3	-	-	-	-
CMR	Male	2014	MICS	5345	9.4	9.2	-	-	-
CMR	Male	2017-18	PHIA	14981	15.7	-	-	-	-
CMR	Male	2018-19	DHS	6978	-	-	-	-	1.1
COD	Female	2007	DHS	9995	0.1	-	-	-	4.8
COD	Female	2010	MICS	13235	3	-	-	0.1	-

ISO_A3	Sex	Year	Survey	Sample Size	Missing AFS	Missing age/DOB	AFS > age	AFS < 7	'Ever sex' only
COD	Female	2012-14	PMA	2163	1.4	100	-	-	3.6
COD	Female	2013-14	DHS	18827	0.1	-	-	-	3.2
COD	Female	2014	PMA	11228	74.8	73.2	-	-	1
COD	Female	2015	PMA	2835	4.8	0.1	-	-	2.9
COD	Female	2015-16	PMA	4514	4.4	3.5	-	0.1	4
COD	Female	2016	PMA	4507	5	4.3	-	0.1	5
COD	Female	2017	PMA	4465	4.3	3.8	-	-	3.4
COD	Female	2017-18	MICS	21828	0.4	-	-	-	-
COD	Female	2018	PMA	4503	3.8	3.3	-	0.1	2.1
COD	Female	2019	PMA	1942	1	0.6	-	-	2.1
COD	Male	2007	DHS	4757	0.2	-	-	-	1.2
COD	Male	2013-14	DHS	8656	0.1	-	-	-	0.9
COD	Male	2017-18	MICS	6161	0.8	0.8	-	-	-
COG	Female	2005	DHS	7051	-	-	-	-	0.5
COG	Female	2009	DHS	12413	0.5	-	-	-	1.5
COG	Female	2011-12	DHS	10819	0.1	-	-	-	2.1
COG	Female	2014-15	MICS	11841	5	4.6	-	-	-
COG	Male	2005	DHS	3146	-	-	-	-	-
COG	Male	2011-12	DHS	5145	-	-	-	-	0.3
COG	Male	2014-15	MICS	5412	6.4	-	-	-	-
COM	Female	1996	DHS	3050	0.4	-	-	-	2.6
COM	Female	2012	DHS	5329	0.2	-	-	-	6.5
COM	Male	1996	DHS	795	2.1	-	-	-	-
COM	Male	2012	DHS	2167	0.7	-	-	-	1.5
ERI	Female	2010	PHS	31701	68.8	4.7	-	-	-
ERI	Male	2010	PHS	6436	25.1	22	-	-	-
ETH	Female	2000	DHS	15367	0.1	-	-	0.1	2.6
ETH	Female	2005	DHS	14070	0.1	-	-	-	3.8
ETH	Female	2010-11	DHS	16515	0.1	-	-	-	4.2
ETH	Female	2014	PMA	13561	2.5	1.6	0.1	0.1	3.7
ETH	Female	2015	PMA	7705	1.1	0.9	0.1	-	1.8
ETH	Female	2016	DHS	15683	-	-	-	-	1.6
ETH	Female	2016	PMA	7641	1.4	1.3	0.1	0.1	2.1
ETH	Female	2017	PMA	7577	1.6	1.4	-	-	2.1
ETH	Female	2018	PMA	7690	2.2	1.9	-	0.1	1.5
ETH	Female	2019	PMA	9106	2.2	2	-	0.1	1.1
ETH	Male	2000	DHS	2607	-	-	-	-	1.8
ETH	Male	2005	DHS	6033	0.1	-	-	-	1.1
ETH	Male	2010-11	DHS	14110	0.1	-	-	-	3
ETH	Male	2016	DHS	12688	-	-	-	-	1.4
GAB	Female	2000-01	DHS	6183	0.1	-	-	-	3.5
GAB	Female	2012	DHS	8422	0.1	-	-	-	5.6
GAB	Male	2000-01	DHS	2004	0.2	-	-	0.1	0.1
GAB	Male	2012	DHS	5654	0.2	-	-	-	0.6
GHA	Female	1993-94	DHS	4562	0.2	-	-	-	0.2
GHA	Female	1998-99	DHS	4843	0.1	-	-	-	4.7
GHA	Female	2003	DHS	5691	0.1	-	-	-	5.8
GHA	Female	2006	MICS	6240	5.9	5.6	-	0.1	-
GHA	Female	2008	DHS	4916	-	-	-	-	4.7

ISO_A3	Sex	Year	Survey	Sample Size	Missing AFS	Missing age/DOB	AFS > age	AFS < 7	'Ever sex' only
GHA	Female	2011	MICS	10963	3.1	-	-	-	-
GHA	Female	2012-13	PMA	4155	11.4	100	-	0.4	3.5
GHA	Female	2014	DHS	9396	-	-	-	-	3.4
GHA	Female	2014	PMA	9035	6.1	3.8	-	0.1	4.4
GHA	Female	2015	PMA	5390	3.3	2.4	-	0.1	2.8
GHA	Female	2016	PMA	3850	4	2.7	-	-	3.7
GHA	Female	2017-18	MICS	14609	1.9	-	-	-	-
GHA	Male	1993-94	DHS	1302	0.3	-	-	-	-
GHA	Male	1998-99	DHS	1546	0.1	-	-	-	0.1
GHA	Male	2003	DHS	5015	0.1	-	-	-	0.4
GHA	Male	2008	DHS	4568	0.1	-	-	-	0.5
GHA	Male	2011	MICS	3511	5.4	5.4	-	-	-
GHA	Male	2014	DHS	4388	-	-	-	-	1.3
GHA	Male	2017-18	MICS	5476	2.8	2.8	-	-	-
GIN	Female	1999	DHS	6753	0.2	-	-	-	7.2
GIN	Female	2005	DHS	7954	0.2	-	-	-	6.5
GIN	Female	2012	DHS	9142	0.1	-	-	-	2.7
GIN	Female	2016	MICS	10245	6.6	-	-	-	-
GIN	Female	2018	DHS	10874	-	-	-	-	2.4
GIN	Male	1999	DHS	1980	0.4	-	-	-	-
GIN	Male	2005	DHS	3174	0.2	-	-	-	0.1
GIN	Male	2012	DHS	3782	0.4	-	-	-	0.1
GIN	Male	2018	DHS	4117	-	-	-	-	2
GMB	Female	2010	MICS	15138	3	-	-	0.1	-
GMB	Female	2013	DHS	10233	0.1	-	-	-	4.5
GMB	Female	2017-18	MICS	14297	4.7	-	-	-	-
GMB	Female	2019-20	DHS	11865	0.7	-	-	-	-
GMB	Male	2013	DHS	3821	0.3	-	-	-	2.7
GMB	Male	2017-18	MICS	5213	18.6	13.3	-	-	-
GMB	Male	2019-20	DHS	4636	0.9	-	-	-	-
GNB	Female	2014	MICS	10744	4.8	-	-	-	-
GNB	Male	2014	MICS	4620	8.4	8.4	-	-	-
IDN	Female	2015	PMA	11585	9.5	0.1	-	-	1.2
IDN	Female	2016-17	PMA	11379	7	6.1	-	-	0.6
IND	Female	2016	PMA	5658	6.7	3.6	-	0.2	5
IND	Female	2017	PMA	12414	2.4	2	-	-	0.4
IND	Female	2018	PMA	6064	2.5	2.5	-	0.1	0.1
KEN	Female	1993	DHS	7540	0.4	-	-	-	5.6
KEN	Female	1998	DHS	7881	0.3	-	-	-	3.8
KEN	Female	2003	DHS	8195	0.2	-	-	-	4.3
KEN	Female	2007	AIS	10957	12.2	6.5	0.1	-	-
KEN	Female	2008-09	DHS	8444	0.1	-	-	-	6.4
KEN	Female	2012-13	AIS	7898	-	-	-	-	7.8
KEN	Female	2014	DHS	31079	0.1	-	-	0.1	4.1
KEN	Female	2014	PMA	8456	5.7	-	-	0.1	4.8
KEN	Female	2015	PMA	9529	3	1.3	-	-	3.6
KEN	Female	2016	PMA	6048	2.3	1.1	-	0.1	2.8
KEN	Female	2017	PMA	5977	1.8	1.1	-	0.1	1.2
KEN	Female	2018-19	PMA	5776	1.3	0.9	-	-	0.8

ISO_A3	Sex	Year	Survey	Sample Size	Missing AFS	Missing age/DOB	AFS > age	AFS < 7	'Ever sex' only
KEN	Female	2019	PMA	9699	2	1.4	-	0.1	0.5
KEN	Male	1993	DHS	2336	0.3	-	-	-	0.1
KEN	Male	1998	DHS	3407	1	-	-	-	4.6
KEN	Male	2003	DHS	3578	0.3	-	-	-	0.2
KEN	Male	2007	AIS	8883	15.7	13.3	0.1	-	-
KEN	Male	2008-09	DHS	3465	-	-	-	-	0.6
KEN	Male	2012-13	AIS	5713	-	-	-	-	6.1
KEN	Male	2014	DHS	12819	0.1	-	-	0.4	0.7
LBR	Female	2006-07	DHS	7092	0.5	-	-	-	5.9
LBR	Female	2013	DHS	9239	-	-	-	-	4.3
LBR	Male	2006-07	DHS	6009	0.2	-	-	-	0.8
LBR	Male	2013	DHS	4118	-	-	-	-	0.9
LSO	Female	2004-05	DHS	7095	0.2	-	-	-	5.7
LSO	Female	2009-10	DHS	7624	-	-	-	-	1.6
LSO	Female	2014	DHS	6621	-	-	-	-	1
LSO	Female	2016-17	PHIA	10746	30.2	-	-	-	-
LSO	Male	2004-05	DHS	2797	0.3	-	-	-	1.4
LSO	Male	2009-10	DHS	3317	-	-	-	-	4.9
LSO	Male	2014	DHS	2931	-	-	-	-	2
LSO	Male	2016-17	PHIA	8770	39	-	-	-	-
MDG	Female	1992	DHS	6260	0.1	-	-	-	0.3
MDG	Female	1997	DHS	7060	0.1	-	-	-	1.7
MDG	Female	2003-04	DHS	7949	0.1	-	-	-	2.8
MDG	Female	2008-09	DHS	17375	-	-	-	-	2.1
MDG	Female	2018	MICS	18812	9	-	-	-	-
MDG	Male	2003-04	DHS	2432	0.2	-	-	-	-
MDG	Male	2008-09	DHS	8586	0.1	-	-	-	0.3
MDG	Male	2018	MICS	8980	15.2	0.1	-	-	-
MLI	Female	1995-96	DHS	9704	0.1	-	-	-	1.5
MLI	Female	2001	DHS	12849	0.3	-	-	-	3.7
MLI	Female	2006	DHS	14583	0.1	-	-	-	5.8
MLI	Female	2009-10	MICS	28846	7.5	-	-	-	-
MLI	Female	2012-13	DHS	10424	-	-	-	-	11.5
MLI	Female	2015	MICS	18409	7	-	-	-	-
MLI	Female	2018	DHS	10519	-	-	-	-	2
MLI	Male	1995-96	DHS	2474	-	-	-	-	-
MLI	Male	2001	DHS	3405	0.7	-	-	-	-
MLI	Male	2006	DHS	4207	0.3	-	-	-	2.5
MLI	Male	2012-13	DHS	4399	-	-	-	-	4.9
MLI	Male	2015	MICS	8419	12.5	11.8	-	-	-
MLI	Male	2018	DHS	4618	-	-	-	-	1.3
MOZ	Female	1997	DHS	8779	1.3	-	-	-	1.9
MOZ	Female	2003-04	DHS	12418	0.1	-	-	-	2.7
MOZ	Female	2009	DHS	11212	0.3	-	-	-	0.1
MOZ	Female	2011	DHS	13745	-	-	-	-	3.2
MOZ	Female	2015	DHS	7749	0.1	-	-	-	1.6
MOZ	Male	1997	DHS	2335	0.9	-	-	-	2.9
MOZ	Male	2003-04	DHS	2900	0.3	-	-	-	0.9
MOZ	Male	2011	DHS	4035	-	-	-	-	2.2

ISO_A3	Sex	Year	Survey	Sample Size	Missing AFS	Missing age/DOB	AFS > age	AFS < 7	'Ever sex' only
MOZ	Male	2015	DHS	5283	-	-	-	-	1.7
MWI	Female	2000	DHS	13220	0.1	-	-	-	2.8
MWI	Female	2004-05	DHS	11698	0.2	-	-	-	7.3
MWI	Female	2006	MICS	27073	3.2	3	-	-	-
MWI	Female	2010	DHS	23020	0.1	-	-	-	5.1
MWI	Female	2013-14	MICS	25430	4.8	-	-	-	-
MWI	Female	2015-16	DHS	24562	-	-	-	-	2.7
MWI	Female	2015-16	PHIA	14158	21.4	-	-	0.1	-
MWI	Female	2019-20	MICS	24543	-	-	-	-	-
MWI	Male	2000	DHS	3092	0.3	-	-	-	0.1
MWI	Male	2004-05	DHS	3261	0.2	-	-	-	1.9
MWI	Male	2010	DHS	7175	0.1	-	-	-	1.6
MWI	Male	2013-14	MICS	7818	12.6	12.5	-	-	-
MWI	Male	2015-16	DHS	7478	-	-	-	-	0.9
MWI	Male	2015-16	PHIA	12713	35.9	-	-	0.2	-
MWI	Male	2019-20	MICS	6791	0.1	-	-	-	-
NAM	Female	1992	DHS	5421	1.3	-	-	-	6.3
NAM	Female	2000	DHS	6755	1.1	-	-	-	8.8
NAM	Female	2006-07	DHS	9804	0.6	-	-	-	7.3
NAM	Female	2013	DHS	10018	0.7	-	-	-	5.6
NAM	Female	2017	PHIA	14160	33.8	-	-	-	-
NAM	Male	2000	DHS	2954	1.5	-	-	-	-
NAM	Male	2006-07	DHS	3915	0.7	-	-	-	0.7
NAM	Male	2013	DHS	4481	0.8	-	-	-	0.7
NAM	Male	2017	PHIA	12361	39.5	-	-	-	-
NER	Female	1992	DHS	6503	0.6	-	-	-	3.6
NER	Female	2006	DHS	9223	0.3	-	-	-	4.2
NER	Female	2012	DHS	11160	0.1	-	-	-	4.4
NER	Female	2015	PMA	1408	5.7	3.8	-	-	2.9
NER	Female	2016	PMA	3193	6.3	4.6	-	0.2	2.9
NER	Female	2017	PMA	3180	4.8	4.5	-	-	0.9
NER	Female	2018	PMA	1390	6.9	6.3	-	-	0.9
NER	Male	1998	DHS	3542	0.3	-	-	-	0.1
NER	Male	2006	DHS	3549	0.4	-	-	-	0.1
NER	Male	2012	DHS	3928	0.3	-	-	-	2.8
NGA	Female	1990	DHS	8781	0.5	-	-	0.2	9.7
NGA	Female	2003	DHS	7620	0.4	-	-	-	5.1
NGA	Female	2008	DHS	33385	0.4	-	-	-	6.5
NGA	Female	2011	MICS	33699	8.8	-	-	0.3	-
NGA	Female	2013	DHS	38948	0.2	-	-	-	5.2
NGA	Female	2016	PMA	11445	4.8	2.3	-	0.4	4.2
NGA	Female	2016-17	MICS	36176	6.6	5	-	-	-
NGA	Female	2017	PMA	11624	3	1.4	-	0.1	3.5
NGA	Female	2018	DHS	41821	-	-	-	-	2.4
NGA	Female	2018	PMA	11470	3	2.1	-	0.1	2.1
NGA	Female	2019	PMA	2016	1.6	0.4	-	-	0.7
NGA	Male	2003	DHS	2346	0.2	-	-	-	-
NGA	Male	2008	DHS	15486	0.3	-	-	-	2.8
NGA	Male	2013	DHS	17359	0.2	-	-	-	3.5

ISO_A3	Sex	Year	Survey	Sample Size	Missing AFS	Missing age/DOB	AFS > age	AFS < 7	'Ever sex' only
NGA	Male	2016-17	MICS	16514	9.9	8.1	-	0.1	-
NGA	Male	2018	DHS	13311	-	-	-	-	1.6
RWA	Female	1992	DHS	6551	0.5	-	-	-	1.2
RWA	Female	2000	DHS	10421	-	-	-	0.1	1.2
RWA	Female	2005	DHS	11321	-	-	-	0.3	2
RWA	Female	2010-11	DHS	13671	-	-	-	-	1.5
RWA	Female	2014-15	DHS	13497	-	-	-	0.3	1.3
RWA	Female	2018-19	PHIA	18988	11.5	-	-	-	-
RWA	Female	2019-20	DHS	14634	0.2	-	-	-	-
RWA	Male	2000	DHS	2717	0.3	-	-	0.4	1.4
RWA	Male	2005	DHS	4820	0.1	-	-	1.2	1
RWA	Male	2010-11	DHS	6329	-	-	-	0.5	1.9
RWA	Male	2014-15	DHS	6217	-	-	-	0.8	2
RWA	Male	2018-19	PHIA	16277	15.8	-	-	-	-
RWA	Male	2019-20	DHS	6513	0.8	-	-	-	-
SDN	Male	2010	MICS	16448	66.2	66.1	-	-	1.6
SEN	Female	1992-93	DHS	6310	0.5	-	-	-	4.7
SEN	Female	1997	DHS	8593	1.2	-	-	-	4.8
SEN	Female	2005	DHS	14602	0.3	-	-	-	5.8
SEN	Female	2010-11	DHS	15688	-	-	-	-	5.3
SEN	Female	2012-13	DHS	8636	-	-	-	-	2.8
SEN	Female	2014	DHS	8488	-	-	-	-	2.6
SEN	Female	2015	DHS	8851	-	-	-	-	2.2
SEN	Female	2016	DHS	8865	-	-	-	-	2
SEN	Female	2017	DHS	16787	-	-	-	-	2.5
SEN	Female	2018	DHS	9414	-	-	-	0.1	2.2
SEN	Female	2019	DHS	8649	2	-	-	0.1	-
SEN	Female	2019	DHS	8649	-	-	-	0.1	2
SEN	Male	1997	DHS	4306	0.6	-	-	-	11.2
SEN	Male	2005	DHS	3761	0.5	-	-	-	0.2
SEN	Male	2010-11	DHS	4929	-	-	-	-	3
SEN	Male	2014	DHS	3371	-	-	-	-	2.4
SEN	Male	2015	DHS	3734	-	-	-	-	2.2
SEN	Male	2016	DHS	3527	-	-	-	-	1.7
SEN	Male	2017	DHS	6977	-	-	-	-	2.6
SEN	Male	2018	DHS	3762	-	-	-	-	2.5
SEN	Male	2019	DHS	3365	2.4	-	-	-	-
SEN	Male	2019	DHS	3365	-	-	-	-	2.4
SLE	Female	2008	DHS	7374	0.5	-	-	-	9.8
SLE	Female	2010	MICS	14066	5.3	-	-	0.1	-
SLE	Female	2013	DHS	16658	0.2	-	-	-	8.8
SLE	Female	2017	MICS	18006	1.1	0.3	-	0.7	-
SLE	Female	2019	DHS	15574	1.2	-	-	-	-
SLE	Male	2008	DHS	3280	0.5	-	-	-	1.5
SLE	Male	2013	DHS	7262	0.2	-	-	-	1.4
SLE	Male	2017	MICS	7534	2	1.6	0.3	-	-
SLE	Male	2019	DHS	7197	1	-	-	-	-
SSD	Female	2010	MICS	11568	22.1	-	-	-	18
STP	Female	2008-09	DHS	2615	0.1	-	-	-	3.6

ISO_A3	Sex	Year	Survey	Sample Size	Missing AFS	Missing age/DOB	AFS > age	AFS < 7	'Ever sex' only
STP	Female	2014	MICS	3101	5.4	-	-	-	-
STP	Female	2019	MICS	3115	0.3	-	-	-	-
STP	Male	2008-09	DHS	2296	0.3	-	-	-	0.8
STP	Male	2014	MICS	2772	18.3	18.2	-	-	-
STP	Male	2019	MICS	1395	0.1	-	-	-	-
SWZ	Female	2006-07	DHS	4987	0.1	-	-	-	7
SWZ	Female	2010	MICS	4956	5.4	-	-	-	-
SWZ	Female	2014	MICS	5001	4.8	-	-	-	1.2
SWZ	Female	2016-17	PHIA	7413	11.2	-	-	-	-
SWZ	Male	2006-07	DHS	4156	0.1	-	-	-	0.5
SWZ	Male	2010	MICS	4646	10.1	10.1	-	-	-
SWZ	Male	2014	MICS	1629	10.4	10.4	-	-	2.6
SWZ	Male	2016-17	PHIA	5926	20.2	-	-	0.1	-
TCD	Female	1996-97	DHS	7454	0.1	-	-	-	-
TCD	Female	2004	DHS	6085	-	-	-	-	0.5
TCD	Female	2010	MICS	18088	12.8	-	-	0.1	-
TCD	Female	2014-15	DHS	17719	0.3	-	-	-	8
TCD	Male	1996-97	DHS	2320	0.2	-	-	-	-
TCD	Male	2004	DHS	1887	0.2	-	-	-	-
TCD	Male	2014-15	DHS	5248	0.2	-	-	-	3.4
TGO	Female	1998	DHS	8569	-	-	-	0.1	3.6
TGO	Female	2010	MICS	7016	9.3	-	-	-	-
TGO	Female	2013-14	DHS	9480	-	-	-	-	5
TGO	Female	2017	MICS	7657	4.4	-	-	-	-
TGO	Male	2013-14	DHS	4476	0.1	-	-	0.1	0.6
TGO	Male	2017	MICS	2456	6.9	0.4	0.1	-	-
TZA	Female	1991-92	DHS	9238	0.7	-	-	-	5.3
TZA	Female	1996	DHS	8120	0.3	-	-	-	6.8
TZA	Female	1999	DHS	4029	0.1	-	-	-	2.4
TZA	Female	2003-04	DHS	12522	-	-	-	-	0.1
TZA	Female	2004-05	DHS	10329	-	-	-	-	2.6
TZA	Female	2007-08	DHS	16318	-	-	-	-	2.3
TZA	Female	2009-10	DHS	10139	-	-	-	-	2
TZA	Female	2011-12	DHS	19319	0.2	-	-	-	0.6
TZA	Female	2015-16	DHS	13266	-	-	-	-	0.6
TZA	Female	2016-17	PHIA	20699	10.2	-	-	-	-
TZA	Male	1991-92	DHS	2114	0.5	-	-	0.1	0.2
TZA	Male	1996	DHS	2256	0.7	-	-	-	0.1
TZA	Male	1999	DHS	3542	-	-	-	0.1	-
TZA	Male	2004-05	DHS	2635	0.1	-	-	0.1	0.7
TZA	Male	2009-10	DHS	2527	-	-	-	-	0.9
TZA	Male	2015-16	DHS	3514	-	-	-	-	0.3
TZA	Male	2016-17	PHIA	17978	17.6	-	-	-	-
UGA	Female	1995	DHS	7070	0.5	-	-	-	4.6
UGA	Female	2000-01	DHS	7246	0.1	-	-	-	5
UGA	Female	2004-05	AIS	10826	0.4	-	-	0.3	-
UGA	Female	2006	DHS	8531	-	-	-	0.1	2.7
UGA	Female	2011	DHS	30415	-	-	-	-	1.9
UGA	Female	2014	PMA	3986	7.9	-	-	-	3.5

ISO_A3	Sex	Year	Survey	Sample Size	Missing AFS	Missing age/DOB	AFS > age	AFS < 7	'Ever sex' only
UGA	Female	2015	PMA	7748	6.8	2.3	-	0.2	3.4
UGA	Female	2016	DHS	18506	-	-	-	-	2
UGA	Female	2016	PMA	4044	6.9	5.4	-	0.1	5.8
UGA	Female	2016-17	PHIA	19103	12.3	-	-	-	-
UGA	Female	2017	PMA	4363	5.3	4.6	-	0.1	3
UGA	Female	2018	PMA	4454	4.2	3.7	-	0.1	3.7
UGA	Male	1995	DHS	1996	0.2	-	-	-	0.2
UGA	Male	2000-01	DHS	1962	0.1	-	-	-	12.3
UGA	Male	2004-05	AIS	8830	0.1	-	-	0.3	-
UGA	Male	2006	DHS	2503	0.1	-	-	0.1	0.5
UGA	Male	2011	DHS	2295	0.2	-	-	-	2.3
UGA	Male	2016	DHS	5336	-	-	-	-	0.8
UGA	Male	2016-17	PHIA	15611	19.3	-	-	-	-
ZAF	Female	1998	DHS	11735	1.3	-	-	-	5.3
ZAF	Female	2002	HSRC	5361	24.6	-	-	-	-
ZAF	Female	2005	HSRC	10057	1.1	-	0.1	-	-
ZAF	Female	2008	HSRC	22458	29.8	-	0.1	-	-
ZAF	Female	2016	DHS	8514	-	-	-	-	2.9
ZAF	Female	2016-18	HSRC	36692	35	-	-	0.1	0.1
ZAF	Male	2002	HSRC	4427	31.9	-	-	-	-
ZAF	Male	2005	HSRC	6338	1.9	-	0.3	-	-
ZAF	Male	2008	HSRC	20089	38.7	0.1	0.1	-	-
ZAF	Male	2016	DHS	3618	-	-	-	-	0.8
ZAF	Male	2016-18	HSRC	29388	42.5	-	-	0.1	0.1
ZMB	Female	1992	DHS	7060	0.2	-	-	-	3.4
ZMB	Female	1996-97	DHS	8021	0.2	-	-	-	2.9
ZMB	Female	1998	SBS	2046	-	-	0.2	-	-
ZMB	Female	2000-01	SBS	1791	0.1	-	0.3	0.3	-
ZMB	Female	2001-02	DHS	7658	0.2	-	-	-	4.4
ZMB	Female	2003	SBS	2324	0.2	-	-	0.1	-
ZMB	Female	2005	SBS	2174	0.2	-	2.4	-	-
ZMB	Female	2007	DHS	7146	0.1	-	-	-	6.4
ZMB	Female	2009	SBS	2398	0.1	-	0.2	-	-
ZMB	Female	2013-14	DHS	16411	0.2	-	-	-	6.9
ZMB	Female	2016	PHIA	15783	25.5	-	-	-	-
ZMB	Female	2018-19	DHS	13683	-	-	-	-	1.5
ZMB	Male	1996-97	DHS	1849	0.5	-	-	-	0.2
ZMB	Male	1998	SBS	1656	0.1	-	0.2	0.6	-
ZMB	Male	2000-01	SBS	1525	-	-	0.1	0.2	-
ZMB	Male	2001-02	DHS	2145	0.4	-	-	-	0.6
ZMB	Male	2003	SBS	2147	0.2	-	0.1	0.5	-
ZMB	Male	2005	SBS	2046	-	-	1.6	-	-
ZMB	Male	2007	DHS	6500	0.1	-	-	0.1	1
ZMB	Male	2009	SBS	2085	-	-	-	0.1	-
ZMB	Male	2013-14	DHS	14773	0.1	-	-	-	1.1
ZMB	Male	2016	PHIA	14101	36.6	-	-	0.2	-
ZMB	Male	2018-19	DHS	12132	-	-	-	-	0.9
ZWE	Female	1994	DHS	6128	-	-	-	-	5.1
ZWE	Female	1999	DHS	5907	-	-	-	-	6.2

ISO_A3	Sex	Year	Survey	Sample Size	Missing AFS	Missing age/DOB	AFS > age	AFS < 7	'Ever sex' only
ZWE	Female	2005-06	DHS	8907	0.1	-	-	-	3.3
ZWE	Female	2010-11	DHS	9171	-	-	-	-	5.1
ZWE	Female	2014	MICS	15376	6.3	-	-	-	-
ZWE	Female	2015	DHS	9955	-	-	-	-	0.6
ZWE	Female	2015-16	PHIA	15976	11.6	-	-	-	-
ZWE	Female	2019	MICS	10703	5.4	-	-	-	-
ZWE	Male	1994	DHS	2141	0.1	-	-	-	0.2
ZWE	Male	1999	DHS	2609	0.2	-	-	-	-
ZWE	Male	2005-06	DHS	7175	0.3	-	-	-	0.8
ZWE	Male	2010-11	DHS	7480	-	-	-	-	3
ZWE	Male	2014	MICS	9008	12.2	12.1	-	-	-
ZWE	Male	2015	DHS	8396	-	-	-	-	2
ZWE	Male	2015-16	PHIA	13015	22.8	-	-	-	-
ZWE	Male	2019	MICS	4677	10.7	10.7	-	-	-

Supplemental Table S2. Proportion ever had sex by country, sex, and year of birth

ISO_A3 : ISO 3166-1 alpha-3 code for countries
≤ 15, 18 : Cumulative proportion ever had sex by age 15 and 18 years
– : No surveys available for the sex in this country

Annual estimates can be found in file sexual_debut_by_country_age_sex.xlsx.

ISO_A3	Year turned age 15	Male (≤ 15)	Female (≤ 15)	Male (≤ 18)	Female (≤ 18)
AGO	1985	0.39 [0.34-0.44]	0.26 [0.22-0.32]	0.79 [0.75-0.82]	0.77 [0.73-0.80]
AGO	1995	0.38 [0.32-0.44]	0.27 [0.22-0.32]	0.77 [0.73-0.82]	0.77 [0.73-0.80]
AGO	2005	0.37 [0.33-0.40]	0.25 [0.23-0.28]	0.77 [0.74-0.79]	0.76 [0.74-0.78]
AGO	2015	0.35 [0.30-0.42]	0.24 [0.19-0.30]	0.75 [0.70-0.80]	0.75 [0.70-0.79]
BDI	1985	0.08 [0.07-0.10]	0.04 [0.03-0.05]	0.24 [0.20-0.29]	0.25 [0.21-0.28]
BDI	1995	0.07 [0.06-0.08]	0.04 [0.04-0.05]	0.21 [0.19-0.24]	0.27 [0.24-0.29]
BDI	2005	0.06 [0.05-0.06]	0.04 [0.04-0.05]	0.17 [0.16-0.19]	0.26 [0.24-0.27]
BDI	2015	0.05 [0.05-0.06]	0.02 [0.02-0.03]	0.16 [0.14-0.18]	0.18 [0.16-0.21]
BEN	1985	0.24 [0.22-0.27]	0.15 [0.14-0.17]	0.62 [0.59-0.65]	0.57 [0.55-0.60]
BEN	1995	0.21 [0.19-0.22]	0.16 [0.15-0.17]	0.57 [0.54-0.59]	0.58 [0.57-0.60]
BEN	2005	0.16 [0.15-0.17]	0.16 [0.15-0.17]	0.49 [0.47-0.52]	0.58 [0.57-0.60]
BEN	2015	0.09 [0.08-0.10]	0.14 [0.13-0.16]	0.34 [0.31-0.37]	0.55 [0.53-0.58]
BFA	1985	0.05 [0.03-0.06]	0.16 [0.14-0.17]	0.31 [0.26-0.36]	0.67 [0.65-0.69]
BFA	1995	0.04 [0.04-0.05]	0.12 [0.11-0.13]	0.30 [0.27-0.33]	0.60 [0.58-0.62]
BFA	2005	0.03 [0.02-0.04]	0.09 [0.09-0.10]	0.23 [0.20-0.26]	0.54 [0.52-0.55]
BFA	2015	0.01 [0.01-0.03]	0.08 [0.07-0.08]	0.14 [0.09-0.21]	0.49 [0.46-0.51]
BWA	1985	0.09 [0.07-0.12]	0.04 [0.03-0.05]	0.39 [0.34-0.45]	0.36 [0.30-0.40]
BWA	1995	0.09 [0.07-0.10]	0.04 [0.03-0.04]	0.38 [0.35-0.41]	0.34 [0.31-0.36]
BWA	2005	0.05 [0.04-0.06]	0.03 [0.03-0.04]	0.28 [0.24-0.32]	0.31 [0.28-0.35]
BWA	2015	0.03 [0.02-0.05]	0.03 [0.02-0.05]	0.19 [0.11-0.28]	0.28 [0.20-0.39]
CAF	1985	0.15 [0.12-0.18]	0.34 [0.30-0.37]	0.56 [0.50-0.61]	0.80 [0.78-0.83]
CAF	1995	0.15 [0.13-0.18]	0.33 [0.30-0.36]	0.57 [0.53-0.61]	0.80 [0.78-0.82]
CAF	2005	0.15 [0.13-0.17]	0.35 [0.33-0.37]	0.57 [0.53-0.60]	0.81 [0.80-0.82]
CAF	2015	0.14 [0.12-0.16]	0.26 [0.24-0.29]	0.55 [0.51-0.59]	0.75 [0.73-0.77]
CIV	1985	0.27 [0.21-0.33]	0.19 [0.17-0.20]	0.67 [0.61-0.72]	0.67 [0.65-0.69]
CIV	1995	0.19 [0.15-0.22]	0.20 [0.18-0.21]	0.57 [0.53-0.62]	0.68 [0.67-0.70]
CIV	2005	0.12 [0.11-0.14]	0.19 [0.18-0.20]	0.47 [0.45-0.50]	0.68 [0.66-0.69]
CIV	2015	0.11 [0.09-0.13]	0.16 [0.14-0.17]	0.45 [0.41-0.49]	0.63 [0.61-0.66]
CMR	1985	0.22 [0.19-0.25]	0.34 [0.31-0.36]	0.62 [0.59-0.64]	0.80 [0.78-0.81]
CMR	1995	0.17 [0.16-0.19]	0.24 [0.22-0.26]	0.56 [0.54-0.58]	0.72 [0.71-0.74]
CMR	2005	0.12 [0.11-0.13]	0.16 [0.15-0.18]	0.48 [0.46-0.50]	0.64 [0.62-0.65]
CMR	2015	0.08 [0.07-0.10]	0.13 [0.12-0.14]	0.40 [0.37-0.43]	0.58 [0.56-0.60]
COD	1985	0.12 [0.10-0.14]	0.17 [0.16-0.18]	0.46 [0.42-0.50]	0.60 [0.57-0.61]
COD	1995	0.16 [0.14-0.18]	0.19 [0.18-0.20]	0.53 [0.51-0.57]	0.62 [0.61-0.64]
COD	2005	0.22 [0.20-0.24]	0.21 [0.20-0.21]	0.62 [0.59-0.64]	0.64 [0.63-0.65]
COD	2015	0.21 [0.19-0.24]	0.22 [0.21-0.23]	0.61 [0.58-0.64]	0.66 [0.65-0.68]
COG	1985	0.39 [0.34-0.45]	0.38 [0.34-0.41]	0.81 [0.78-0.84]	0.86 [0.84-0.87]
COG	1995	0.35 [0.32-0.39]	0.30 [0.28-0.32]	0.78 [0.76-0.81]	0.82 [0.80-0.83]
COG	2005	0.27 [0.25-0.29]	0.26 [0.24-0.28]	0.72 [0.69-0.74]	0.78 [0.77-0.80]

COG	2015	0.20 [0.17-0.25]	0.20 [0.18-0.23]	0.64 [0.58-0.69]	0.73 [0.70-0.76]
COM	1985	0.14 [0.11-0.18]	0.16 [0.12-0.20]	0.43 [0.38-0.49]	0.41 [0.36-0.46]
COM	1995	0.15 [0.12-0.18]	0.13 [0.10-0.16]	0.44 [0.39-0.50]	0.36 [0.32-0.41]
COM	2005	0.14 [0.11-0.16]	0.11 [0.09-0.12]	0.43 [0.39-0.47]	0.33 [0.30-0.36]
COM	2015	0.11 [0.07-0.16]	0.08 [0.05-0.12]	0.38 [0.30-0.47]	0.27 [0.19-0.36]
ERI	1985	0.03 [0.02-0.05]	0.25 [0.20-0.30]	0.18 [0.14-0.22]	0.53 [0.48-0.59]
ERI	1995	0.03 [0.02-0.03]	0.18 [0.14-0.22]	0.15 [0.12-0.18]	0.45 [0.40-0.51]
ERI	2005	0.02 [0.01-0.03]	0.10 [0.08-0.13]	0.13 [0.11-0.15]	0.34 [0.30-0.38]
ERI	2015	0.02 [0.01-0.03]	0.07 [0.03-0.14]	0.11 [0.07-0.18]	0.28 [0.17-0.40]
ETH	1985	0.05 [0.04-0.06]	0.23 [0.22-0.24]	0.24 [0.21-0.26]	0.56 [0.54-0.57]
ETH	1995	0.04 [0.03-0.04]	0.18 [0.17-0.19]	0.20 [0.19-0.22]	0.50 [0.48-0.51]
ETH	2005	0.03 [0.02-0.03]	0.12 [0.12-0.13]	0.17 [0.15-0.18]	0.41 [0.40-0.42]
ETH	2015	0.02 [0.02-0.03]	0.11 [0.10-0.11]	0.15 [0.12-0.17]	0.38 [0.36-0.39]
GAB	1985	0.43 [0.39-0.48]	0.33 [0.30-0.37]	0.82 [0.79-0.85]	0.85 [0.83-0.87]
GAB	1995	0.41 [0.37-0.45]	0.25 [0.23-0.28]	0.81 [0.78-0.83]	0.80 [0.78-0.82]
GAB	2005	0.33 [0.30-0.36]	0.20 [0.18-0.22]	0.75 [0.73-0.78]	0.75 [0.73-0.78]
GAB	2015	0.22 [0.16-0.28]	0.16 [0.12-0.22]	0.64 [0.56-0.71]	0.70 [0.62-0.77]
GHA	1985	0.07 [0.06-0.08]	0.10 [0.09-0.11]	0.31 [0.28-0.34]	0.44 [0.41-0.46]
GHA	1995	0.07 [0.06-0.08]	0.10 [0.09-0.10]	0.30 [0.28-0.33]	0.43 [0.41-0.45]
GHA	2005	0.07 [0.06-0.08]	0.10 [0.09-0.10]	0.31 [0.29-0.34]	0.44 [0.42-0.46]
GHA	2015	0.08 [0.07-0.09]	0.11 [0.10-0.12]	0.33 [0.30-0.37]	0.46 [0.43-0.48]
GIN	1985	0.23 [0.20-0.27]	0.37 [0.34-0.40]	0.60 [0.56-0.65]	0.78 [0.76-0.79]
GIN	1995	0.18 [0.16-0.21]	0.31 [0.29-0.34]	0.54 [0.51-0.58]	0.74 [0.72-0.76]
GIN	2005	0.10 [0.09-0.12]	0.26 [0.24-0.27]	0.40 [0.37-0.43]	0.70 [0.68-0.71]
GIN	2015	0.05 [0.04-0.06]	0.17 [0.16-0.19]	0.26 [0.22-0.29]	0.60 [0.58-0.62]
GMB	1985	0.04 [0.03-0.06]	0.16 [0.14-0.18]	0.17 [0.13-0.21]	0.51 [0.48-0.54]
GMB	1995	0.05 [0.04-0.07]	0.14 [0.12-0.15]	0.19 [0.16-0.23]	0.47 [0.45-0.50]
GMB	2005	0.06 [0.06-0.07]	0.10 [0.09-0.11]	0.22 [0.20-0.24]	0.41 [0.39-0.43]
GMB	2015	0.08 [0.07-0.09]	0.06 [0.05-0.07]	0.27 [0.24-0.29]	0.31 [0.29-0.33]
GNB	1985	0.09 [0.05-0.13]	0.23 [0.17-0.29]	0.42 [0.31-0.53]	0.79 [0.73-0.83]
GNB	1995	0.13 [0.09-0.17]	0.20 [0.16-0.23]	0.51 [0.43-0.58]	0.76 [0.72-0.79]
GNB	2005	0.15 [0.13-0.17]	0.20 [0.18-0.22]	0.55 [0.52-0.59]	0.76 [0.74-0.78]
GNB	2015	0.09 [0.07-0.11]	0.13 [0.11-0.15]	0.42 [0.37-0.48]	0.67 [0.63-0.70]
KEN	1985	0.25 [0.22-0.28]	0.13 [0.12-0.14]	0.58 [0.54-0.61]	0.47 [0.45-0.49]
KEN	1995	0.25 [0.23-0.26]	0.13 [0.12-0.14]	0.57 [0.55-0.60]	0.47 [0.46-0.49]
KEN	2005	0.23 [0.22-0.25]	0.14 [0.13-0.15]	0.56 [0.54-0.58]	0.50 [0.49-0.51]
KEN	2015	0.18 [0.16-0.21]	0.15 [0.14-0.16]	0.48 [0.43-0.52]	0.51 [0.49-0.53]
LBR	1985	0.13 [0.10-0.16]	0.16 [0.13-0.20]	0.59 [0.53-0.65]	0.78 [0.74-0.82]
LBR	1995	0.12 [0.10-0.14]	0.20 [0.17-0.23]	0.57 [0.53-0.61]	0.82 [0.80-0.84]
LBR	2005	0.12 [0.11-0.14]	0.26 [0.23-0.28]	0.59 [0.55-0.62]	0.86 [0.84-0.87]
LBR	2015	0.11 [0.07-0.14]	0.24 [0.19-0.30]	0.55 [0.46-0.63]	0.85 [0.81-0.88]
LSO	1985	0.10 [0.08-0.13]	0.08 [0.07-0.09]	0.44 [0.39-0.48]	0.50 [0.46-0.54]
LSO	1995	0.15 [0.13-0.17]	0.06 [0.05-0.07]	0.51 [0.48-0.54]	0.45 [0.42-0.48]
LSO	2005	0.20 [0.18-0.22]	0.06 [0.05-0.06]	0.58 [0.55-0.60]	0.43 [0.41-0.46]
LSO	2015	0.15 [0.13-0.18]	0.04 [0.03-0.05]	0.52 [0.49-0.56]	0.36 [0.33-0.40]
MDG	1985	0.09 [0.08-0.11]	0.11 [0.10-0.13]	0.55 [0.50-0.58]	0.53 [0.49-0.56]
MDG	1995	0.10 [0.09-0.11]	0.17 [0.15-0.19]	0.56 [0.53-0.59]	0.63 [0.60-0.65]
MDG	2005	0.10 [0.09-0.12]	0.20 [0.18-0.23]	0.57 [0.54-0.60]	0.68 [0.65-0.70]
MDG	2015	0.12 [0.11-0.14]	0.26 [0.23-0.29]	0.61 [0.58-0.65]	0.73 [0.70-0.75]
MLI	1985	0.10 [0.09-0.12]	0.39 [0.37-0.40]	0.41 [0.37-0.44]	0.81 [0.80-0.82]
MLI	1995	0.07 [0.06-0.08]	0.29 [0.28-0.31]	0.33 [0.30-0.35]	0.75 [0.74-0.76]

MLI	2005	0.05 [0.04-0.06]	0.22 [0.21-0.23]	0.26 [0.24-0.28]	0.68 [0.67-0.69]
MLI	2015	0.04 [0.03-0.05]	0.17 [0.16-0.18]	0.22 [0.20-0.26]	0.62 [0.61-0.64]
MOZ	1985	0.20 [0.17-0.24]	0.36 [0.33-0.38]	0.69 [0.65-0.74]	0.82 [0.80-0.83]
MOZ	1995	0.22 [0.19-0.25]	0.28 [0.27-0.30]	0.71 [0.68-0.74]	0.76 [0.75-0.78]
MOZ	2005	0.20 [0.18-0.22]	0.26 [0.24-0.27]	0.69 [0.66-0.72]	0.74 [0.73-0.75]
MOZ	2015	0.18 [0.14-0.21]	0.22 [0.20-0.25]	0.66 [0.61-0.70]	0.71 [0.68-0.74]
MWI	1985	0.21 [0.19-0.23]	0.27 [0.26-0.28]	0.58 [0.55-0.60]	0.75 [0.74-0.76]
MWI	1995	0.18 [0.17-0.20]	0.20 [0.20-0.21]	0.53 [0.51-0.55]	0.68 [0.67-0.69]
MWI	2005	0.16 [0.15-0.17]	0.15 [0.15-0.16]	0.49 [0.47-0.51]	0.61 [0.60-0.62]
MWI	2015	0.14 [0.13-0.15]	0.12 [0.11-0.13]	0.45 [0.43-0.48]	0.54 [0.53-0.56]
NAM	1985	0.24 [0.21-0.27]	0.09 [0.08-0.10]	0.65 [0.62-0.68]	0.48 [0.45-0.50]
NAM	1995	0.23 [0.21-0.26]	0.10 [0.09-0.11]	0.65 [0.62-0.67]	0.50 [0.48-0.52]
NAM	2005	0.17 [0.15-0.18]	0.08 [0.07-0.09]	0.56 [0.54-0.58]	0.46 [0.44-0.48]
NAM	2015	0.11 [0.09-0.13]	0.08 [0.07-0.09]	0.45 [0.42-0.49]	0.46 [0.44-0.49]
NER	1985	0.08 [0.06-0.11]	0.53 [0.50-0.56]	0.32 [0.26-0.39]	0.83 [0.81-0.84]
NER	1995	0.04 [0.03-0.05]	0.36 [0.34-0.38]	0.20 [0.17-0.23]	0.72 [0.71-0.74]
NER	2005	0.02 [0.02-0.02]	0.20 [0.19-0.21]	0.11 [0.09-0.13]	0.59 [0.57-0.60]
NER	2015	0.01 [0.01-0.02]	0.09 [0.07-0.10]	0.06 [0.03-0.09]	0.42 [0.39-0.44]
NGA	1985	0.15 [0.13-0.16]	0.26 [0.25-0.27]	0.43 [0.41-0.46]	0.62 [0.61-0.64]
NGA	1995	0.10 [0.09-0.11]	0.20 [0.19-0.21]	0.33 [0.31-0.35]	0.56 [0.55-0.57]
NGA	2005	0.06 [0.05-0.06]	0.18 [0.18-0.19]	0.23 [0.22-0.24]	0.53 [0.52-0.54]
NGA	2015	0.03 [0.02-0.03]	0.14 [0.13-0.15]	0.13 [0.11-0.14]	0.46 [0.45-0.48]
RWA	1985	0.10 [0.09-0.11]	0.05 [0.05-0.05]	0.30 [0.27-0.32]	0.25 [0.23-0.26]
RWA	1995	0.08 [0.08-0.09]	0.04 [0.04-0.04]	0.26 [0.24-0.28]	0.22 [0.21-0.23]
RWA	2005	0.08 [0.07-0.08]	0.03 [0.03-0.03]	0.24 [0.22-0.25]	0.17 [0.17-0.18]
RWA	2015	0.08 [0.07-0.09]	0.05 [0.04-0.05]	0.25 [0.23-0.27]	0.24 [0.23-0.25]
SDN	1985	0.04 [0.03-0.05]	-	0.17 [0.14-0.19]	-
SDN	1995	0.04 [0.03-0.05]	-	0.15 [0.13-0.17]	-
SDN	2005	0.03 [0.03-0.04]	-	0.14 [0.12-0.16]	-
SDN	2015	0.04 [0.02-0.06]	-	0.15 [0.10-0.21]	-
SEN	1985	0.21 [0.18-0.23]	0.24 [0.22-0.25]	0.47 [0.43-0.50]	0.56 [0.54-0.57]
SEN	1995	0.14 [0.13-0.16]	0.15 [0.14-0.16]	0.37 [0.34-0.39]	0.44 [0.43-0.46]
SEN	2005	0.06 [0.06-0.07]	0.12 [0.12-0.13]	0.21 [0.20-0.23]	0.41 [0.39-0.42]
SEN	2015	0.03 [0.02-0.03]	0.10 [0.09-0.11]	0.11 [0.10-0.12]	0.36 [0.34-0.37]
SLE	1985	0.11 [0.10-0.14]	0.24 [0.22-0.27]	0.52 [0.47-0.57]	0.74 [0.71-0.76]
SLE	1995	0.11 [0.10-0.13]	0.26 [0.24-0.27]	0.52 [0.48-0.56]	0.75 [0.73-0.77]
SLE	2005	0.11 [0.10-0.12]	0.23 [0.22-0.25]	0.50 [0.48-0.53]	0.73 [0.71-0.74]
SLE	2015	0.07 [0.06-0.08]	0.18 [0.17-0.19]	0.40 [0.37-0.43]	0.65 [0.63-0.67]
SSD	1985	-	0.14 [0.12-0.18]	-	0.57 [0.52-0.61]
SSD	1995	-	0.17 [0.14-0.20]	-	0.60 [0.56-0.64]
SSD	2005	-	0.17 [0.15-0.20]	-	0.61 [0.58-0.64]
SSD	2015	-	0.16 [0.11-0.23]	-	0.59 [0.51-0.68]
STP	1985	0.19 [0.15-0.24]	0.09 [0.07-0.12]	0.62 [0.56-0.69]	0.53 [0.46-0.59]
STP	1995	0.18 [0.15-0.21]	0.11 [0.08-0.13]	0.61 [0.56-0.65]	0.57 [0.52-0.62]
STP	2005	0.17 [0.15-0.19]	0.14 [0.12-0.17]	0.60 [0.57-0.63]	0.64 [0.60-0.68]
STP	2015	0.15 [0.13-0.18]	0.11 [0.08-0.13]	0.57 [0.51-0.62]	0.57 [0.52-0.63]
SWZ	1985	0.08 [0.06-0.10]	0.21 [0.17-0.24]	0.38 [0.33-0.43]	0.69 [0.65-0.72]
SWZ	1995	0.07 [0.06-0.08]	0.10 [0.09-0.12]	0.35 [0.32-0.39]	0.54 [0.51-0.57]
SWZ	2005	0.05 [0.04-0.06]	0.05 [0.04-0.05]	0.30 [0.28-0.33]	0.39 [0.37-0.42]
SWZ	2015	0.05 [0.04-0.07]	0.03 [0.02-0.03]	0.31 [0.27-0.36]	0.30 [0.25-0.34]
TCD	1985	0.23 [0.19-0.27]	0.35 [0.32-0.37]	0.56 [0.52-0.61]	0.76 [0.74-0.77]

TCD	1995	0.15 [0.13-0.18]	0.31 [0.30-0.33]	0.46 [0.42-0.49]	0.74 [0.72-0.75]
TCD	2005	0.08 [0.07-0.10]	0.32 [0.31-0.34]	0.33 [0.31-0.36]	0.74 [0.73-0.75]
TCD	2015	0.04 [0.04-0.05]	0.33 [0.31-0.35]	0.21 [0.19-0.24]	0.75 [0.73-0.76]
TGO	1985	0.07 [0.04-0.10]	0.11 [0.09-0.14]	0.29 [0.21-0.38]	0.50 [0.45-0.56]
TGO	1995	0.08 [0.06-0.11]	0.12 [0.10-0.14]	0.33 [0.27-0.40]	0.52 [0.48-0.56]
TGO	2005	0.09 [0.08-0.10]	0.13 [0.12-0.14]	0.35 [0.32-0.38]	0.54 [0.51-0.56]
TGO	2015	0.11 [0.09-0.14]	0.13 [0.11-0.15]	0.41 [0.35-0.47]	0.54 [0.50-0.58]
TZA	1985	0.08 [0.07-0.10]	0.12 [0.11-0.13]	0.34 [0.30-0.38]	0.51 [0.49-0.53]
TZA	1995	0.10 [0.09-0.12]	0.12 [0.11-0.13]	0.40 [0.37-0.43]	0.50 [0.49-0.51]
TZA	2005	0.12 [0.11-0.13]	0.11 [0.10-0.11]	0.44 [0.42-0.46]	0.47 [0.46-0.49]
TZA	2015	0.19 [0.17-0.21]	0.17 [0.15-0.18]	0.56 [0.52-0.59]	0.58 [0.56-0.60]
UGA	1985	0.15 [0.14-0.17]	0.21 [0.20-0.22]	0.53 [0.49-0.56]	0.67 [0.66-0.69]
UGA	1995	0.13 [0.12-0.14]	0.19 [0.18-0.20]	0.47 [0.45-0.50]	0.64 [0.63-0.66]
UGA	2005	0.13 [0.12-0.14]	0.14 [0.13-0.15]	0.49 [0.47-0.51]	0.57 [0.56-0.58]
UGA	2015	0.15 [0.13-0.17]	0.16 [0.15-0.17]	0.51 [0.48-0.55]	0.61 [0.59-0.62]
ZAF	1985	0.07 [0.06-0.08]	0.03 [0.03-0.03]	0.32 [0.29-0.34]	0.27 [0.25-0.29]
ZAF	1995	0.10 [0.09-0.11]	0.05 [0.04-0.05]	0.40 [0.38-0.42]	0.34 [0.32-0.36]
ZAF	2005	0.14 [0.13-0.16]	0.07 [0.06-0.07]	0.48 [0.46-0.50]	0.41 [0.39-0.42]
ZAF	2015	0.15 [0.13-0.17]	0.06 [0.05-0.06]	0.49 [0.47-0.52]	0.37 [0.35-0.40]
ZMB	1985	0.22 [0.20-0.24]	0.17 [0.15-0.18]	0.58 [0.56-0.61]	0.62 [0.60-0.64]
ZMB	1995	0.19 [0.18-0.21]	0.17 [0.16-0.18]	0.54 [0.52-0.56]	0.62 [0.61-0.63]
ZMB	2005	0.16 [0.15-0.17]	0.14 [0.13-0.15]	0.49 [0.47-0.51]	0.58 [0.57-0.60]
ZMB	2015	0.18 [0.17-0.20]	0.15 [0.13-0.16]	0.52 [0.50-0.55]	0.59 [0.57-0.61]
ZWE	1985	0.06 [0.05-0.07]	0.11 [0.10-0.12]	0.33 [0.30-0.36]	0.52 [0.50-0.55]
ZWE	1995	0.06 [0.05-0.06]	0.07 [0.06-0.07]	0.30 [0.28-0.32]	0.42 [0.40-0.44]
ZWE	2005	0.05 [0.05-0.05]	0.05 [0.05-0.06]	0.28 [0.27-0.30]	0.38 [0.37-0.40]
ZWE	2015	0.06 [0.05-0.07]	0.05 [0.05-0.06]	0.32 [0.29-0.35]	0.38 [0.36-0.40]

Supplemental Table S3. Parameter estimates by country and sex

The probability density distribution can be generated from R using commands

```
remotes::install_github("kklot/ktools")
dskewlogis(ages, scale, shape, skew)
```

The estimates are for the birth cohort 2000, assuming the respondent's age at interview is 23. The numbers inside bracket are 95% uncertainty interval.

Sex	ISO_A3	Scale	Shape	Skew
Male	AGO	0.06144 [0.05966-0.06334]	9.809 [9.401-10.277]	0.8868 [0.8122-0.9651]
Female	AGO	0.06621 [0.06472-0.06764]	10.450 [10.208-10.714]	1.9423 [1.7961-2.1047]
Male	BDI	0.04196 [0.04088-0.04295]	8.836 [8.580- 9.092]	0.7173 [0.6805-0.7582]
Female	BDI	0.05620 [0.05517-0.05720]	6.834 [6.735- 6.929]	2.5787 [2.4554-2.7290]
Male	BEN	0.05155 [0.05070-0.05243]	8.915 [8.732- 9.108]	0.9966 [0.9490-1.0436]
Female	BEN	0.05632 [0.05581-0.05687]	11.140 [11.004-11.286]	0.9584 [0.9319-0.9863]
Male	BFA	0.05150 [0.04920-0.05370]	8.738 [8.448- 8.988]	1.7971 [1.6325-1.9847]
Female	BFA	0.05646 [0.05600-0.05694]	12.158 [12.000-12.310]	1.2064 [1.1694-1.2497]
Male	BWA	0.05129 [0.04839-0.05387]	8.146 [7.916- 8.397]	1.5573 [1.4341-1.6768]
Female	BWA	0.05332 [0.05142-0.05543]	11.915 [11.629-12.225]	1.3158 [1.2369-1.4025]
Male	CAF	0.05730 [0.05626-0.05837]	10.515 [10.190-10.871]	1.1051 [1.0188-1.1950]
Female	CAF	0.06274 [0.06194-0.06350]	10.943 [10.724-11.165]	1.2363 [1.1719-1.3014]
Male	CIV	0.05966 [0.05843-0.06086]	8.013 [7.853- 8.205]	1.7769 [1.6530-1.8992]
Female	CIV	0.06125 [0.06064-0.06183]	10.545 [10.398-10.685]	1.4878 [1.4303-1.5463]
Male	CMR	0.06021 [0.05924-0.06118]	7.719 [7.599- 7.848]	2.1342 [2.0065-2.2666]
Female	CMR	0.06129 [0.06077-0.06183]	10.036 [9.921-10.159]	1.7052 [1.6407-1.7662]
Male	COD	0.06295 [0.06178-0.06411]	8.236 [8.062- 8.420]	1.6221 [1.5269-1.7275]
Female	COD	0.06301 [0.06251-0.06354]	9.259 [9.166- 9.352]	1.5266 [1.4834-1.5705]
Male	COG	0.06024 [0.05873-0.06181]	9.930 [9.681-10.210]	1.2087 [1.1279-1.2865]
Female	COG	0.06165 [0.06082-0.06246]	12.020 [11.833-12.200]	1.2586 [1.2124-1.3086]
Male	COM	0.05956 [0.05615-0.06325]	6.480 [6.094- 6.861]	1.9575 [1.6375-2.4016]
Female	COM	0.06696 [0.06171-0.07261]	4.679 [4.478- 4.875]	3.7076 [3.0301-4.5790]
Male	ERI	0.05585 [0.05179-0.06049]	5.364 [5.124- 5.644]	3.1957 [2.5671-3.9282]
Female	ERI	0.06903 [0.06279-0.07578]	4.891 [4.754- 5.052]	4.3119 [3.5718-5.1627]
Male	ETH	0.05198 [0.05084-0.05319]	6.648 [6.545- 6.765]	2.0375 [1.9099-2.1555]
Female	ETH	0.06235 [0.06164-0.06306]	6.127 [6.076- 6.179]	2.4408 [2.3697-2.5151]
Male	GAB	0.06044 [0.05820-0.06249]	9.570 [9.225- 9.897]	1.2126 [1.1086-1.3292]
Female	GAB	0.06009 [0.05854-0.06170]	13.066 [12.747-13.402]	1.1509 [1.0755-1.2286]
Male	GHA	0.05346 [0.05246-0.05459]	8.368 [8.187- 8.552]	1.2789 [1.2149-1.3498]
Female	GHA	0.05599 [0.05543-0.05662]	9.890 [9.772-10.016]	1.1927 [1.1548-1.2301]
Male	GIN	0.05337 [0.05214-0.05473]	7.778 [7.533- 8.015]	1.5736 [1.4381-1.7263]
Female	GIN	0.06622 [0.06535-0.06716]	8.412 [8.288- 8.541]	2.4490 [2.3039-2.6052]
Male	GMB	0.05130 [0.04987-0.05275]	6.454 [6.234- 6.673]	1.3440 [1.2336-1.4617]
Female	GMB	0.06103 [0.06008-0.06198]	6.692 [6.609- 6.784]	2.7304 [2.5984-2.8821]
Male	GNB	0.05560 [0.05405-0.05713]	9.799 [9.410-10.219]	1.2411 [1.1185-1.3766]
Female	GNB	0.06064 [0.05991-0.06134]	12.616 [12.392-12.858]	1.4185 [1.3465-1.4929]
Male	KEN	0.05441 [0.05312-0.05577]	7.943 [7.797- 8.084]	0.9489 [0.9151-0.9876]
Female	KEN	0.05759 [0.05711-0.05805]	8.972 [8.886- 9.053]	1.2285 [1.1985-1.2570]
Male	LBR	0.05808 [0.05627-0.05975]	11.400 [11.047-11.738]	1.2746 [1.1770-1.3865]
Female	LBR	0.06416 [0.06294-0.06536]	14.470 [14.157-14.815]	1.4229 [1.3333-1.5187]
Male	LSO	0.06859 [0.06703-0.07022]	6.959 [6.804- 7.112]	3.1128 [2.8091-3.4291]

Sex	ISO_A3	Scale	Shape	Skew
Female	LSO	0.05747 [0.05662-0.05835]	10.134 [9.961-10.316]	1.8845 [1.7792-1.9936]
Male	MDG	0.05977 [0.05894-0.06059]	11.675 [11.405-11.956]	1.3784 [1.2929-1.4524]
Female	MDG	0.06449 [0.06361-0.06528]	10.005 [9.862-10.147]	1.5449 [1.4867-1.6066]
Male	MLI	0.05316 [0.05207-0.05426]	7.740 [7.575- 7.908]	1.7005 [1.5971-1.8240]
Female	MLI	0.06333 [0.06284-0.06389]	9.344 [9.261- 9.423]	1.8333 [1.7858-1.8860]
Male	MOZ	0.06002 [0.05872-0.06124]	11.218 [10.895-11.521]	1.2065 [1.1222-1.3026]
Female	MOZ	0.06204 [0.06127-0.06292]	10.768 [10.606-10.939]	1.2926 [1.2410-1.3477]
Male	MWI	0.05424 [0.05359-0.05493]	8.956 [8.815- 9.102]	0.9820 [0.9459-1.0169]
Female	MWI	0.05757 [0.05724-0.05790]	10.970 [10.879-11.060]	1.1881 [1.1647-1.2135]
Male	NAM	0.05907 [0.05800-0.06014]	8.389 [8.204- 8.571]	1.6809 [1.5805-1.7905]
Female	NAM	0.06206 [0.06117-0.06289]	8.746 [8.617- 8.889]	2.3921 [2.2597-2.5345]
Male	NER	0.04325 [0.04098-0.04572]	7.492 [7.184- 7.822]	1.4280 [1.2766-1.6069]
Female	NER	0.06887 [0.06755-0.07022]	6.797 [6.698- 6.890]	4.1789 [3.8860-4.4995]
Male	NGA	0.04777 [0.04706-0.04852]	6.657 [6.561- 6.737]	1.5690 [1.5155-1.6345]
Female	NGA	0.06107 [0.06063-0.06154]	7.154 [7.108- 7.196]	1.8696 [1.8337-1.9090]
Male	RWA	0.04672 [0.04611-0.04738]	8.173 [8.012- 8.335]	0.8546 [0.8213-0.8909]
Female	RWA	0.05187 [0.05135-0.05237]	8.143 [8.055- 8.236]	1.4142 [1.3725-1.4541]
Male	SDN	0.05789 [0.05281-0.06321]	4.422 [4.220- 4.658]	3.1672 [2.5597-3.9256]
Male	SEN	0.04662 [0.04556-0.04777]	5.515 [5.411- 5.621]	1.6979 [1.6083-1.8078]
Female	SEN	0.06596 [0.06511-0.06679]	5.681 [5.632- 5.733]	3.2001 [3.0684-3.3223]
Male	SLE	0.05408 [0.05336-0.05476]	11.392 [11.151-11.616]	1.0549 [1.0058-1.1077]
Female	SLE	0.05833 [0.05785-0.05878]	12.055 [11.930-12.202]	0.9570 [0.9332-0.9803]
Female	SSD	0.06376 [0.06131-0.06636]	8.805 [8.547- 9.087]	1.9904 [1.8042-2.1964]
Male	STP	0.05786 [0.05652-0.05921]	10.323 [9.916-10.722]	1.1257 [1.0360-1.2314]
Female	STP	0.05842 [0.05727-0.05959]	11.896 [11.487-12.270]	1.2748 [1.1685-1.3863]
Male	SWZ	0.05682 [0.05549-0.05817]	8.080 [7.861- 8.298]	1.9013 [1.7531-2.0621]
Female	SWZ	0.05756 [0.05648-0.05869]	9.449 [9.261- 9.667]	2.2712 [2.1002-2.4472]
Male	TCD	0.05505 [0.05355-0.05662]	6.421 [6.242- 6.602]	2.1292 [1.9258-2.3413]
Female	TCD	0.06898 [0.06827-0.06968]	8.527 [8.434- 8.629]	1.9760 [1.9095-2.0510]
Male	TGO	0.05384 [0.05228-0.05553]	9.303 [8.921- 9.674]	1.0479 [0.9590-1.1414]
Female	TGO	0.05709 [0.05611-0.05804]	10.785 [10.557-11.012]	1.1132 [1.0585-1.1742]
Male	TZA	0.05916 [0.05809-0.06021]	8.502 [8.341- 8.681]	1.2660 [1.2019-1.3288]
Female	TZA	0.06304 [0.06251-0.06361]	8.572 [8.495- 8.647]	1.8681 [1.8179-1.9205]
Male	UGA	0.05720 [0.05642-0.05810]	9.268 [9.121- 9.425]	1.1711 [1.1246-1.2178]
Female	UGA	0.05988 [0.05946-0.06032]	10.246 [10.148-10.339]	1.3173 [1.2881-1.3486]
Male	ZAF	0.06162 [0.06075-0.06248]	7.371 [7.272- 7.466]	1.8528 [1.7817-1.9232]
Female	ZAF	0.05959 [0.05894-0.06024]	8.524 [8.428- 8.616]	2.2448 [2.1683-2.3206]
Male	ZMB	0.05694 [0.05618-0.05765]	8.457 [8.329- 8.576]	1.0851 [1.0483-1.1241]
Female	ZMB	0.06255 [0.06199-0.06313]	9.385 [9.278- 9.487]	1.8533 [1.7859-1.9234]
Male	ZWE	0.05479 [0.05404-0.05553]	8.614 [8.482- 8.741]	1.5059 [1.4459-1.5723]
Female	ZWE	0.05892 [0.05830-0.05956]	8.986 [8.881- 9.095]	2.0978 [2.0140-2.1883]

Supplemental Table S4. ISO A3 and country names of sub-Saharan Africa

Name	ISO_A3	Subregion
Angola	AGO	Middle Africa
Benin	BEN	Western Africa
Botswana	BWA	Southern Africa
Burkina Faso	BFA	Western Africa
Burundi	BDI	Eastern Africa
Cabo Verde*	CPV	Western Africa
Cameroon	CMR	Middle Africa
Central African Republic	CAF	Middle Africa
Chad	TCD	Middle Africa
Comoros	COM	Eastern Africa
Congo	COG	Middle Africa
Congo, Democratic Republic of the	COD	Middle Africa
Cote d'Ivoire	CIV	Western Africa
Djibouti*	DJI	Eastern Africa
Equatorial Guinea*	GNQ	Middle Africa
Eritrea	ERI	Eastern Africa
Eswatini	SWZ	Southern Africa
Ethiopia	ETH	Eastern Africa
Gabon	GAB	Middle Africa
Gambia	GMB	Western Africa
Ghana	GHA	Western Africa
Guinea	GIN	Western Africa
Guinea-Bissau	GNB	Western Africa
Kenya	KEN	Eastern Africa
Lesotho	LSO	Southern Africa
Liberia	LBR	Western Africa
Madagascar	MDG	Eastern Africa
Malawi	MWI	Eastern Africa
Mali	MLI	Western Africa
Mauritania*	MRT	Western Africa
Mauritius*	MUS	Eastern Africa
Mozambique	MOZ	Eastern Africa
Namibia	NAM	Southern Africa
Niger	NER	Western Africa
Nigeria	NGA	Western Africa
Rwanda	RWA	Eastern Africa
Sao Tome and Principe	STP	Middle Africa
Senegal	SEN	Western Africa
Seychelles*	SYC	Eastern Africa
Sierra Leone	SLE	Western Africa
Somalia*	SOM	Eastern Africa
South Africa	ZAF	Southern Africa
South Sudan	SSD	Eastern Africa
Sudan	SDN	Eastern Africa
Tanzania, United Republic of	TZA	Eastern Africa
Togo	TGO	Western Africa
Uganda	UGA	Eastern Africa
Zambia	ZMB	Eastern Africa
Zimbabwe	ZWE	Eastern Africa

* Omitted from analysis due to no available surveys.
