# Supplementary material for: Trends and country-level variation in age at first sex in sub-Saharan Africa among birth cohorts entering adulthood between 1985 and 2020
Source: BMC Public Health. 2022 Jun 4;22:1120. doi: 10.1186/s12889-022-13451-y (PMC9167540; doi:10.1186/s12889-022-13451-y)
Supplement: Supplementary file 2 — Additional file 2. [file 12889_2022_13451_MOESM2_ESM.pdf]

Angola - male

Data and model's prediction

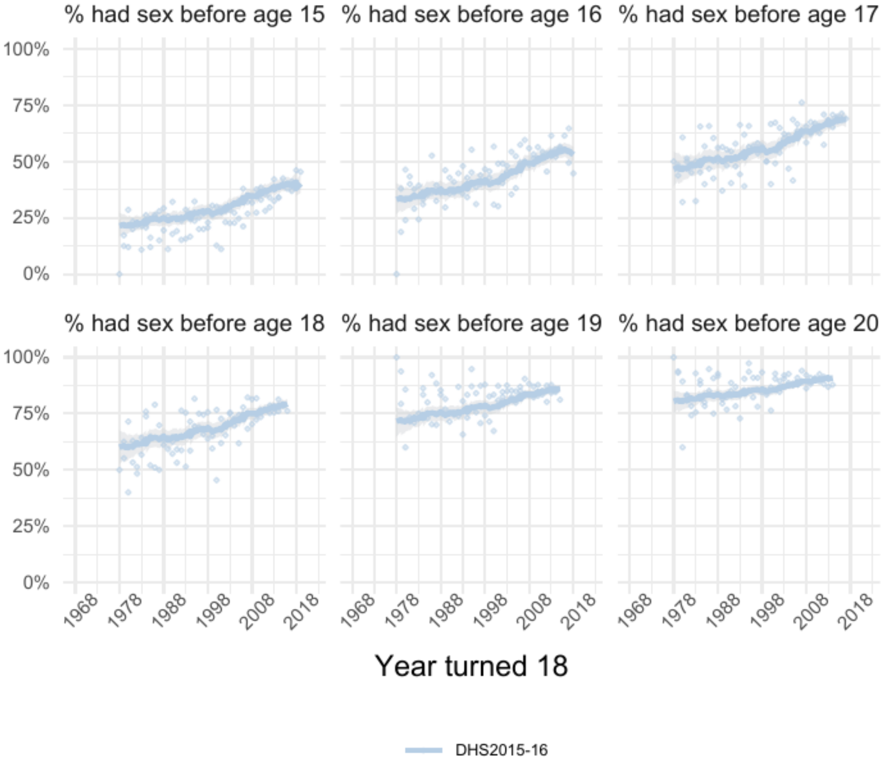

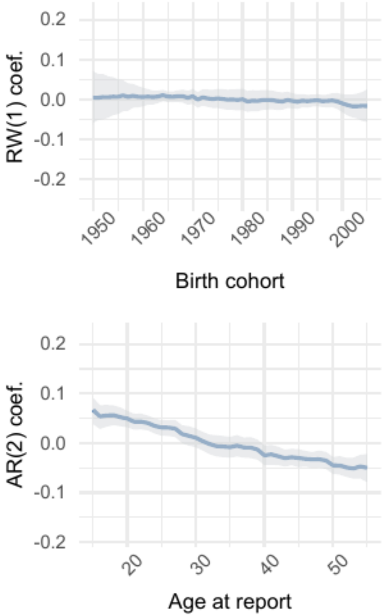

	Parameter	Estimate
1	intercept	0.08 [0.07 – 0.10]
2	skew	0.89 [0.81 – 0.97]
3	shape	9.81 [9.40 – 10.28]

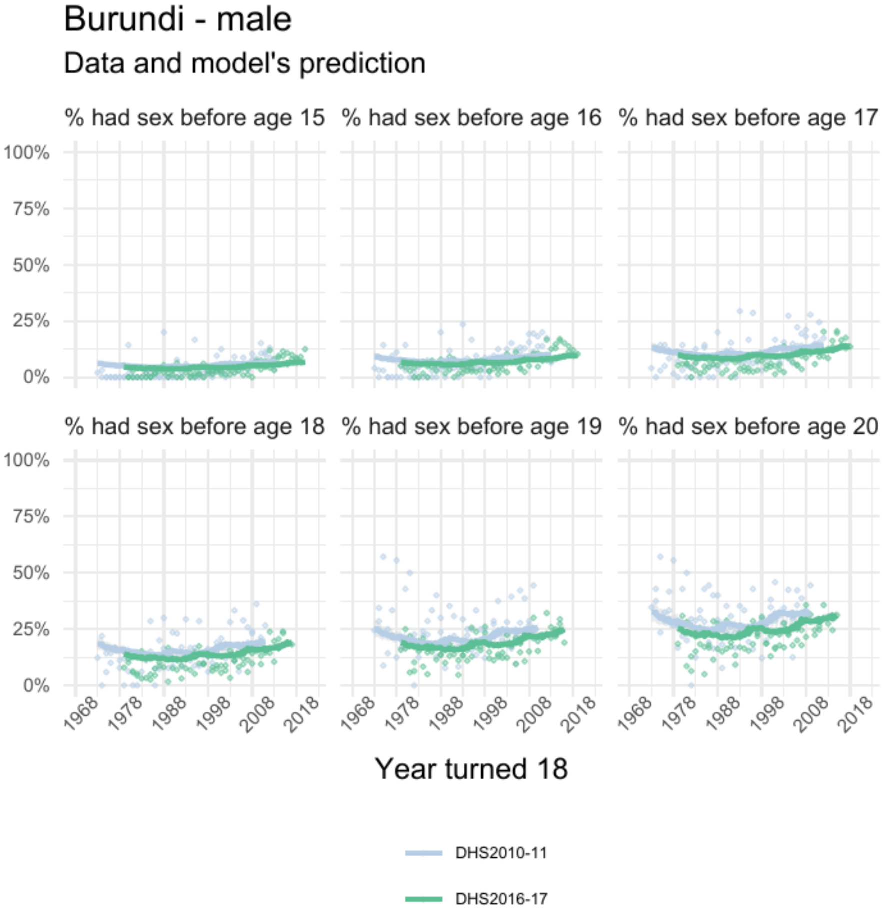

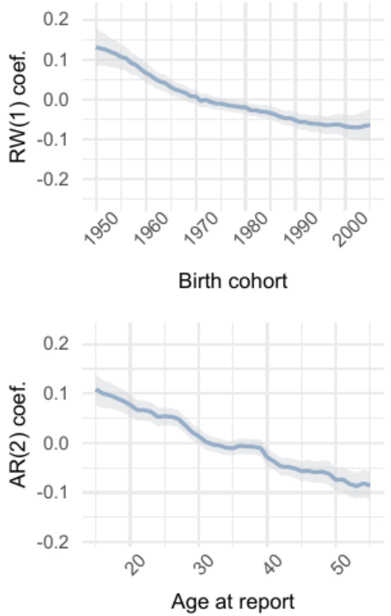

	Parameter	Estimate
1	intercept	-0.26 [-0.28 – -0.25]
2	skew	0.72 [0.68 – 0.76]
3	shape	8.84 [8.58 – 9.09]

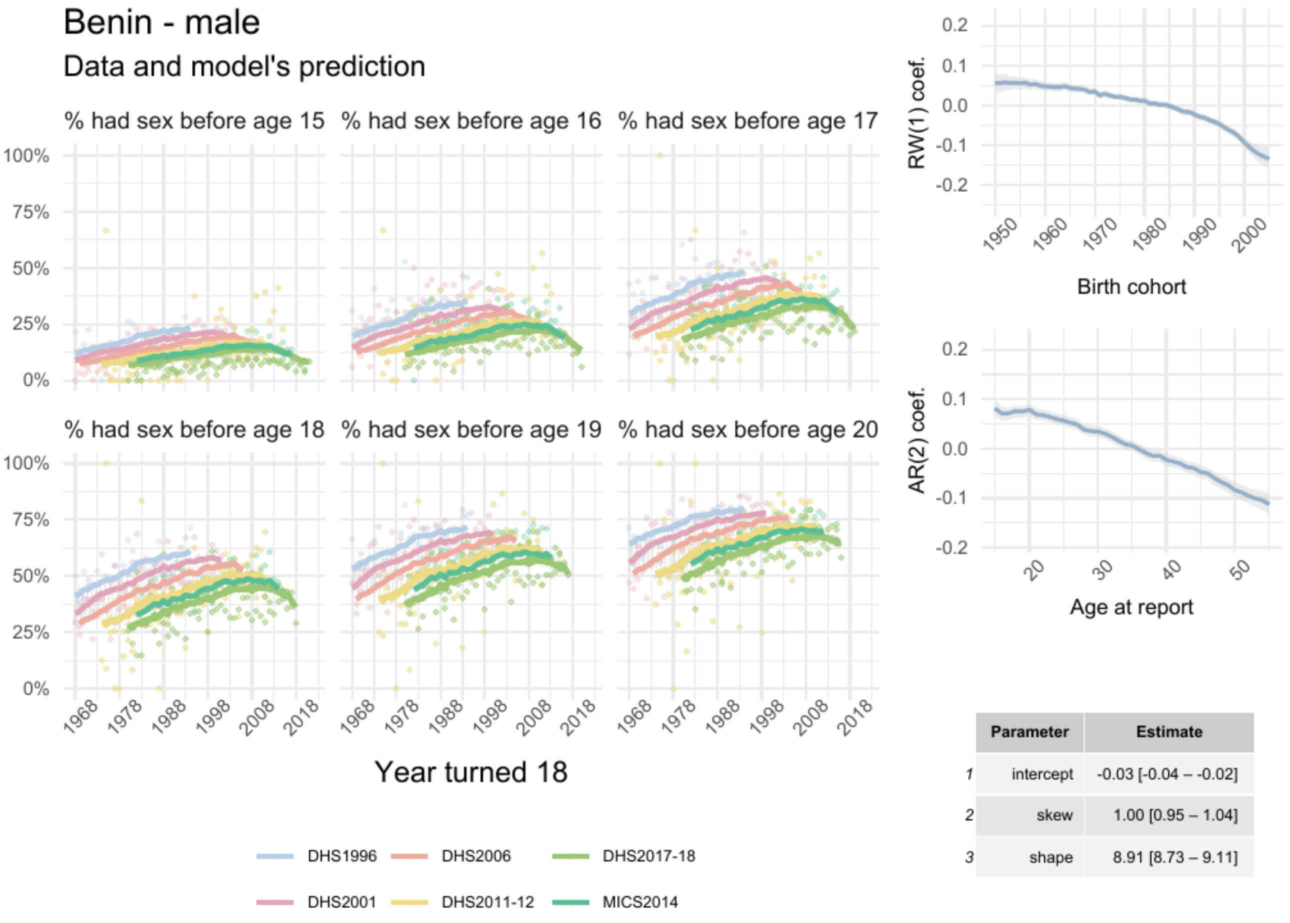

	Parameter	Estimate
1	intercept	-0.03 [-0.04 – -0.02]
2	skew	1.00 [0.95 – 1.04]
3	shape	8.91 [8.73 – 9.11]

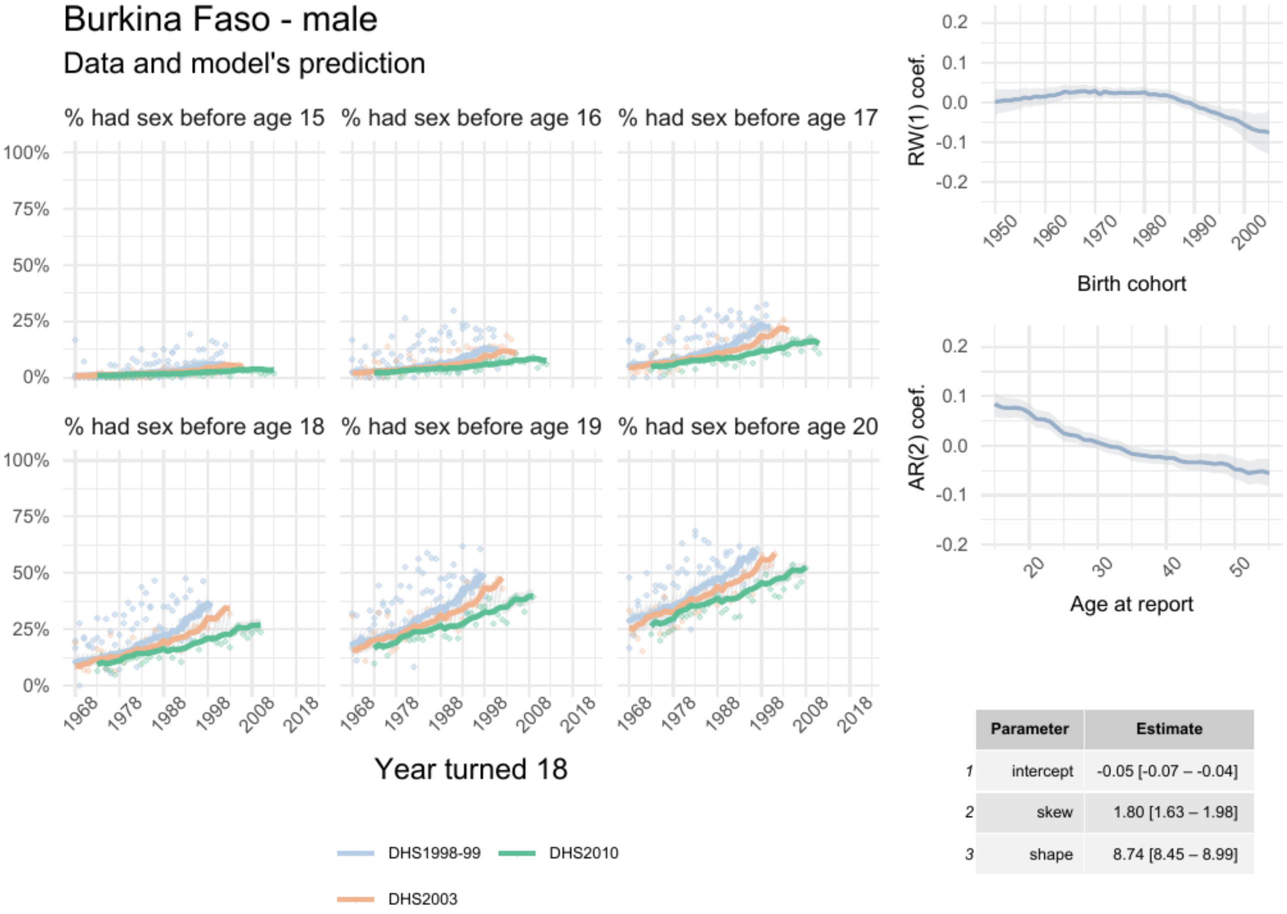

	Parameter	Estimate
1	intercept	-0.05 [-0.07 – -0.04]
2	skew	1.80 [1.63 – 1.98]
3	shape	8.74 [8.45 – 8.99]

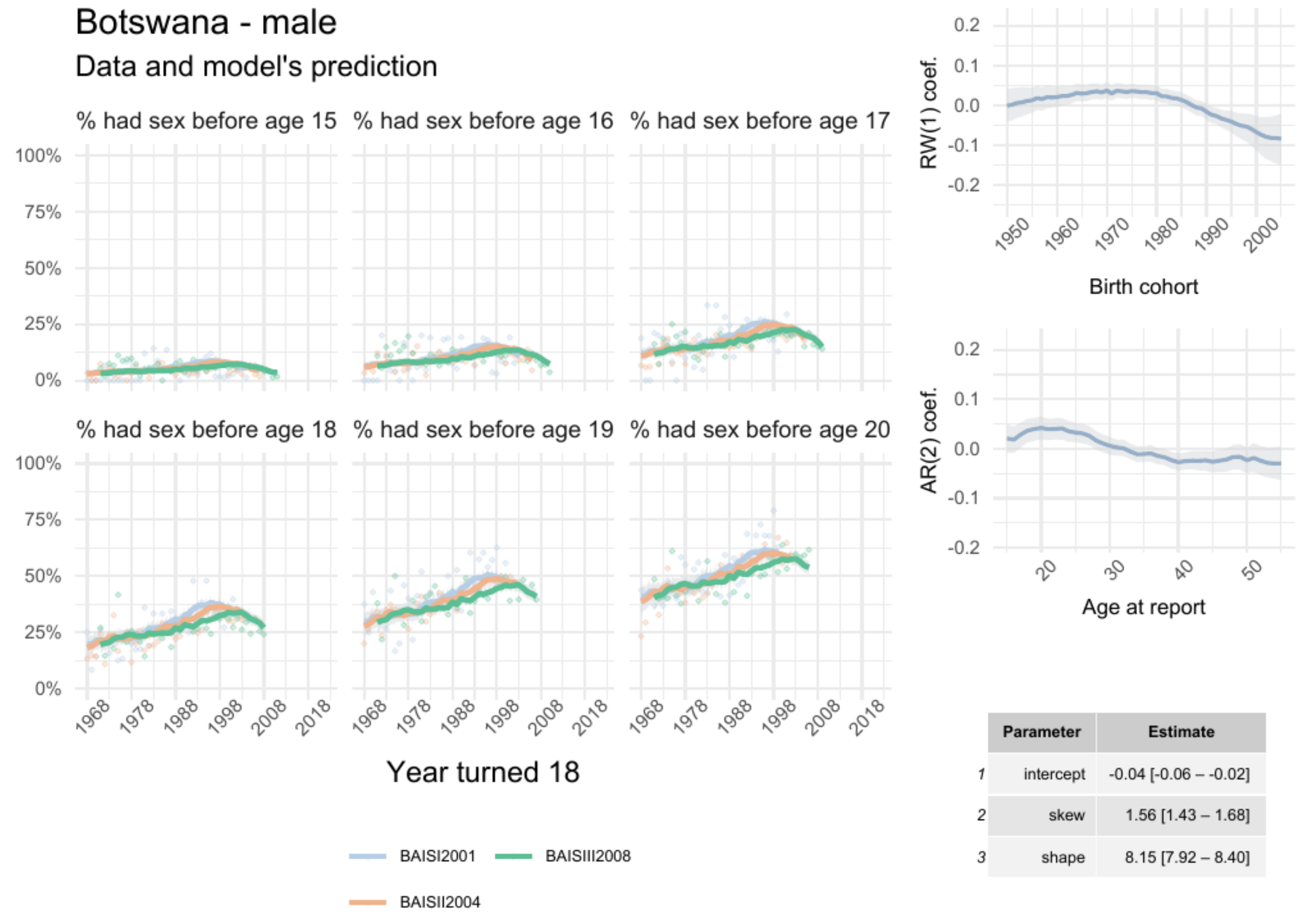

	Parameter	Estimate
1	intercept	-0.04 [-0.06 – -0.02]
2	skew	1.56 [1.43 – 1.68]
3	shape	8.15 [7.92 – 8.40]

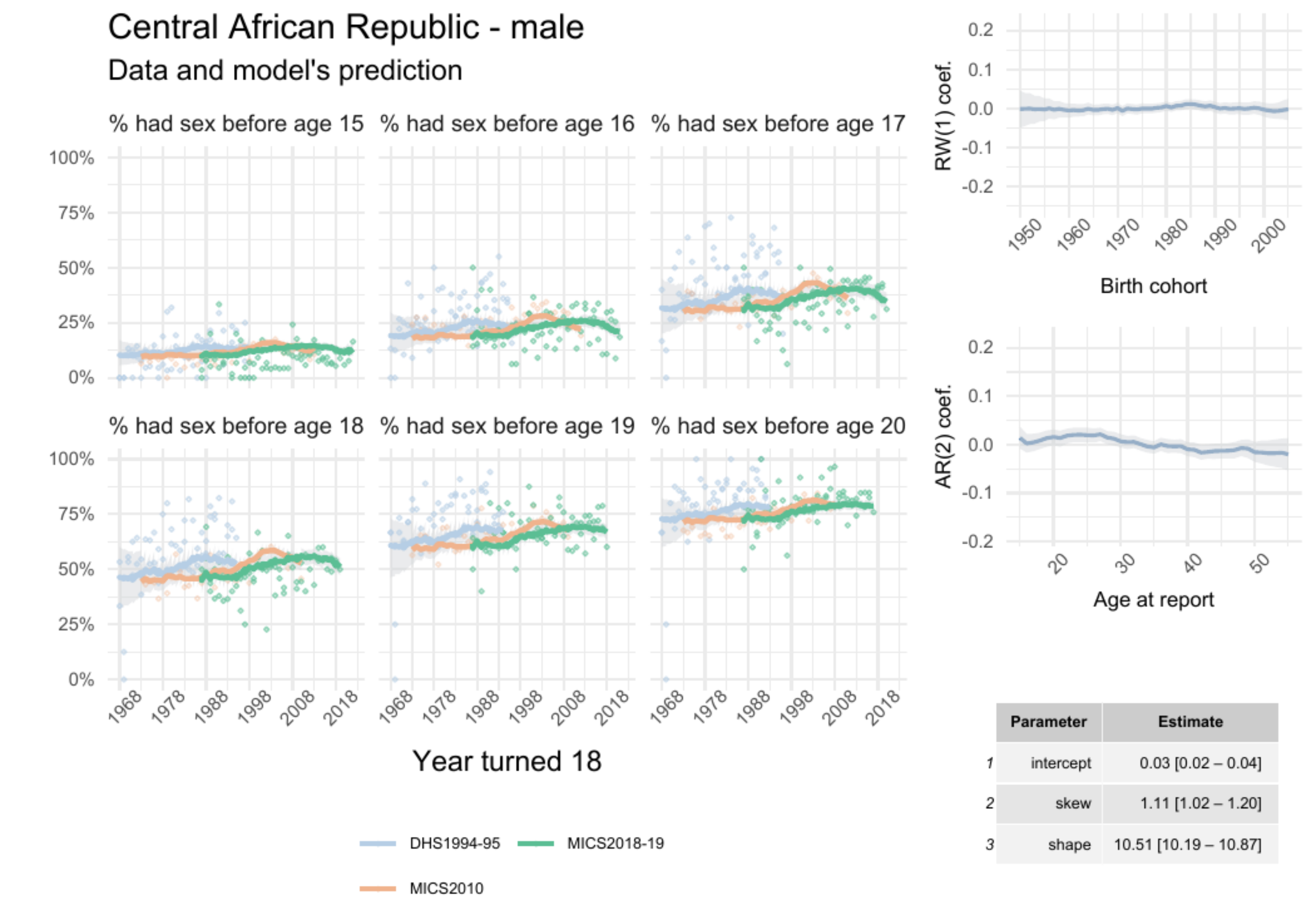

	Parameter	Estimate
1	intercept	0.03 [0.02 – 0.04]
2	skew	1.11 [1.02 – 1.20]
3	shape	10.51 [10.19 – 10.87]

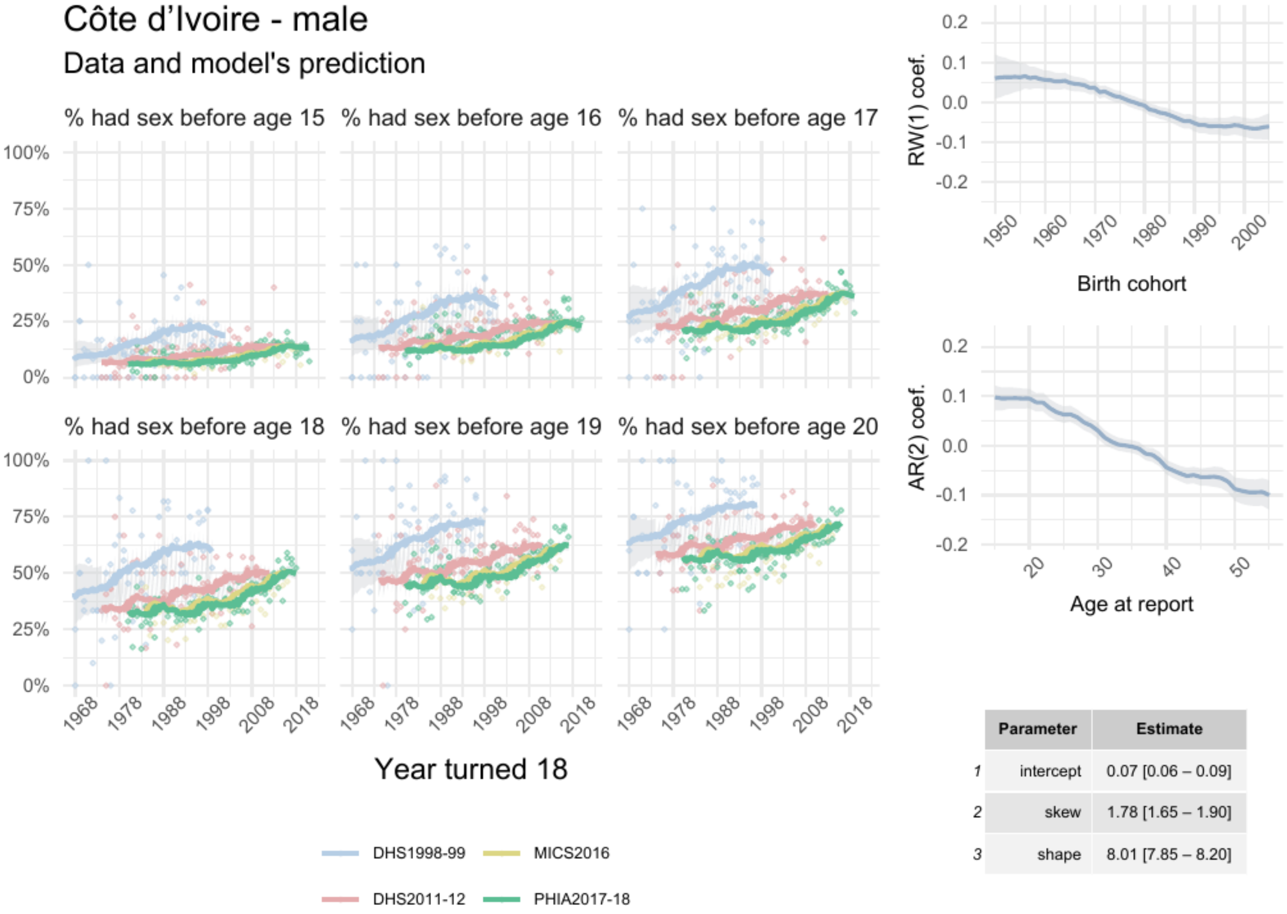

	Parameter	Estimate
1	intercept	0.07 [0.06 – 0.09]
2	skew	1.78 [1.65 – 1.90]
3	shape	8.01 [7.85 – 8.20]

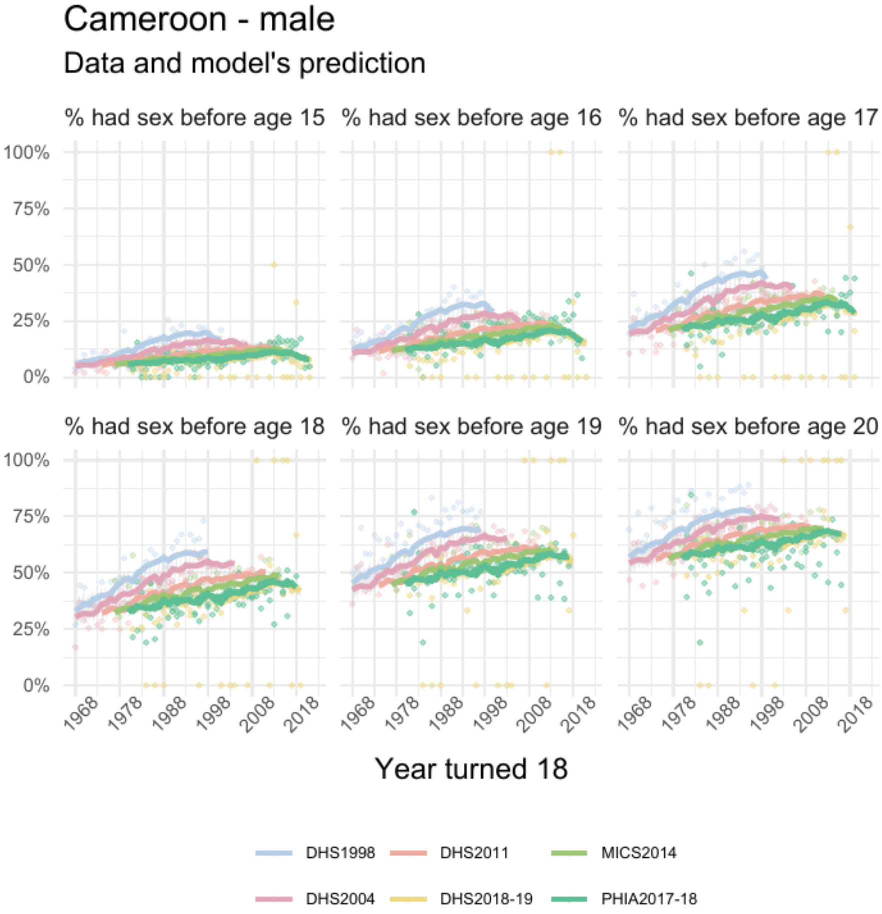

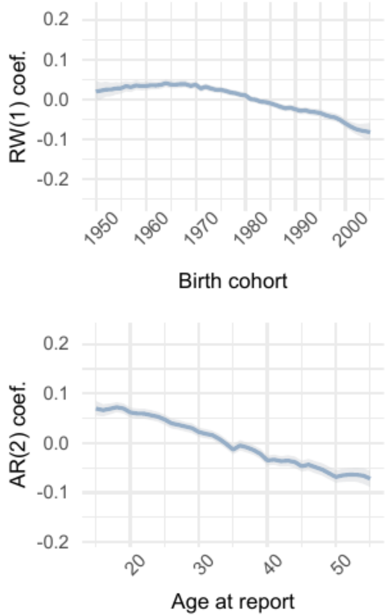

	Parameter	Estimate
1	intercept	0.10 [0.09 – 0.11]
2	skew	2.13 [2.01 – 2.27]
3	shape	7.72 [7.60 – 7.85]

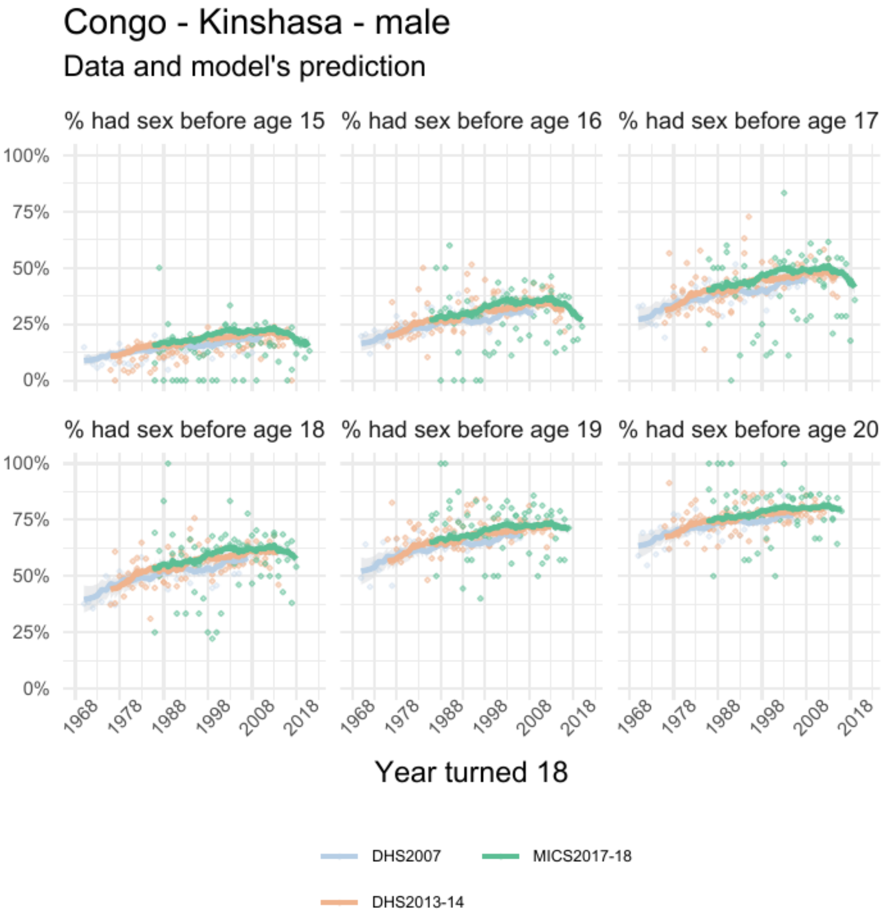

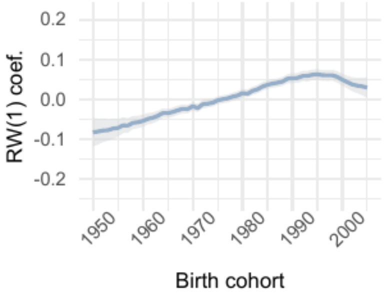

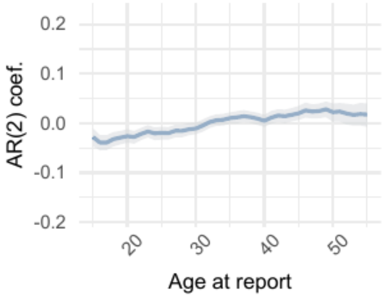

	Parameter	Estimate
1	intercept	0.11 [0.09 – 0.12]
2	skew	1.62 [1.53 – 1.73]
3	shape	8.24 [8.06 – 8.42]

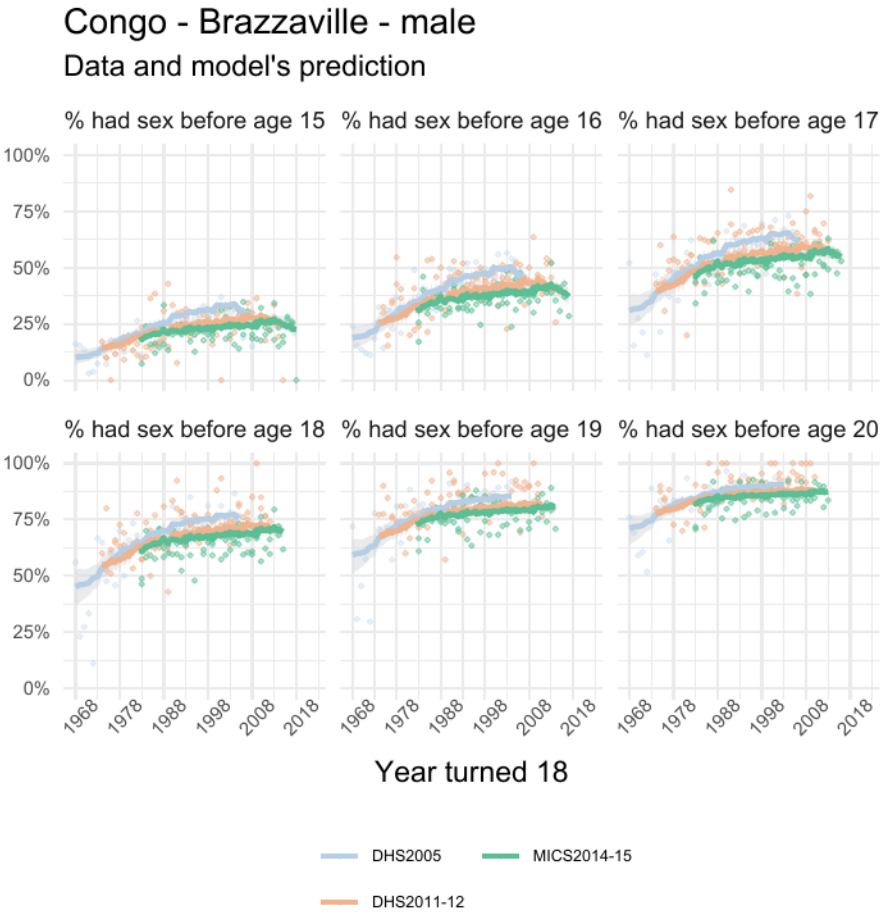

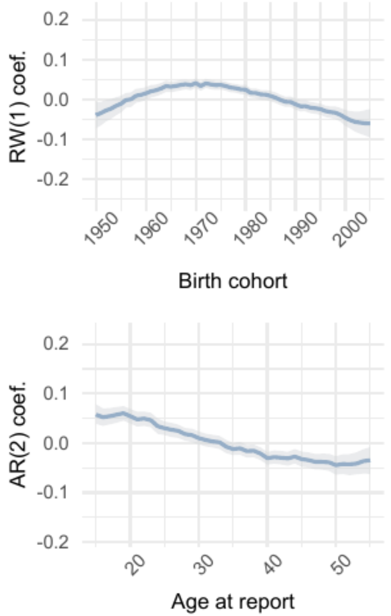

	Parameter	Estimate
1	intercept	0.09 [0.08 – 0.11]
2	skew	1.21 [1.13 – 1.29]
3	shape	9.93 [9.68 – 10.21]

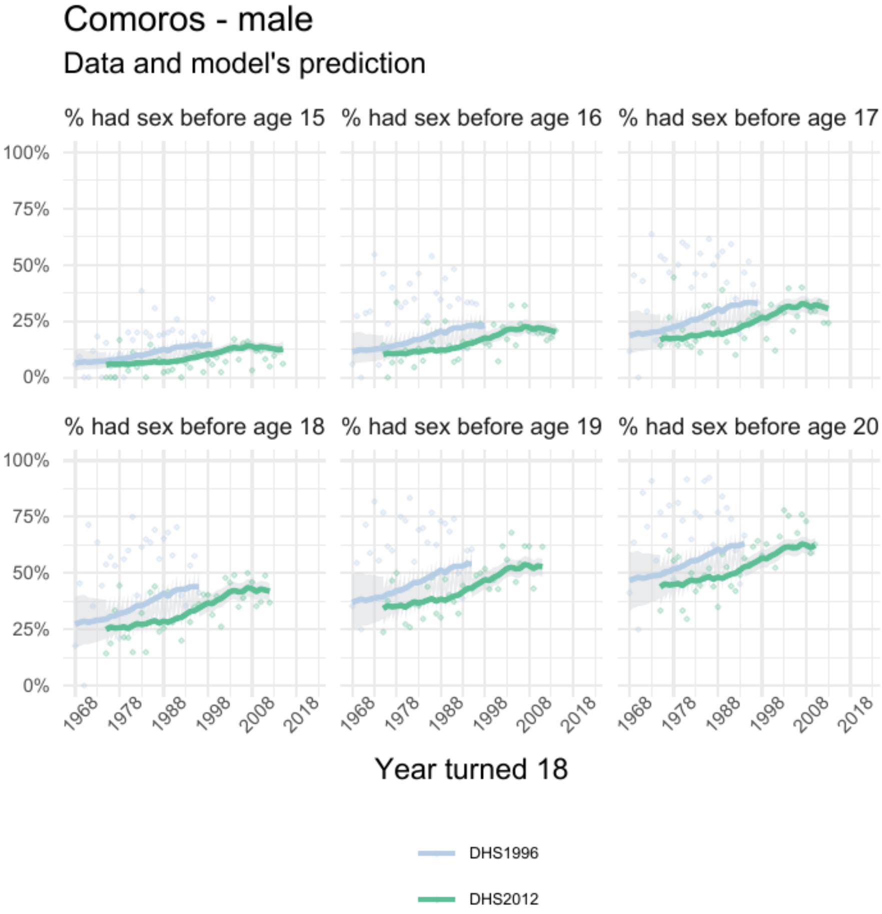

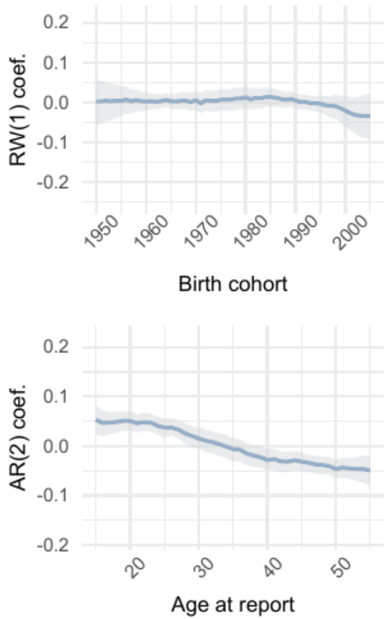

	Parameter	Estimate
1	intercept	0.06 [0.02 – 0.10]
2	skew	1.96 [1.64 – 2.40]
3	shape	6.48 [6.09 – 6.86]

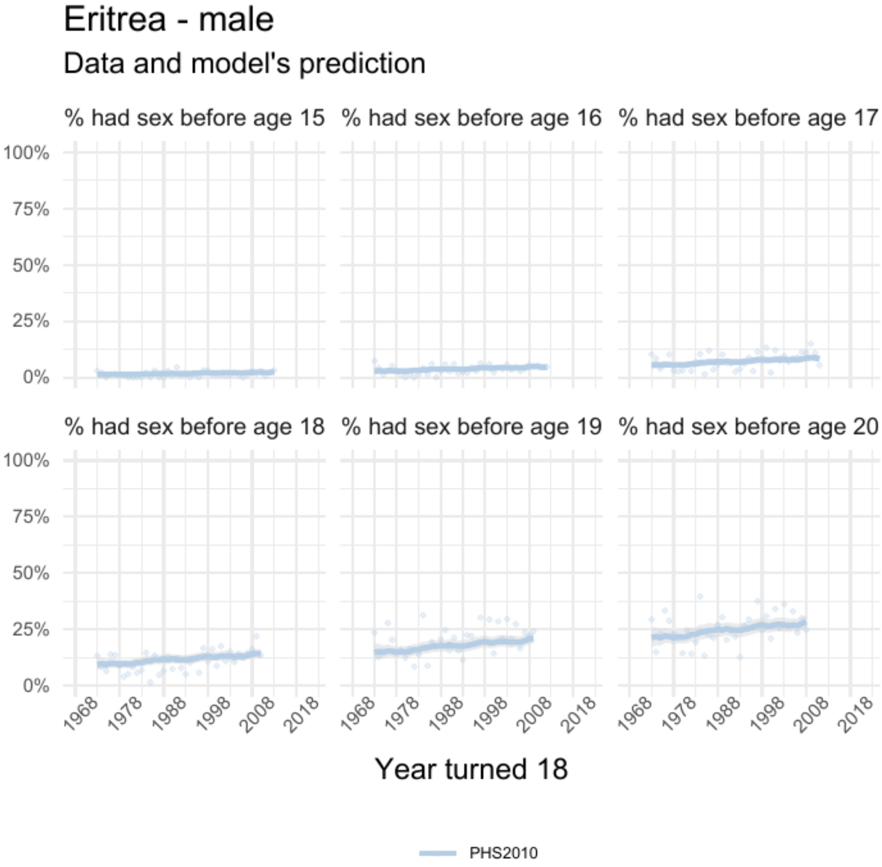

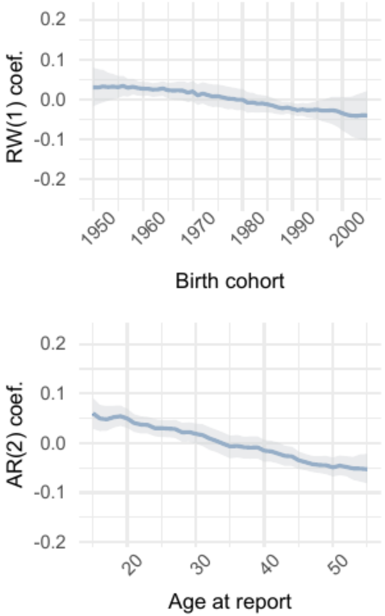

	Parameter	Estimate
1	intercept	0.02 [-0.04 – 0.07]
2	skew	3.20 [2.57 – 3.93]
3	shape	5.36 [5.12 – 5.64]

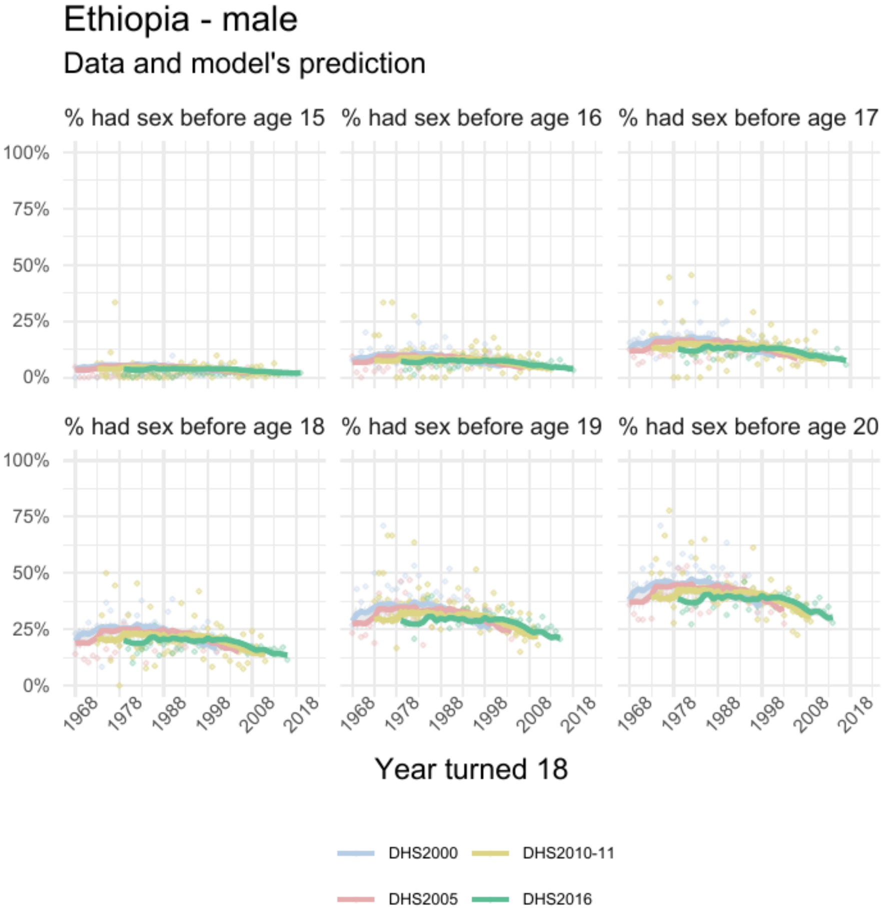

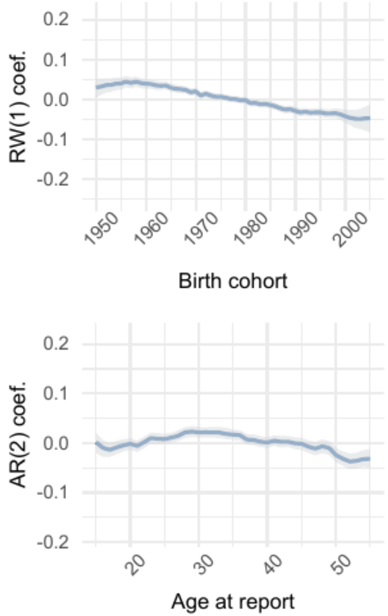

	Parameter	Estimate
1	intercept	-0.020 [-0.035 – -0.007]
2	skew	2.04 [1.91 – 2.16]
3	shape	6.65 [6.55 – 6.76]

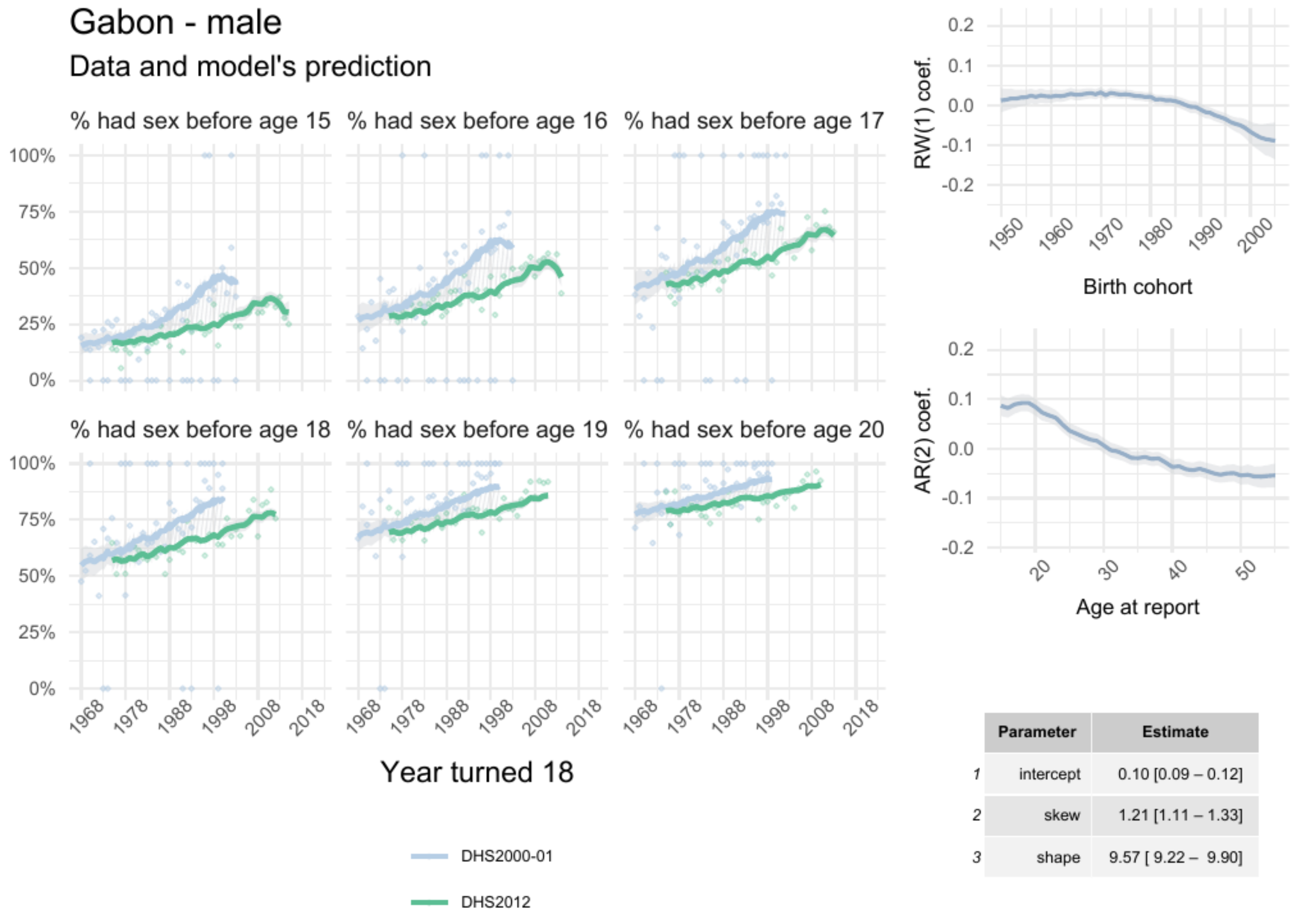

	Parameter	Estimate
1	intercept	0.10 [0.09 – 0.12]
2	skew	1.21 [1.11 – 1.33]
3	shape	9.57 [9.22 – 9.90]

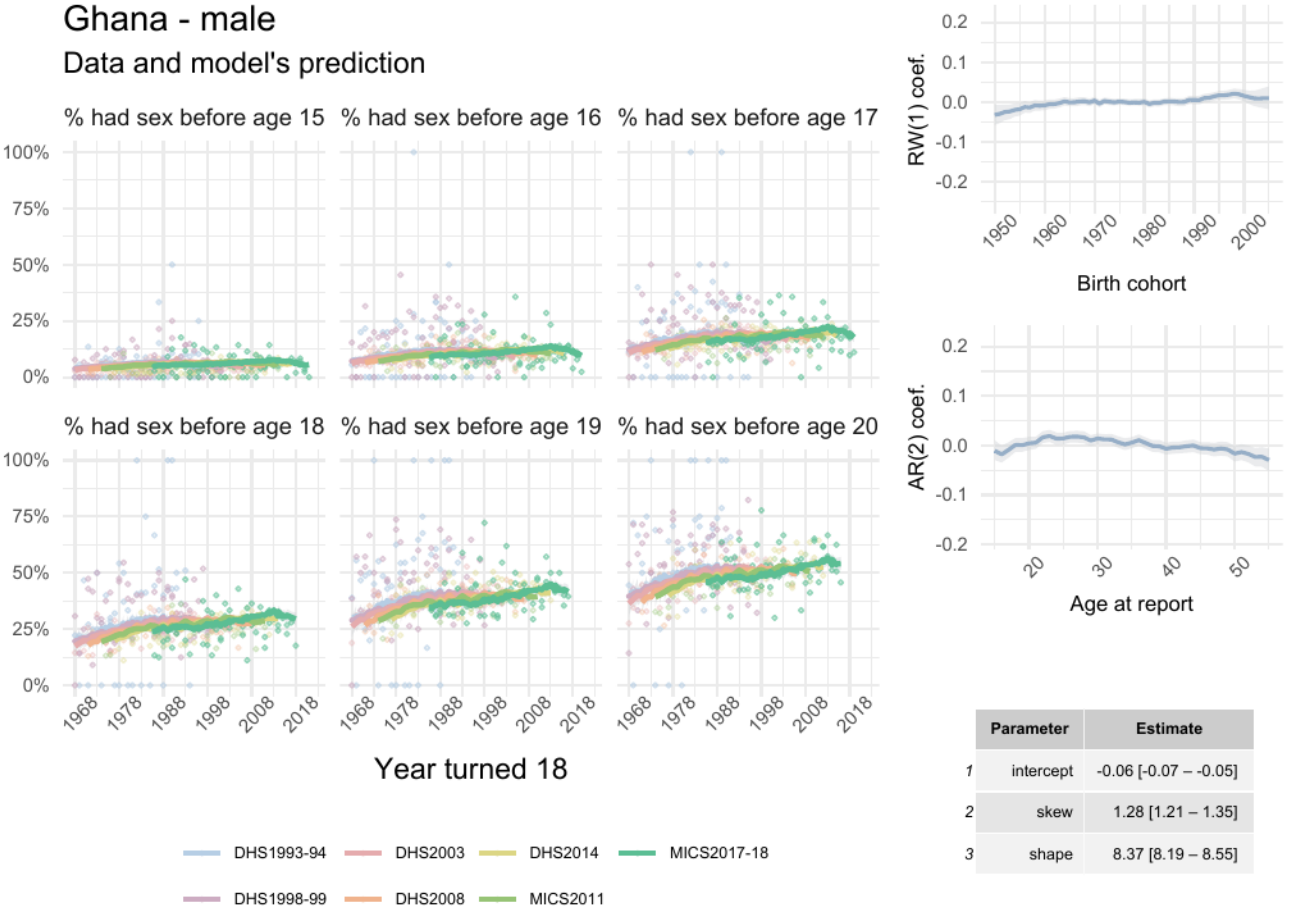

	Parameter	Estimate
1	intercept	-0.06 [-0.07 – -0.05]
2	skew	1.28 [1.21 – 1.35]
3	shape	8.37 [8.19 – 8.55]

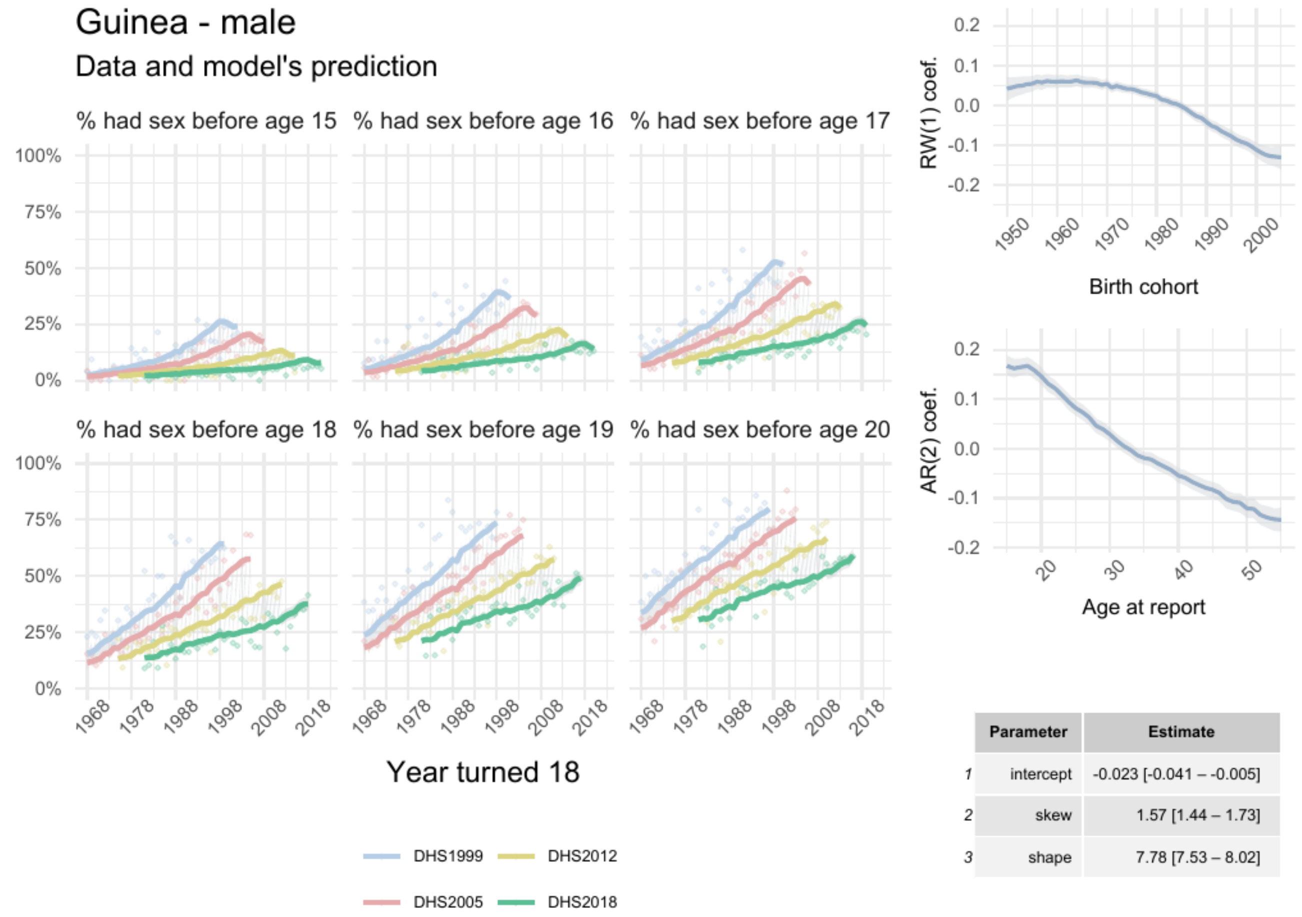

	Parameter	Estimate
1	intercept	-0.023 [-0.041 – -0.005]
2	skew	1.57 [1.44 – 1.73]
3	shape	7.78 [7.53 – 8.02]

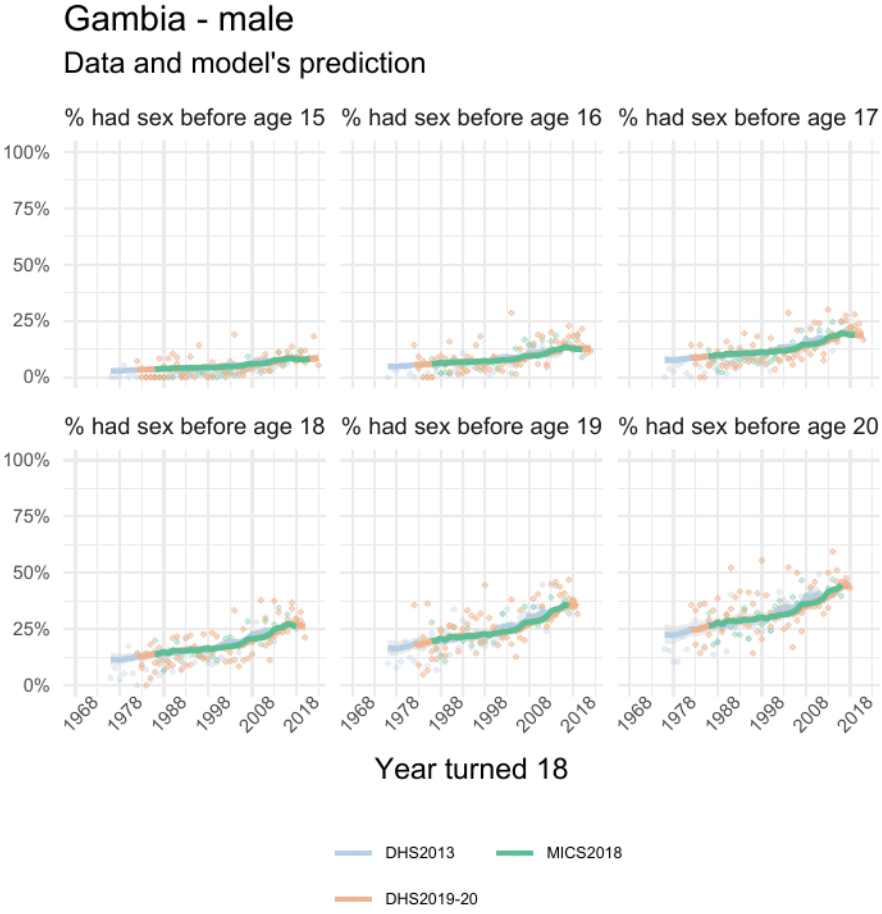

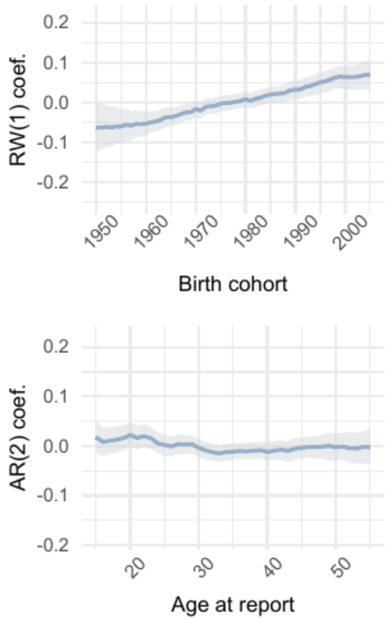

	Parameter	Estimate
1	intercept	-0.14 [-0.17 – -0.12]
2	skew	1.34 [1.23 – 1.46]
3	shape	6.45 [6.23 – 6.67]

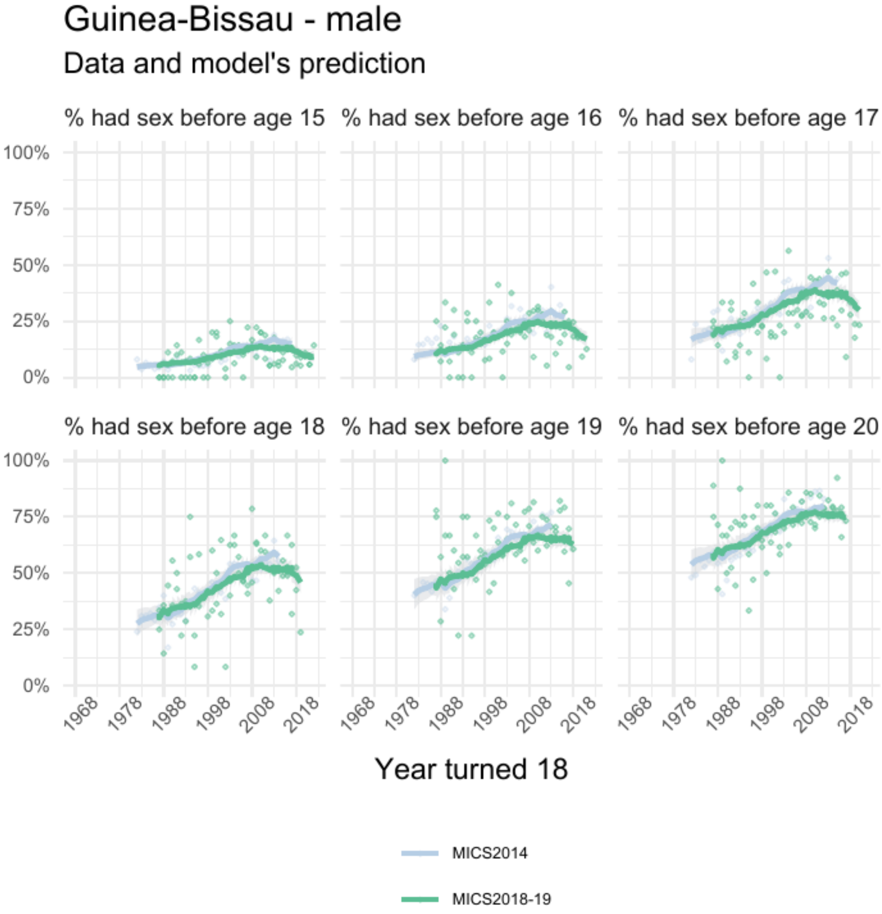

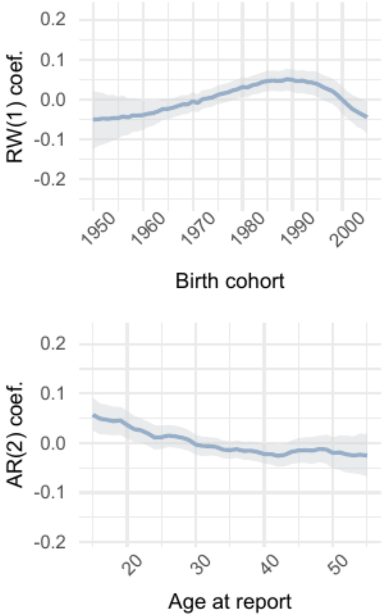

	Parameter	Estimate
1	intercept	-0.003 [-0.025 – 0.020]
2	skew	1.24 [1.12 – 1.38]
3	shape	9.80 [9.41 – 10.22]

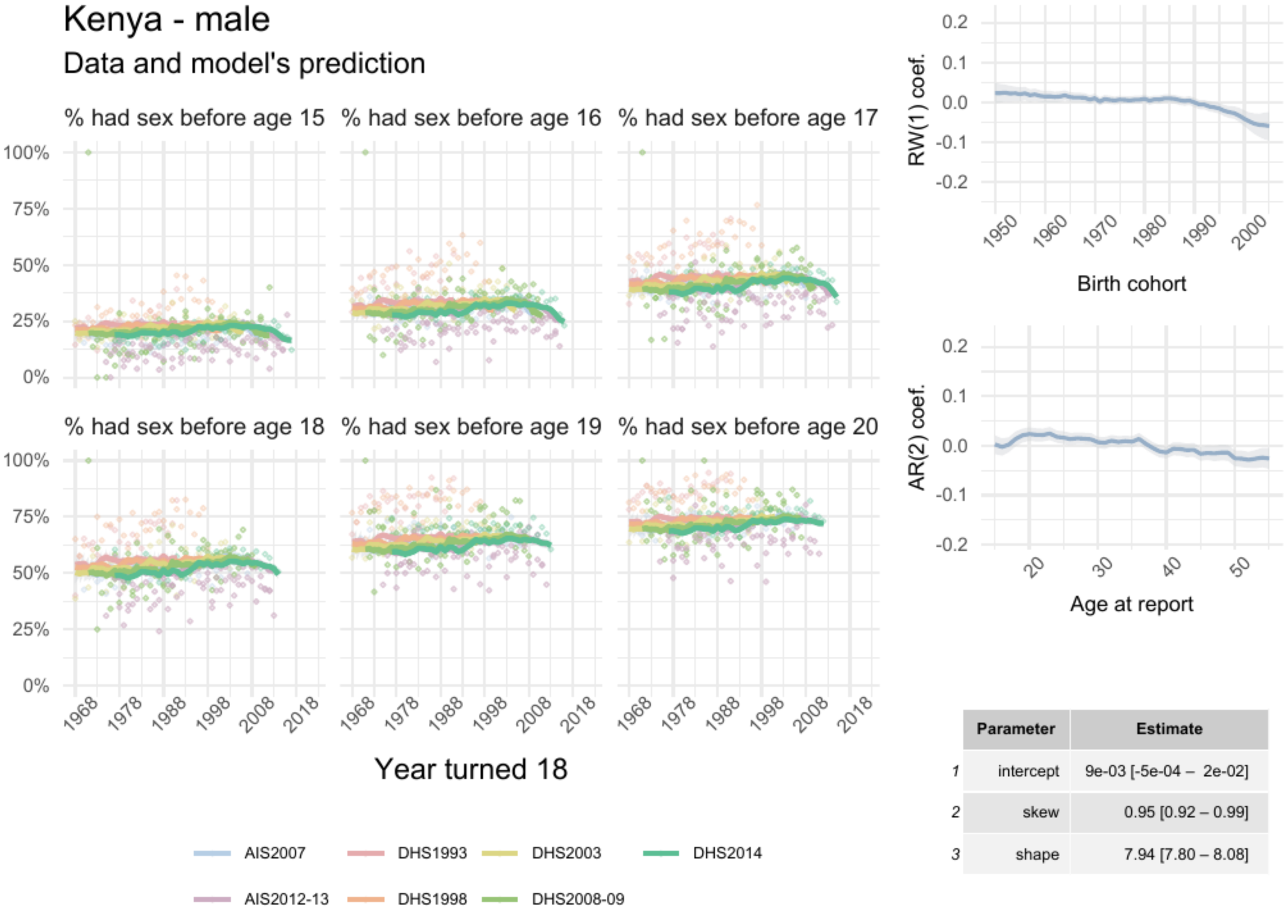

	Parameter	Estimate
1	intercept	9e-03 [-5e-04 – 2e-02]
2	skew	0.95 [0.92 – 0.99]
3	shape	7.94 [7.80 – 8.08]

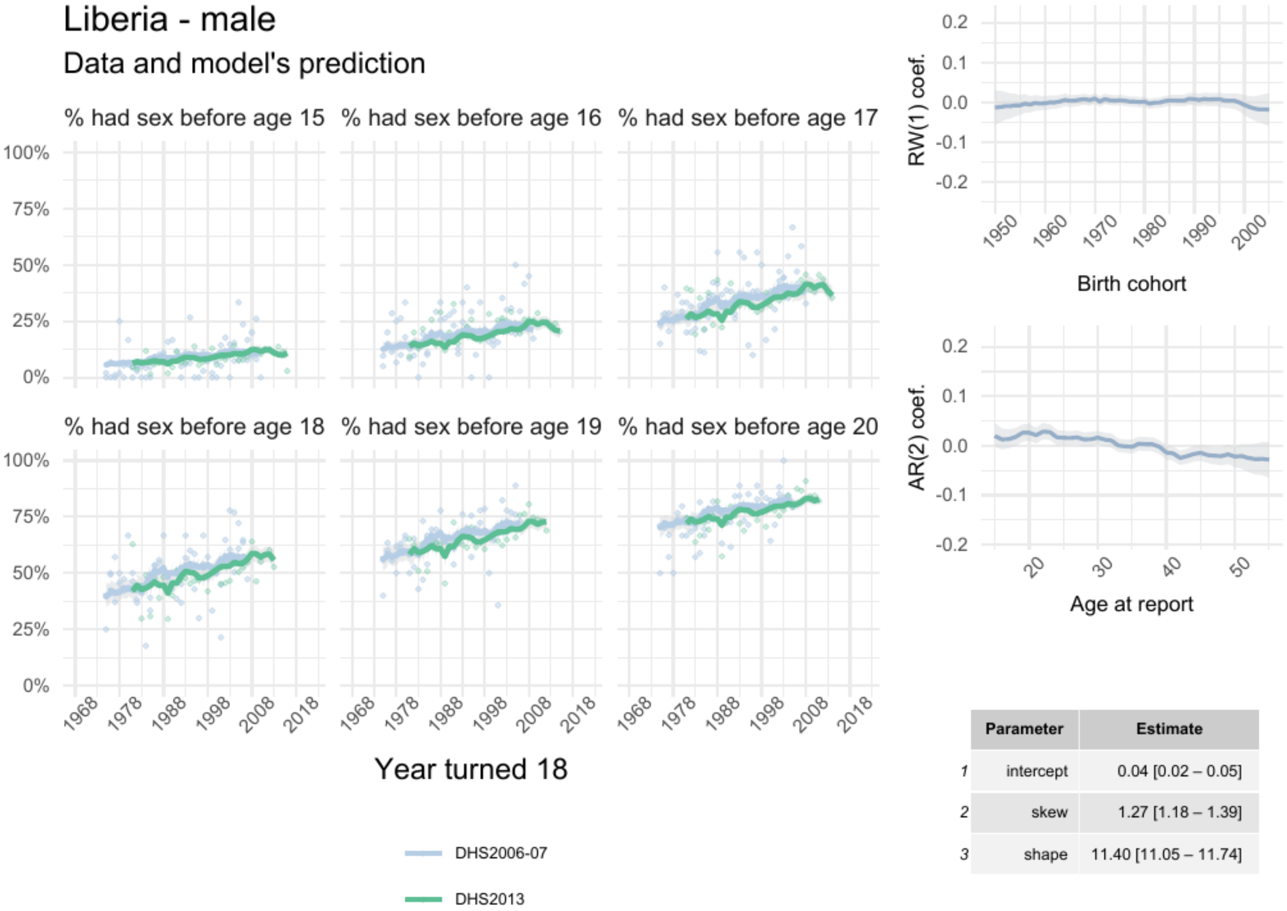

	Parameter	Estimate
1	intercept	0.04 [0.02 – 0.05]
2	skew	1.27 [1.18 – 1.39]
3	shape	11.40 [11.05 – 11.74]

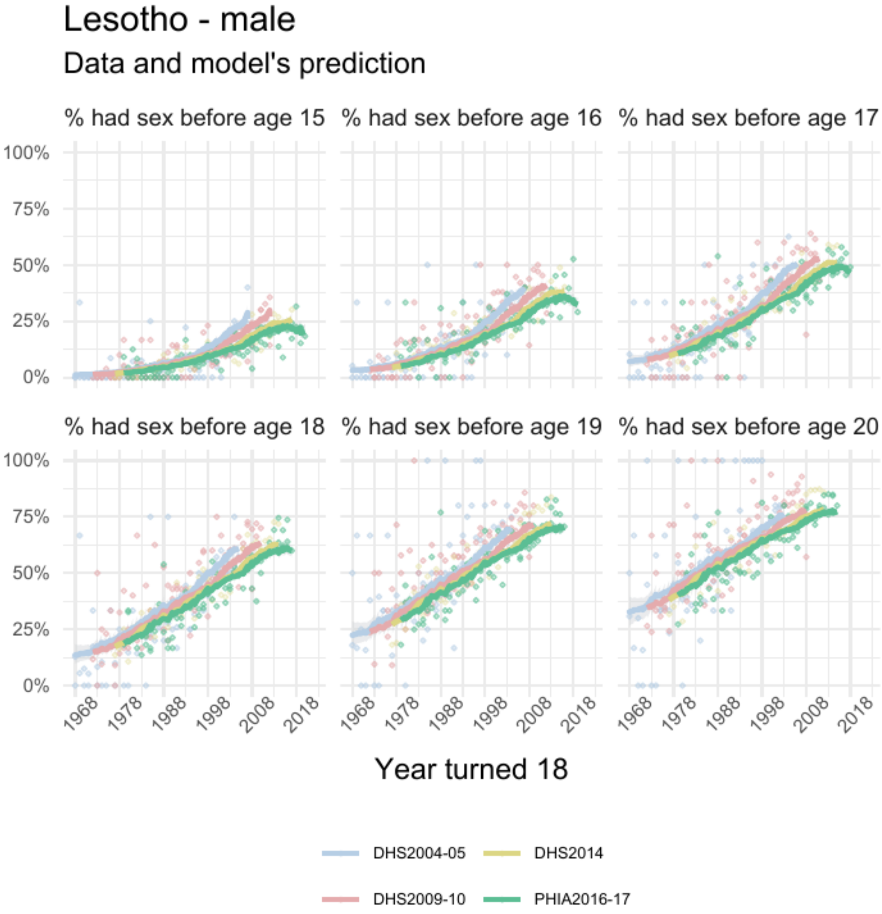

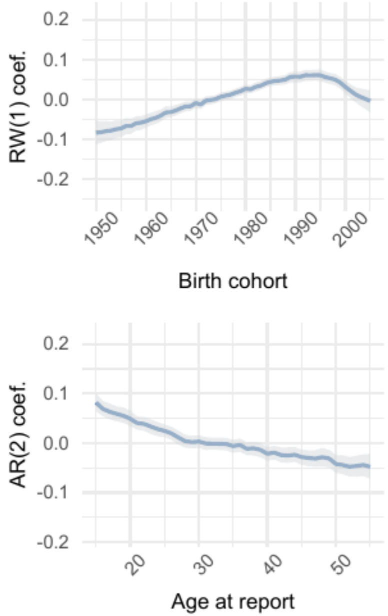

	Parameter	Estimate
1	intercept	0.16 [0.14 – 0.18]
2	skew	3.11 [2.81 – 3.43]
3	shape	6.96 [6.80 – 7.11]

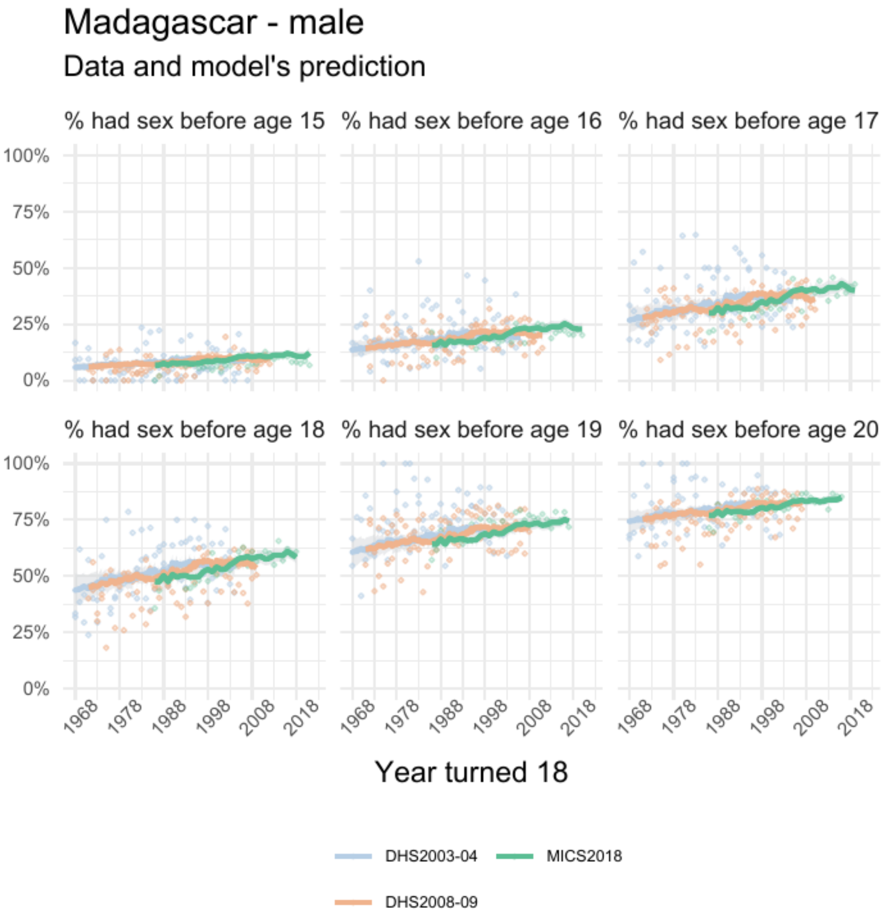

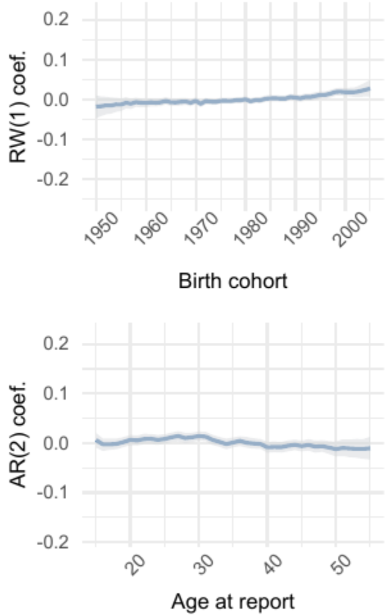

	Parameter	Estimate
1	intercept	0.06 [0.05 – 0.07]
2	skew	1.38 [1.29 – 1.45]
3	shape	11.68 [11.40 – 11.96]

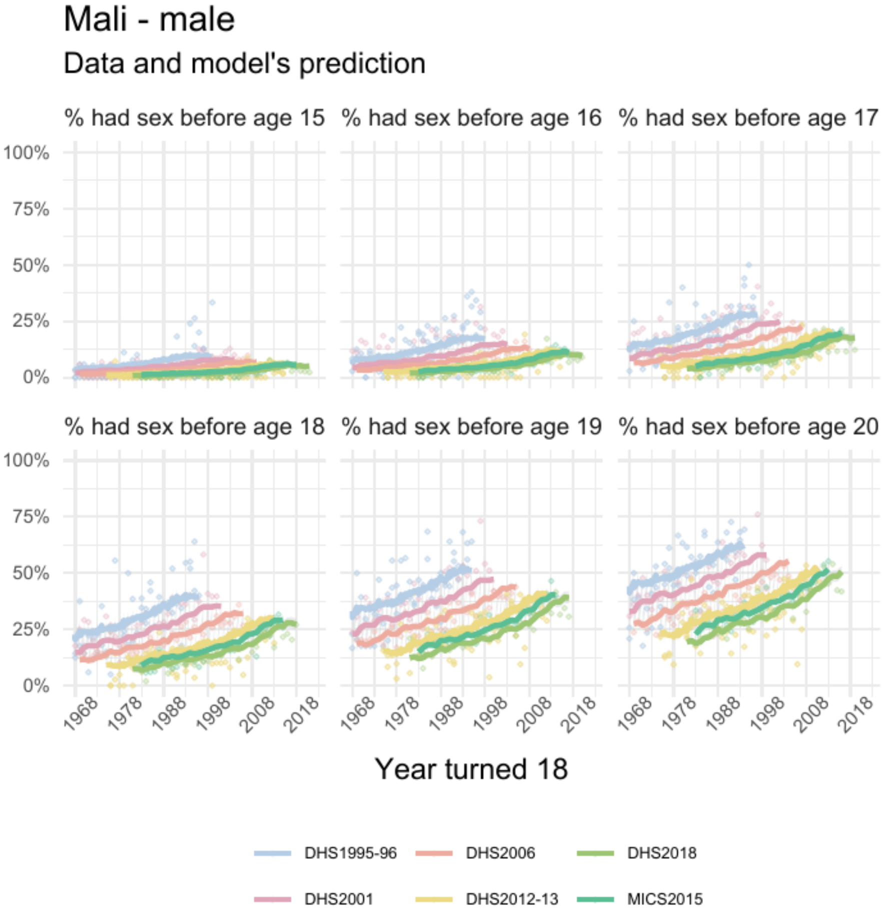

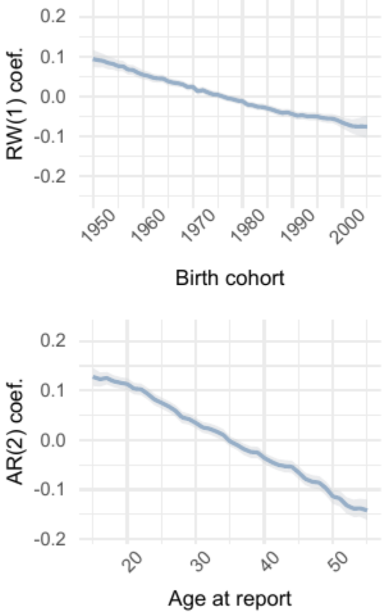

	Parameter	Estimate
1	intercept	-0.06 [-0.07 – -0.04]
2	skew	1.70 [1.60 – 1.82]
3	shape	7.74 [7.58 – 7.91]

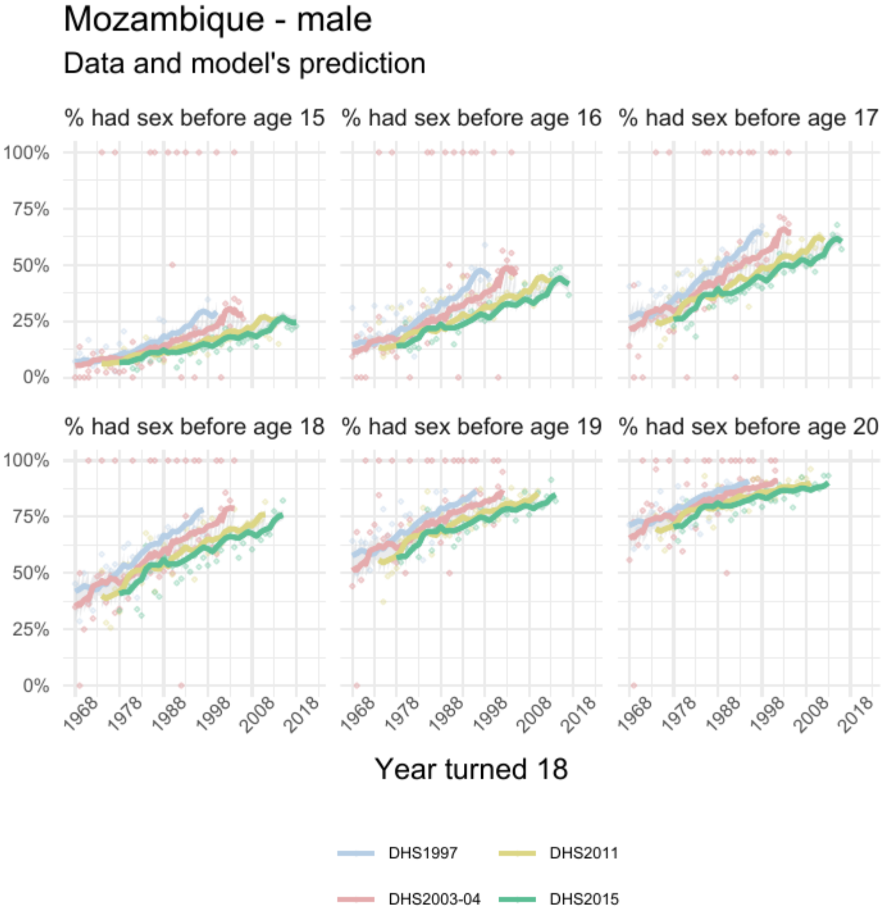

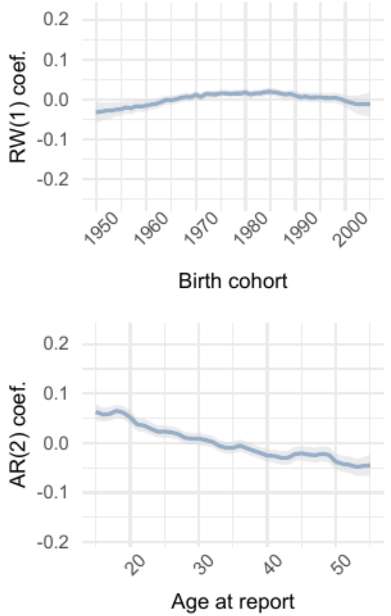

	Parameter	Estimate
1	intercept	0.07 [0.06 – 0.08]
2	skew	1.21 [1.12 – 1.30]
3	shape	11.22 [10.89 – 11.52]

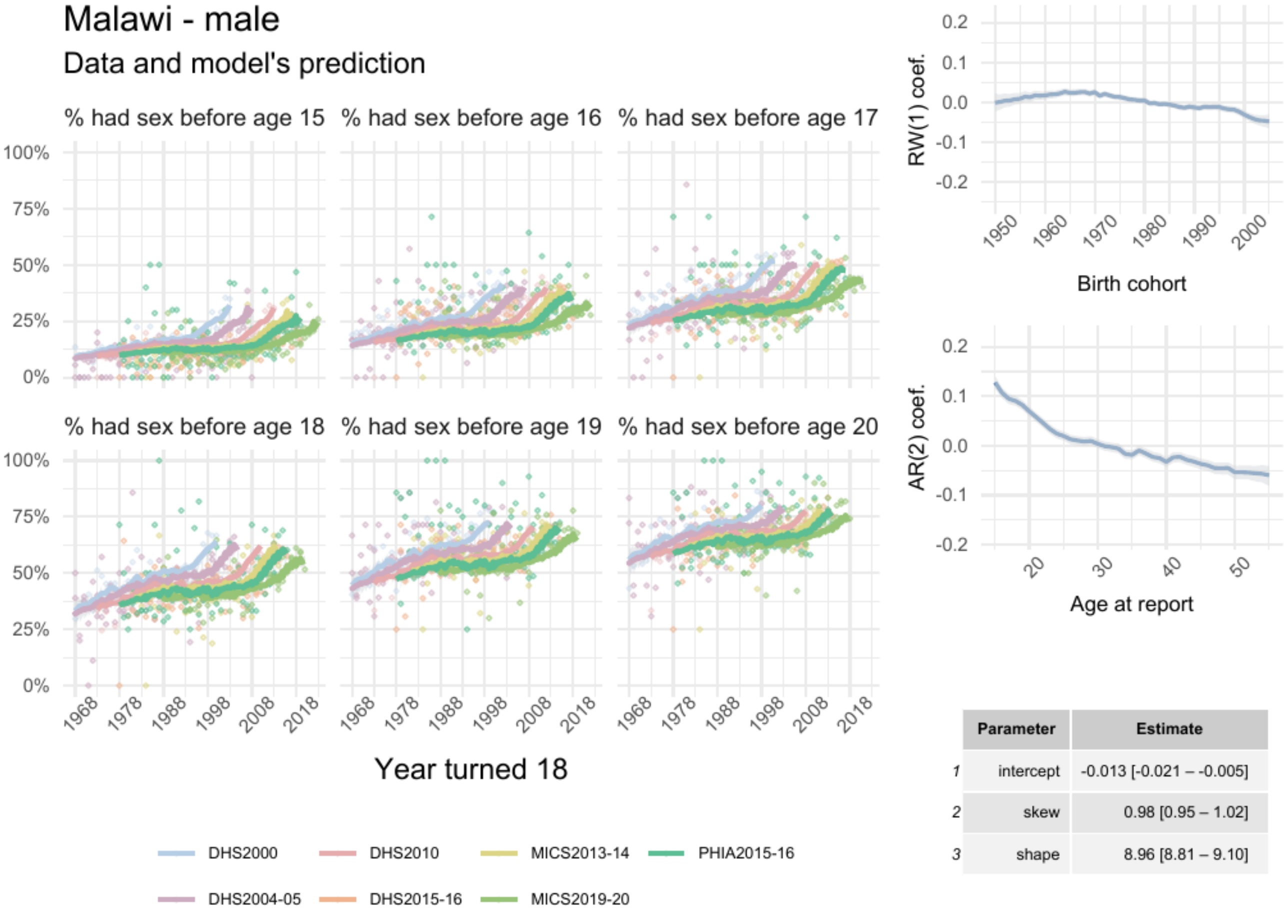

	Parameter	Estimate
1	intercept	-0.013 [-0.021 – -0.005]
2	skew	0.98 [0.95 – 1.02]
3	shape	8.96 [8.81 – 9.10]

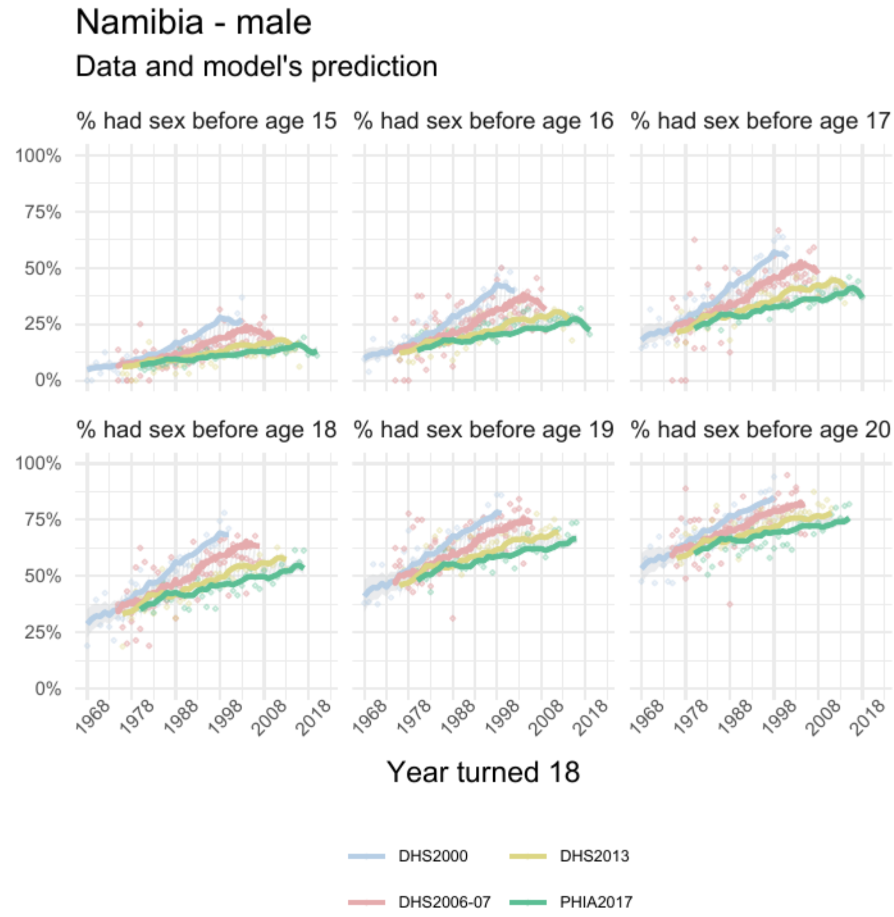

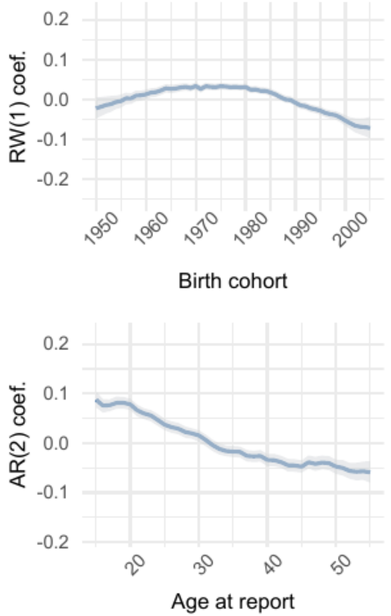

	Parameter	Estimate
1	intercept	0.07 [0.06 – 0.09]
2	skew	1.68 [1.58 – 1.79]
3	shape	8.39 [8.20 – 8.57]

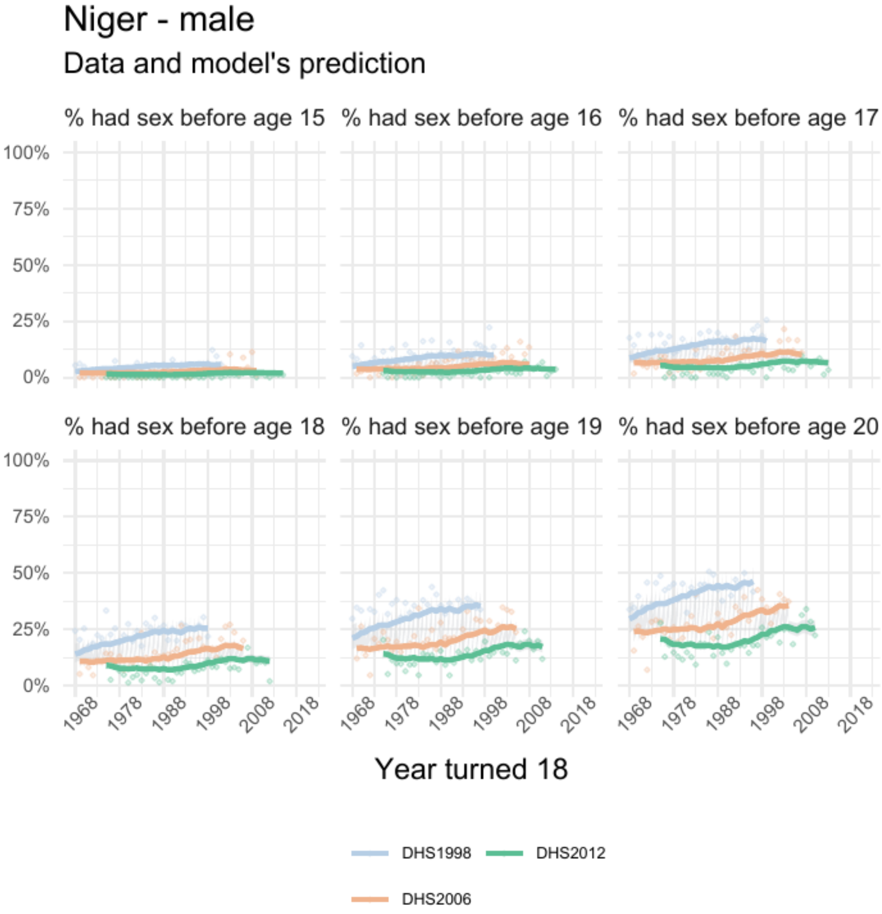

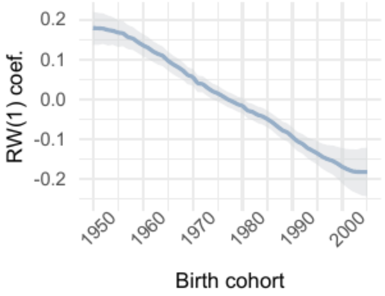

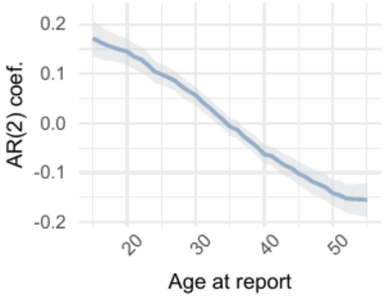

	Parameter	Estimate
1	intercept	-0.18 [-0.21 – -0.16]
2	skew	1.43 [1.28 – 1.61]
3	shape	7.49 [7.18 – 7.82]

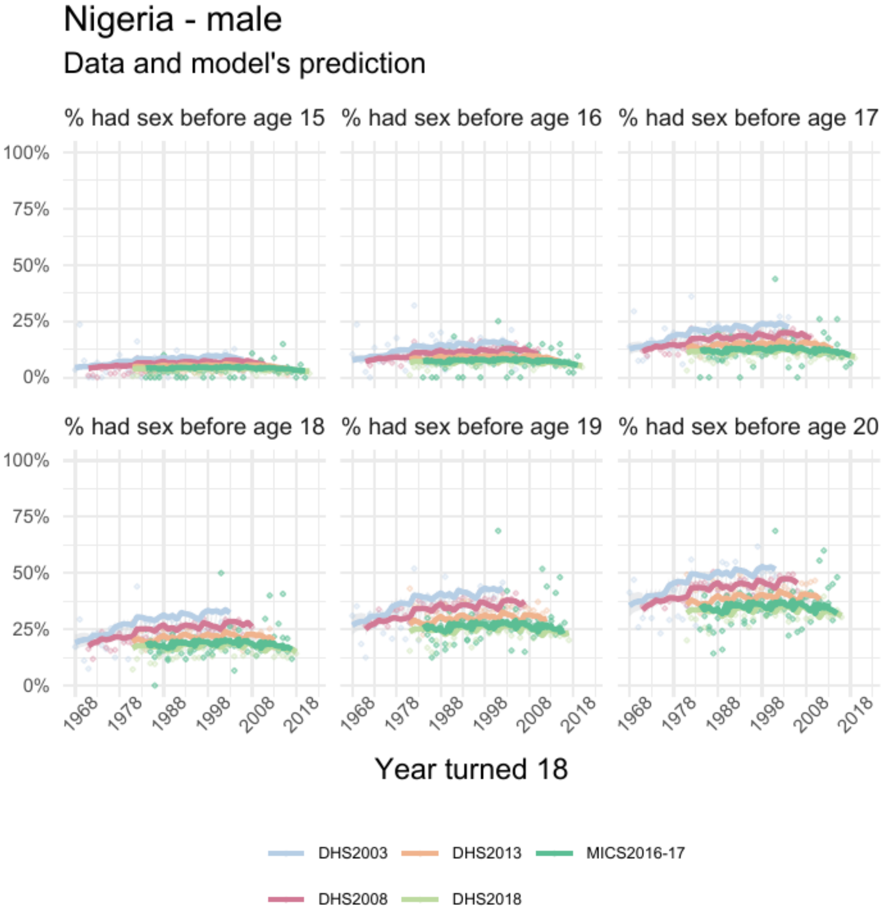

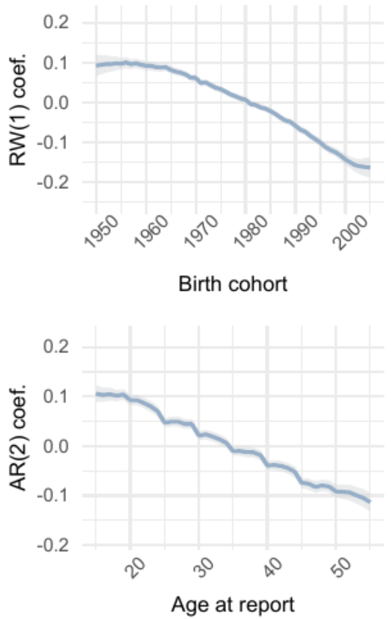

	Parameter	Estimate
1	intercept	-0.07 [-0.08 – -0.06]
2	skew	1.57 [1.52 – 1.63]
3	shape	6.66 [6.56 – 6.74]

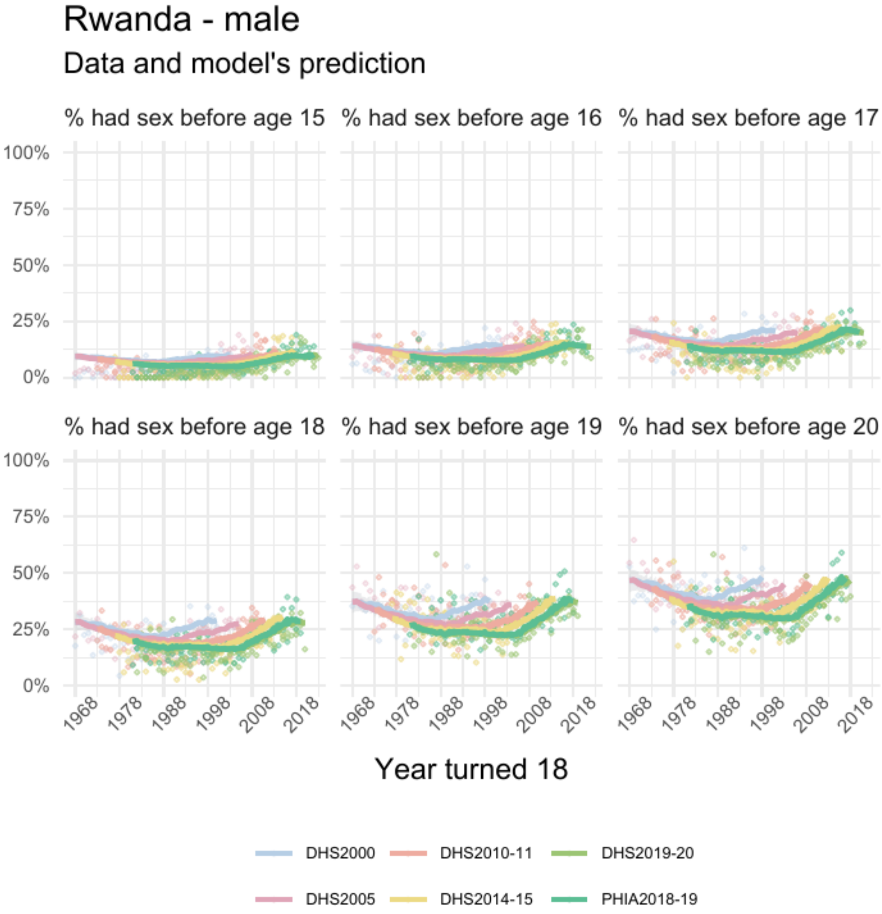

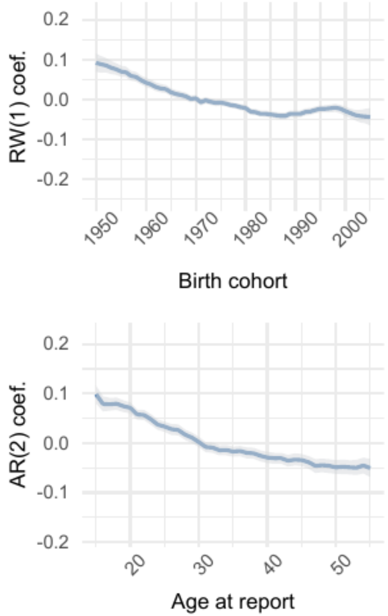

	Parameter	Estimate
1	intercept	-0.18 [-0.19 – -0.17]
2	skew	0.85 [0.82 – 0.89]
3	shape	8.17 [8.01 – 8.34]

Sudan - male

Data and model's prediction

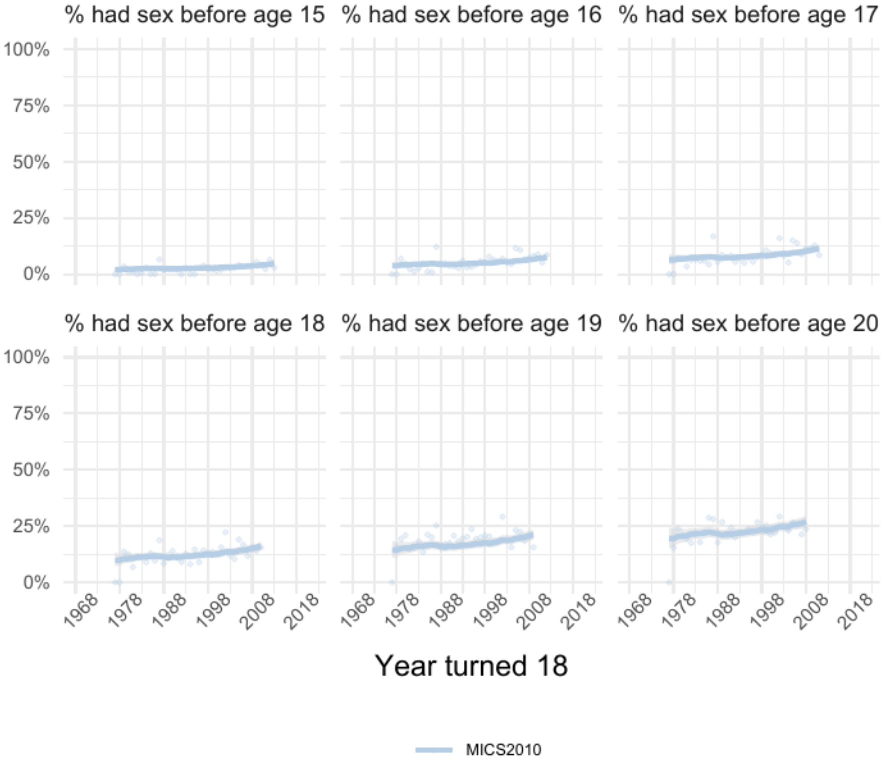

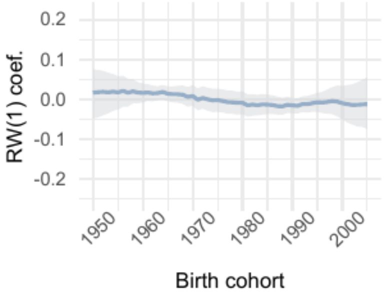

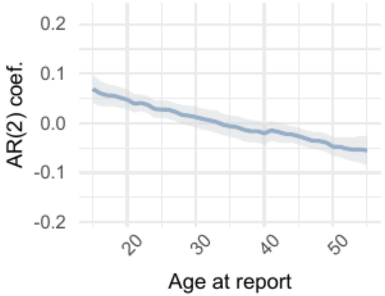

	Parameter	Estimate
1	intercept	0.03 [-0.04 – 0.10]
2	skew	3.17 [2.56 – 3.93]
3	shape	4.42 [4.22 – 4.66]

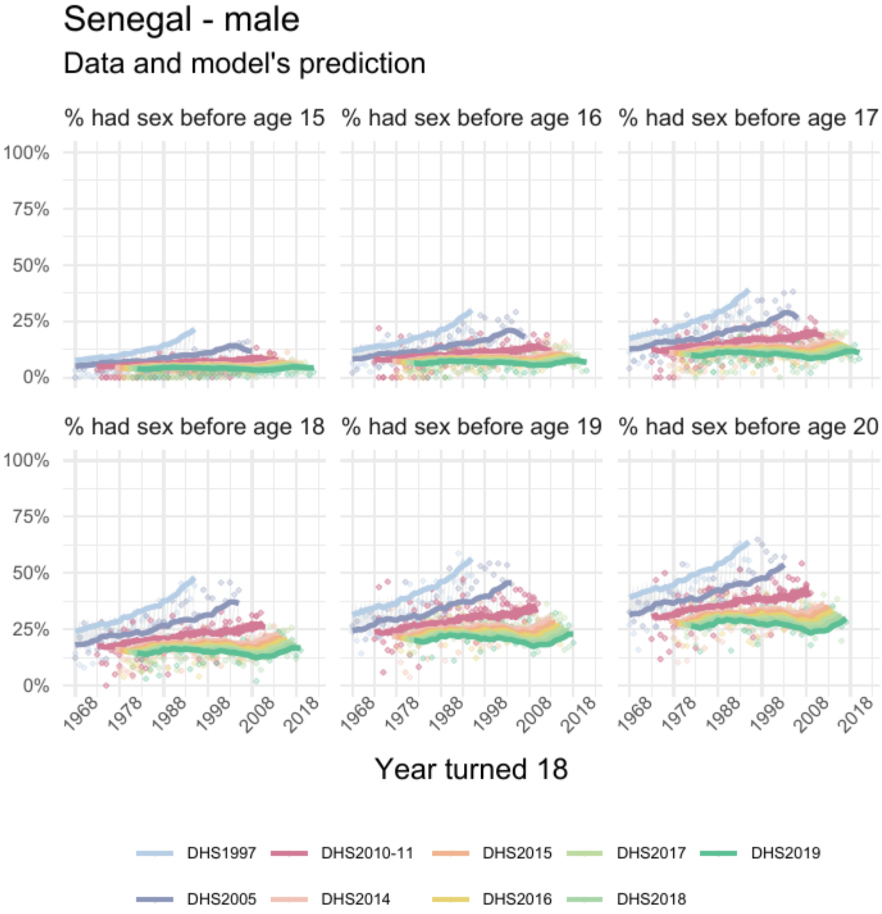

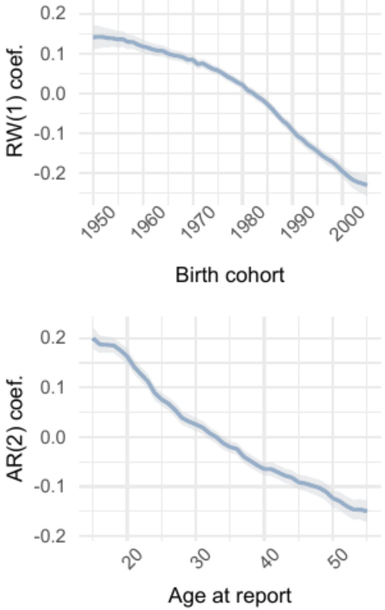

	Parameter	Estimate
1	intercept	-0.08 [-0.09 – -0.06]
2	skew	1.70 [1.61 – 1.81]
3	shape	5.52 [5.41 – 5.62]

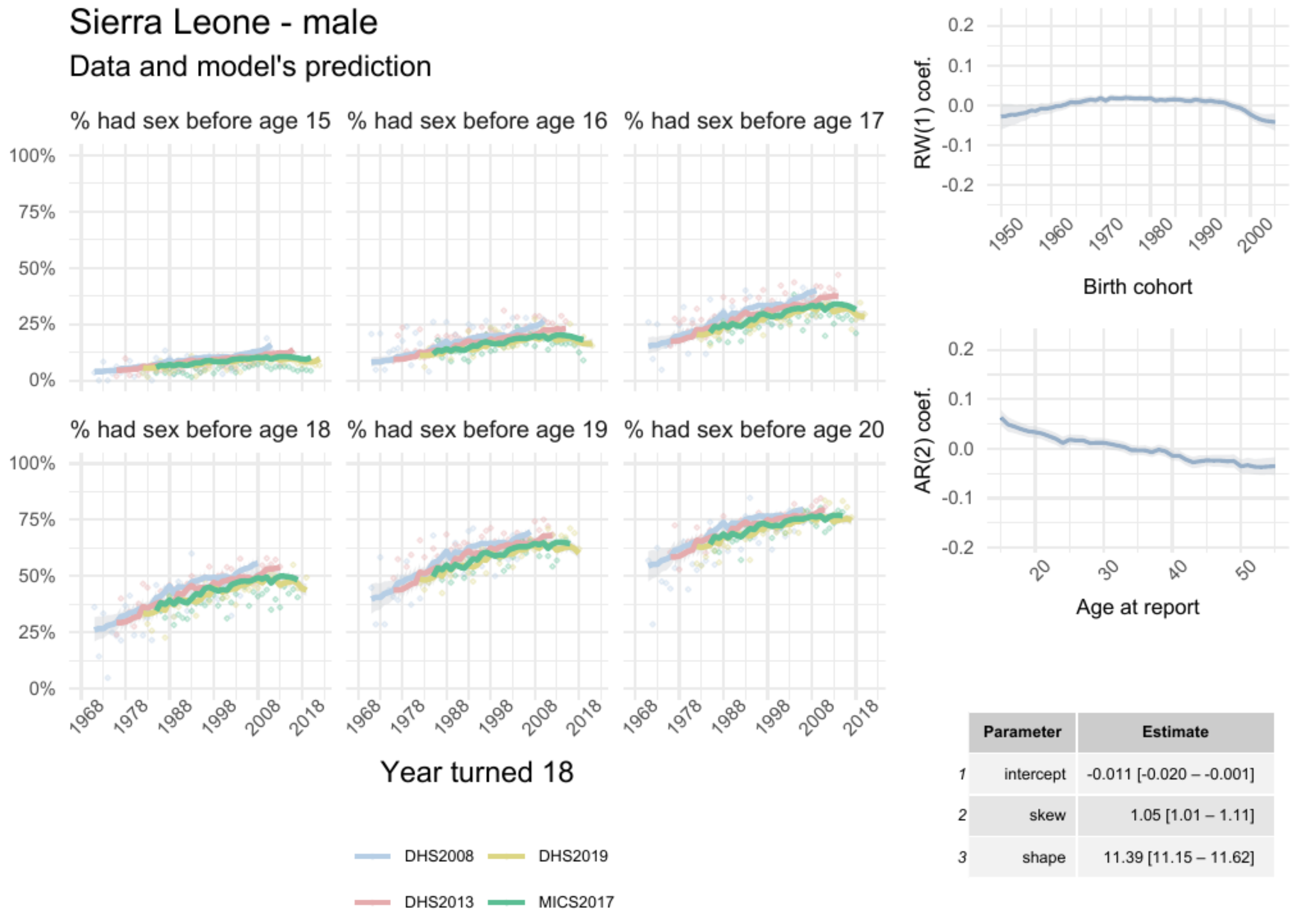

	Parameter	Estimate
1	intercept	-0.011 [-0.020 – -0.001]
2	skew	1.05 [1.01 – 1.11]
3	shape	11.39 [11.15 – 11.62]

São Tomé & Príncipe - male

Data and model's prediction

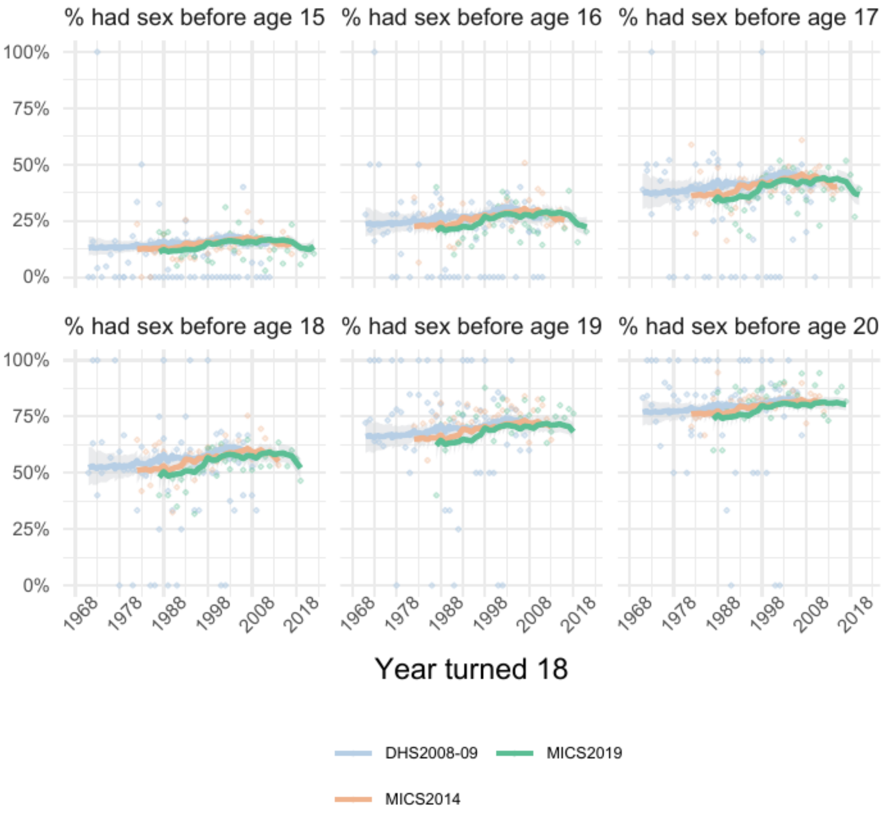

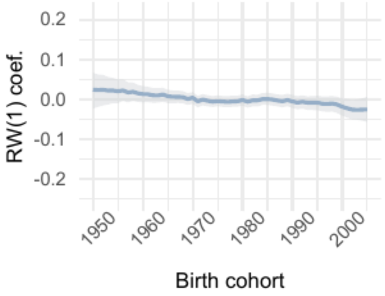

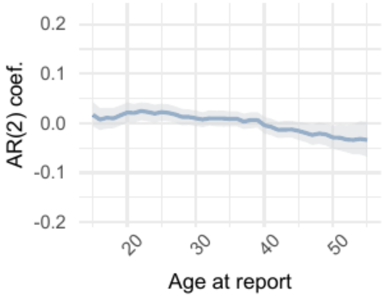

	Parameter	Estimate
1	intercept	0.05 [0.04 – 0.07]
2	skew	1.13 [1.04 – 1.23]
3	shape	10.32 [9.92 – 10.72]

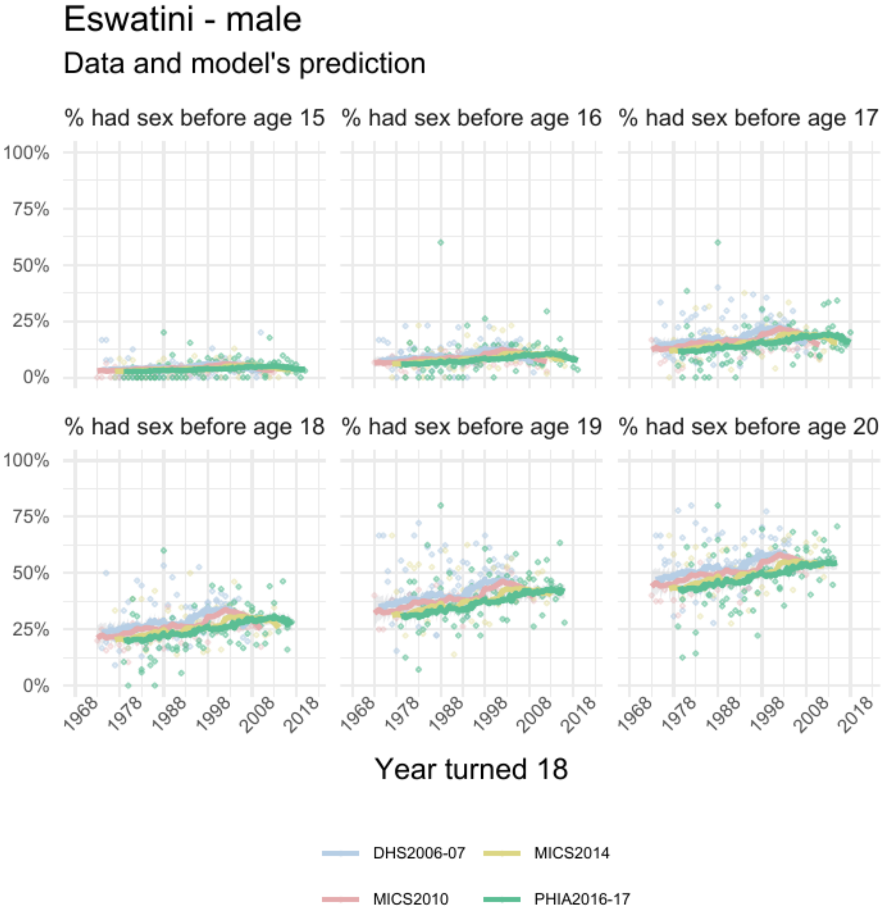

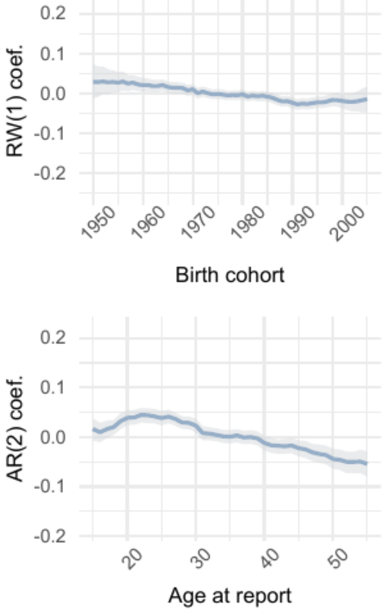

	Parameter	Estimate
1	intercept	0.012 [-0.005 – 0.028]
2	skew	1.90 [1.75 – 2.06]
3	shape	8.08 [7.86 – 8.30]

Chad - male

Data and model's prediction

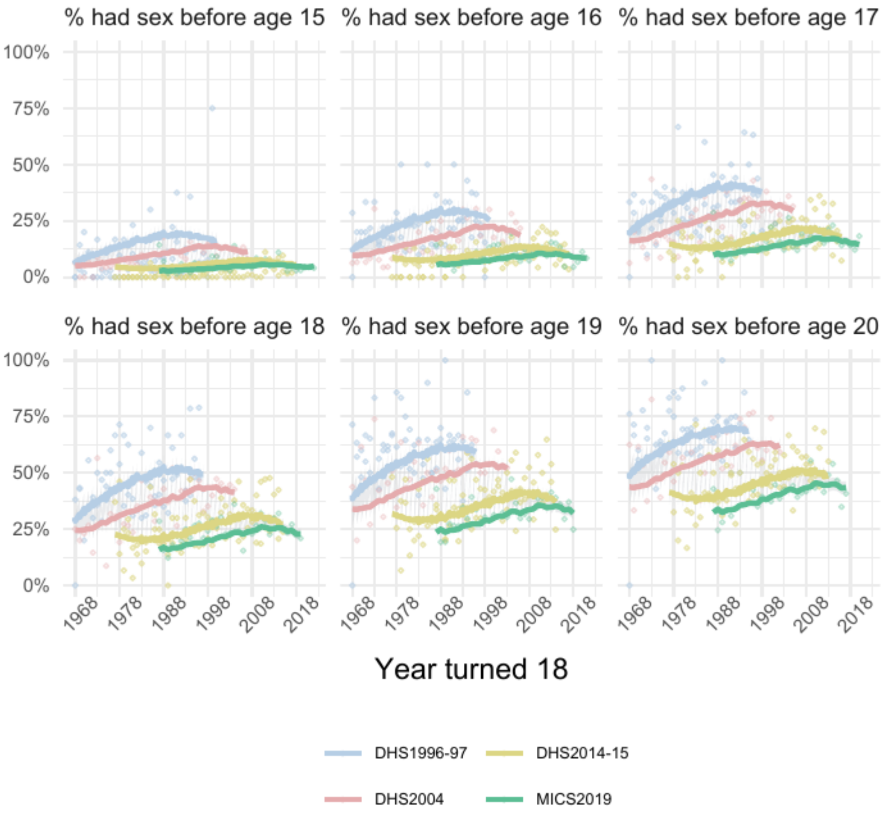

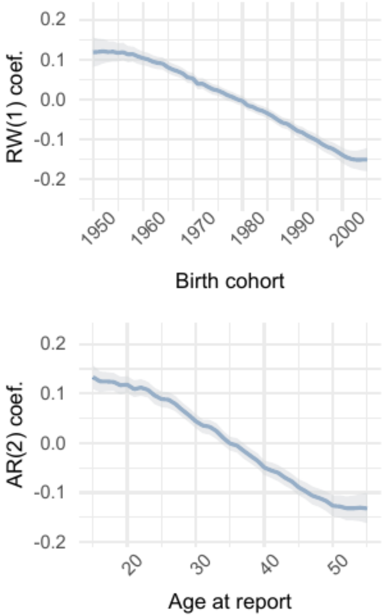

	Parameter	Estimate
1	intercept	0.04 [0.01 – 0.06]
2	skew	2.13 [1.93 – 2.34]
3	shape	6.42 [6.24 – 6.60]

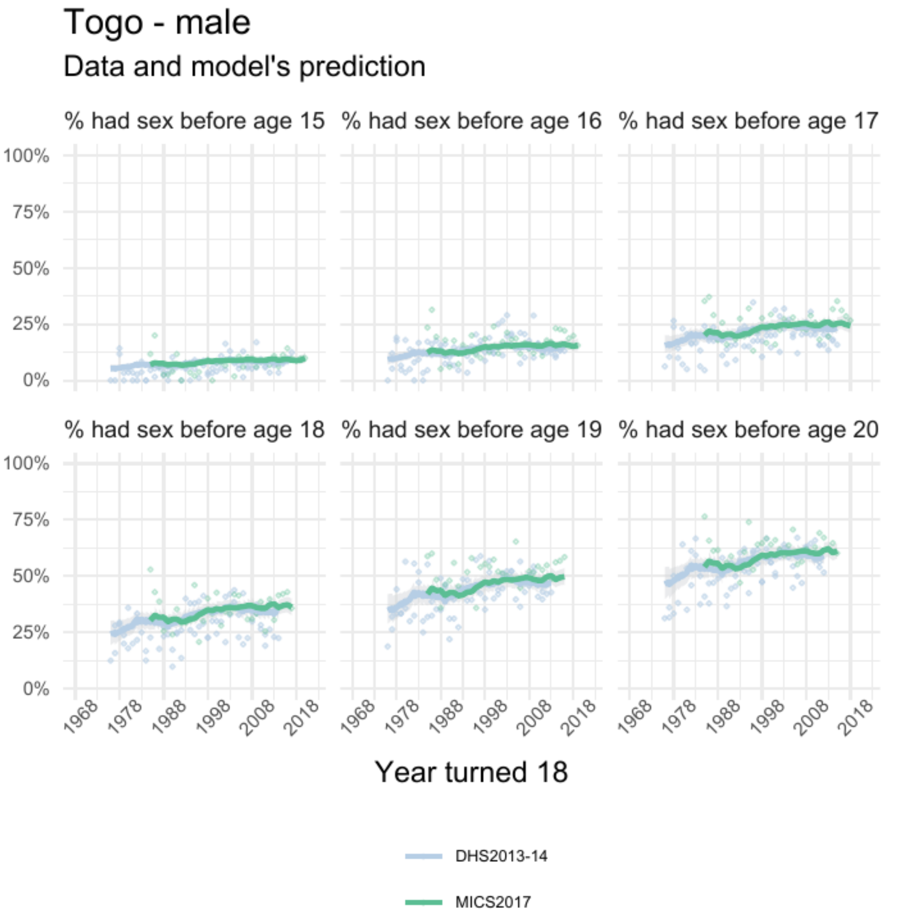

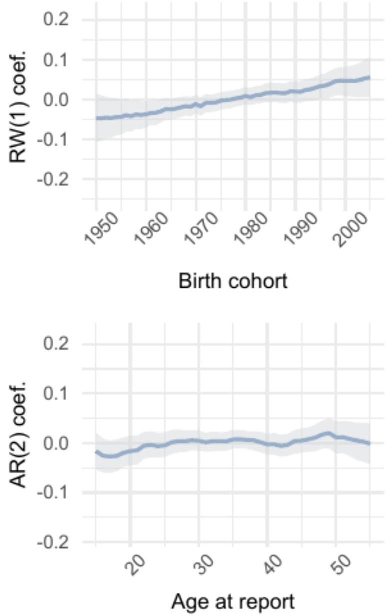

	Parameter	Estimate
1	intercept	-0.06 [-0.08 – -0.04]
2	skew	1.05 [0.96 – 1.14]
3	shape	9.30 [8.92 – 9.67]

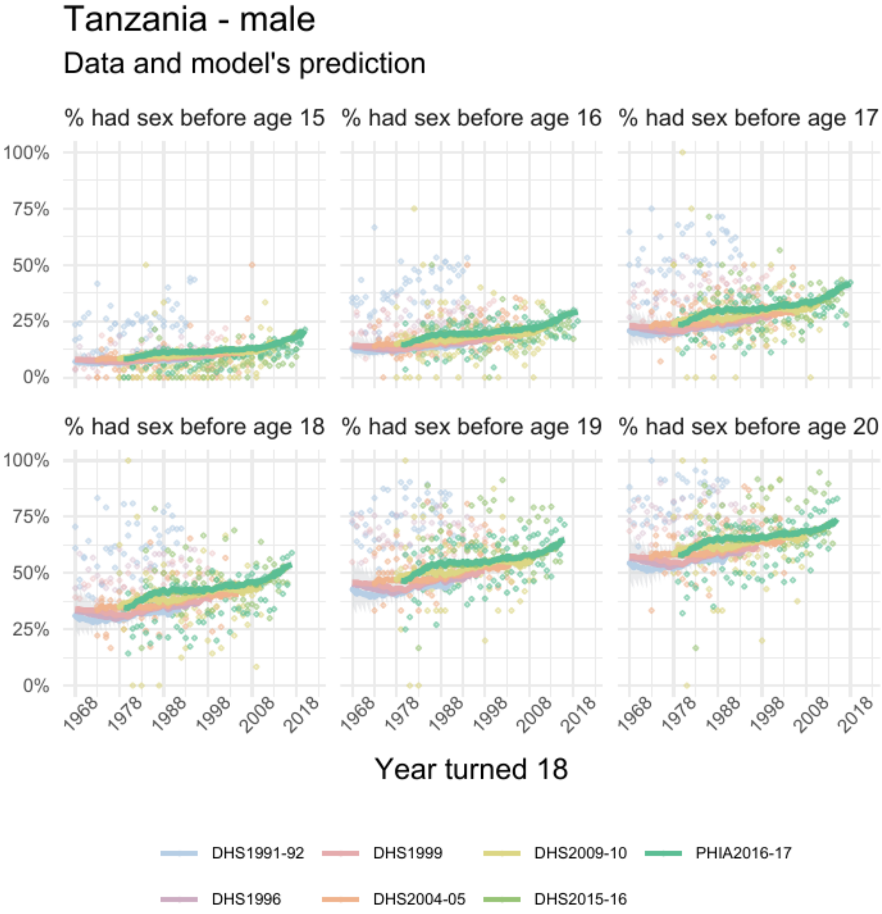

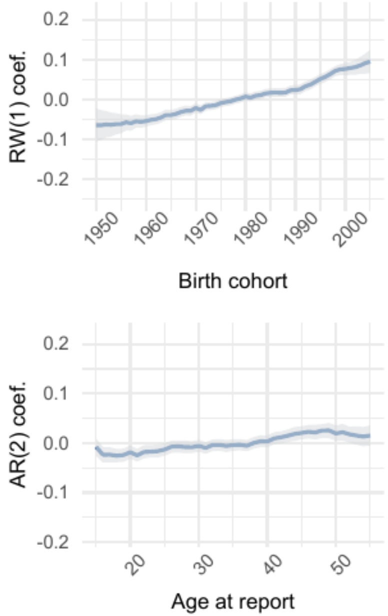

	Parameter	Estimate
1	intercept	0.017 [0.007 – 0.029]
2	skew	1.27 [1.20 – 1.33]
3	shape	8.50 [8.34 – 8.68]

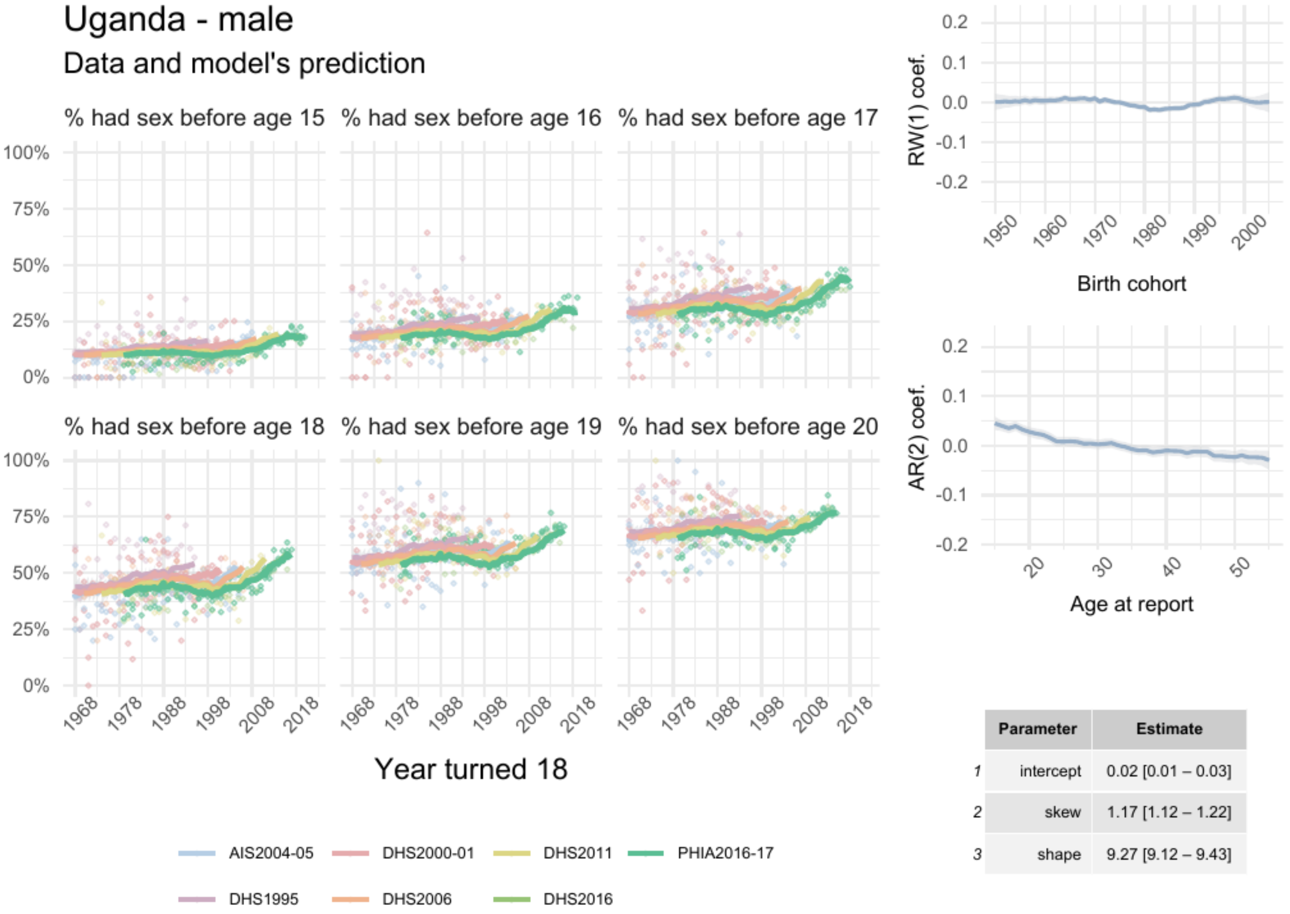

	Parameter	Estimate
1	intercept	0.02 [0.01 – 0.03]
2	skew	1.17 [1.12 – 1.22]
3	shape	9.27 [9.12 – 9.43]

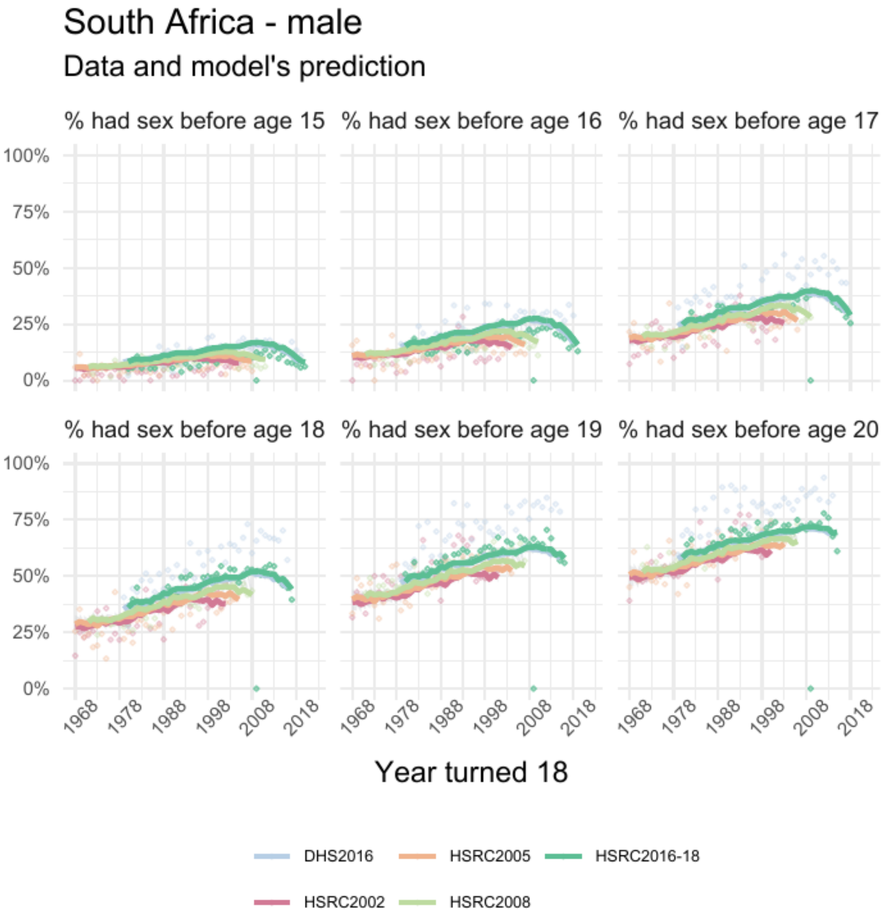

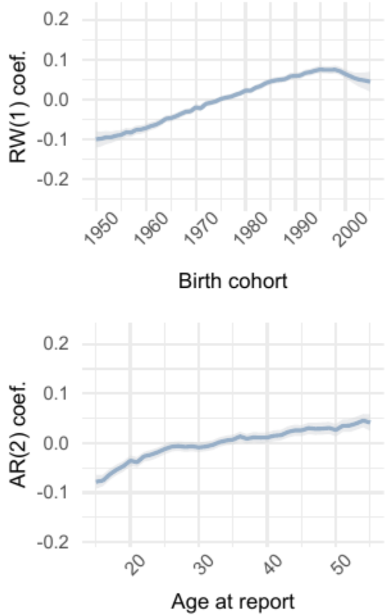

	Parameter	Estimate
1	intercept	0.08 [0.07 – 0.09]
2	skew	1.85 [1.78 – 1.92]
3	shape	7.37 [7.27 – 7.47]

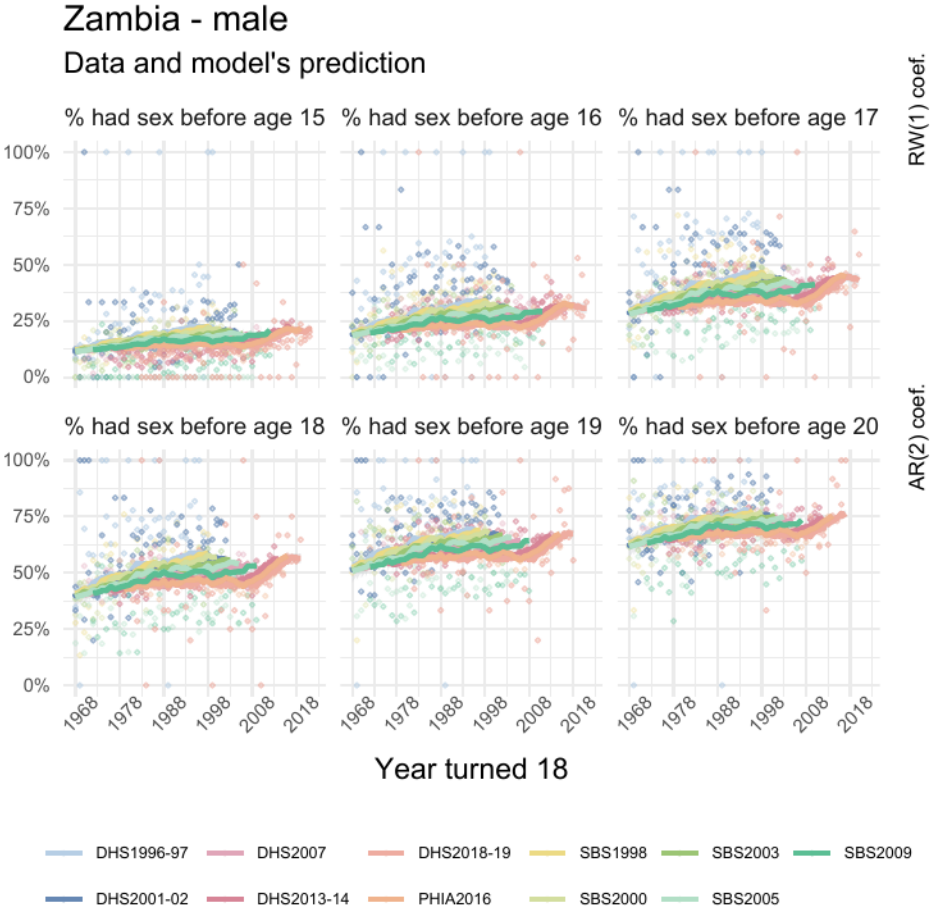

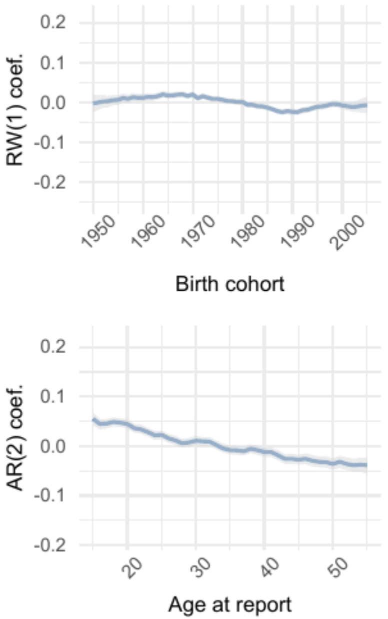

	Parameter	Estimate
1	intercept	0.02 [0.01 – 0.03]
2	skew	1.09 [1.05 – 1.12]
3	shape	8.46 [8.33 – 8.58]

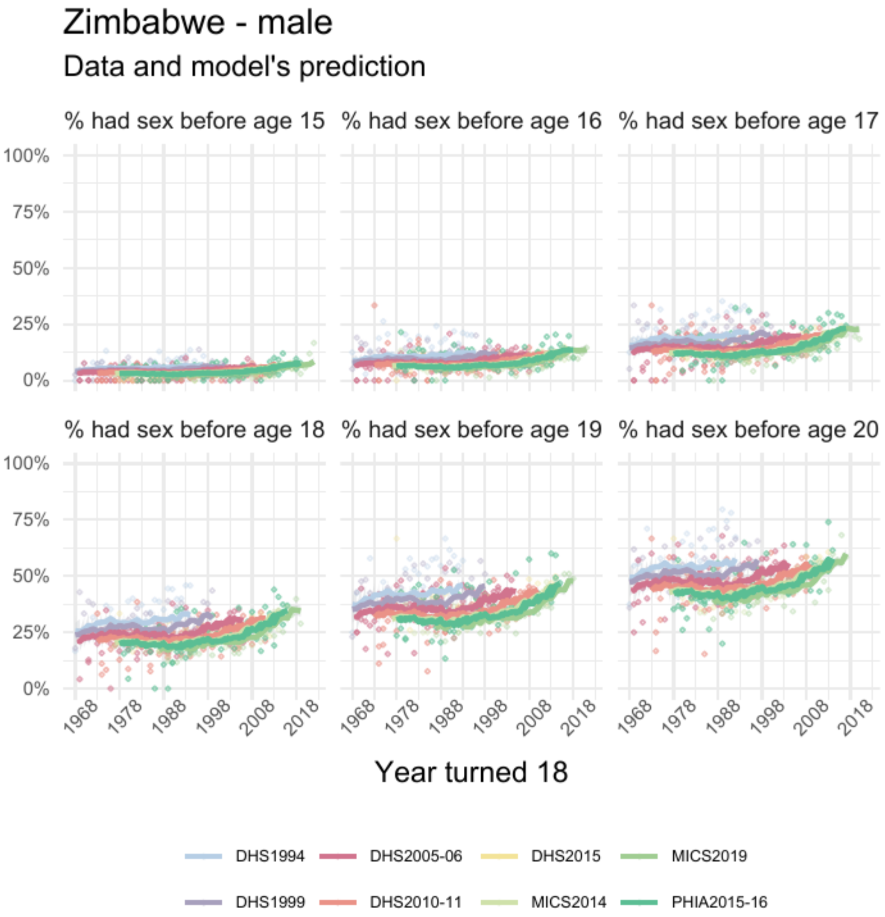

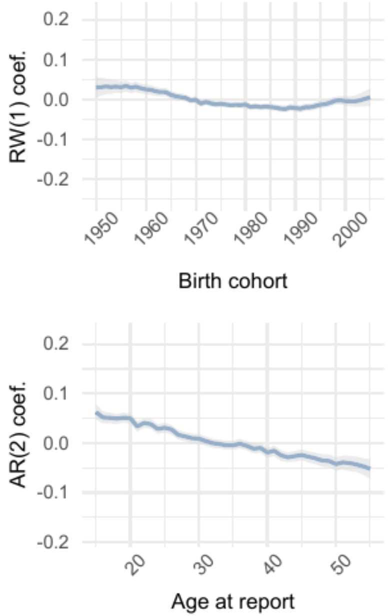

	Parameter	Estimate
1	intercept	-0.03 [-0.04 – -0.03]
2	skew	1.51 [1.45 – 1.57]
3	shape	8.61 [8.48 – 8.74]
