# Supplementary material for: Trends and country-level variation in age at first sex in sub-Saharan Africa among birth cohorts entering adulthood between 1985 and 2020
Source: BMC Public Health. 2022 Jun 4;22:1120. doi: 10.1186/s12889-022-13451-y (PMC9167540; doi:10.1186/s12889-022-13451-y)
Supplement: Supplementary file 3 — Additional file 3. [file 12889_2022_13451_MOESM3_ESM.pdf]

Angola - female

Data and model's prediction

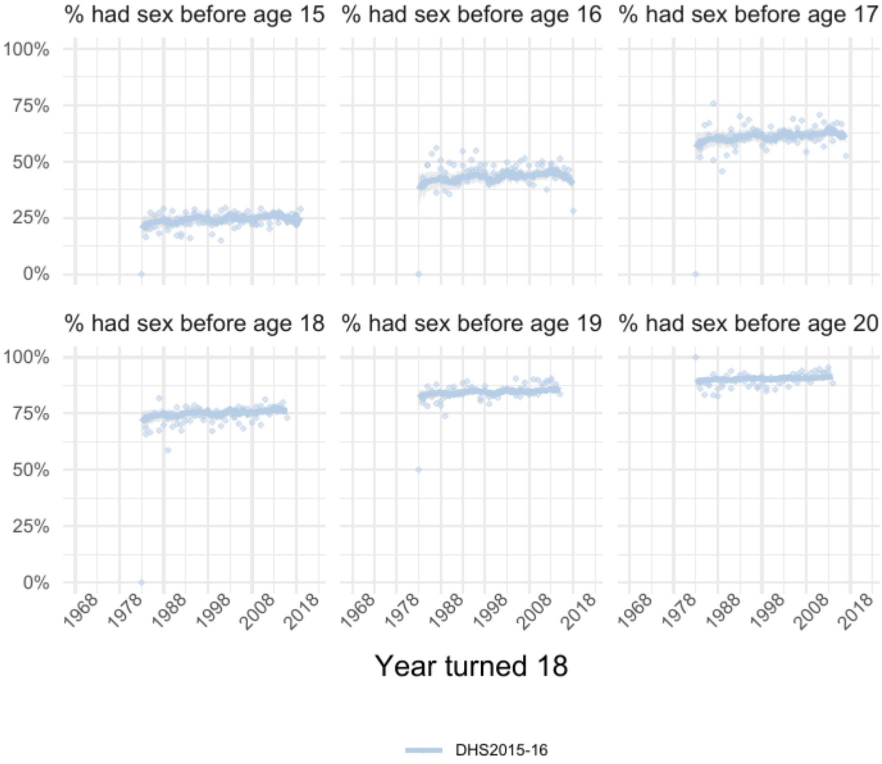

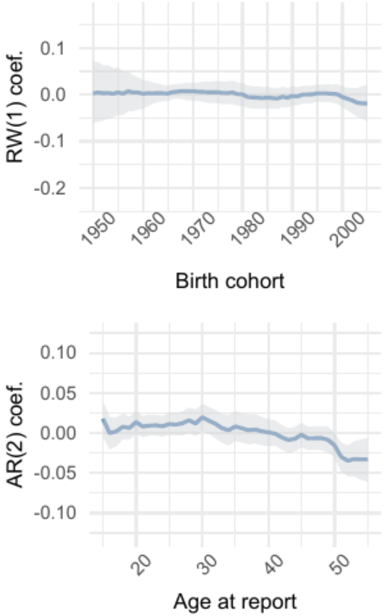

	Parameter	Estimate
1	intercept	0.06 [0.04 – 0.08]
2	skew	1.94 [1.80 – 2.11]
3	shape	10.46 [10.21 – 10.69]

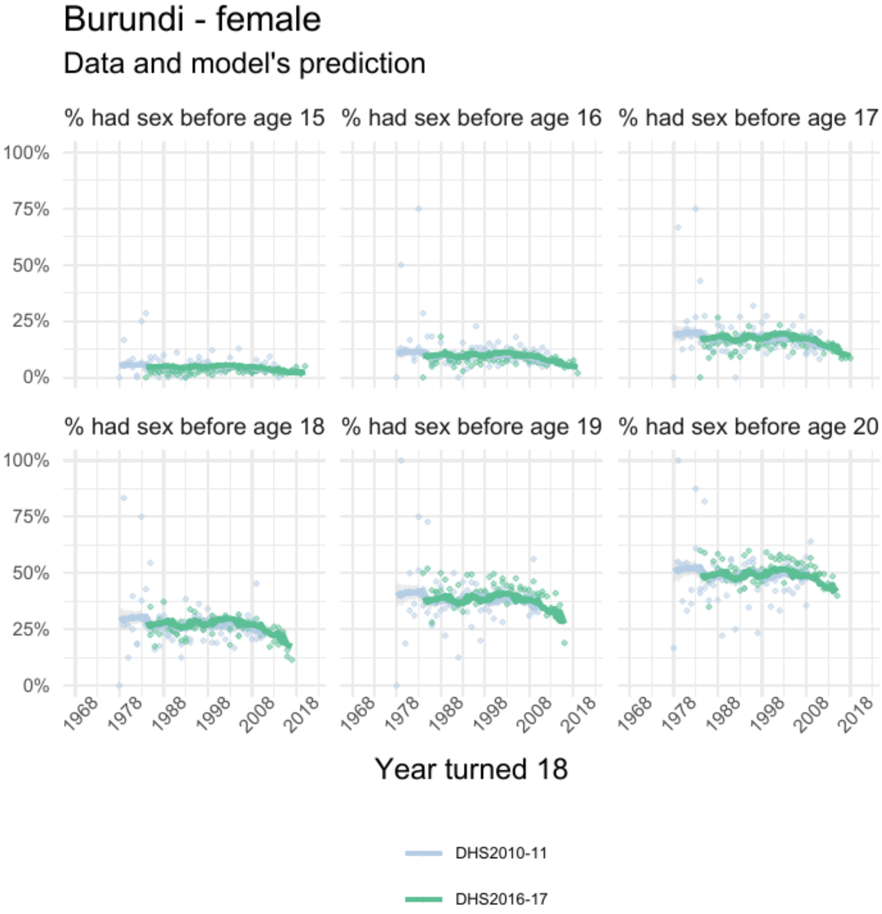

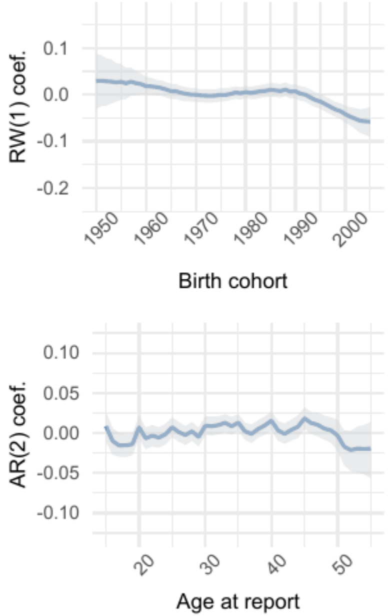

	Parameter	Estimate
1	intercept	-0.06 [-0.07 – -0.04]
2	skew	2.59 [2.46 – 2.73]
3	shape	6.83 [6.73 – 6.92]

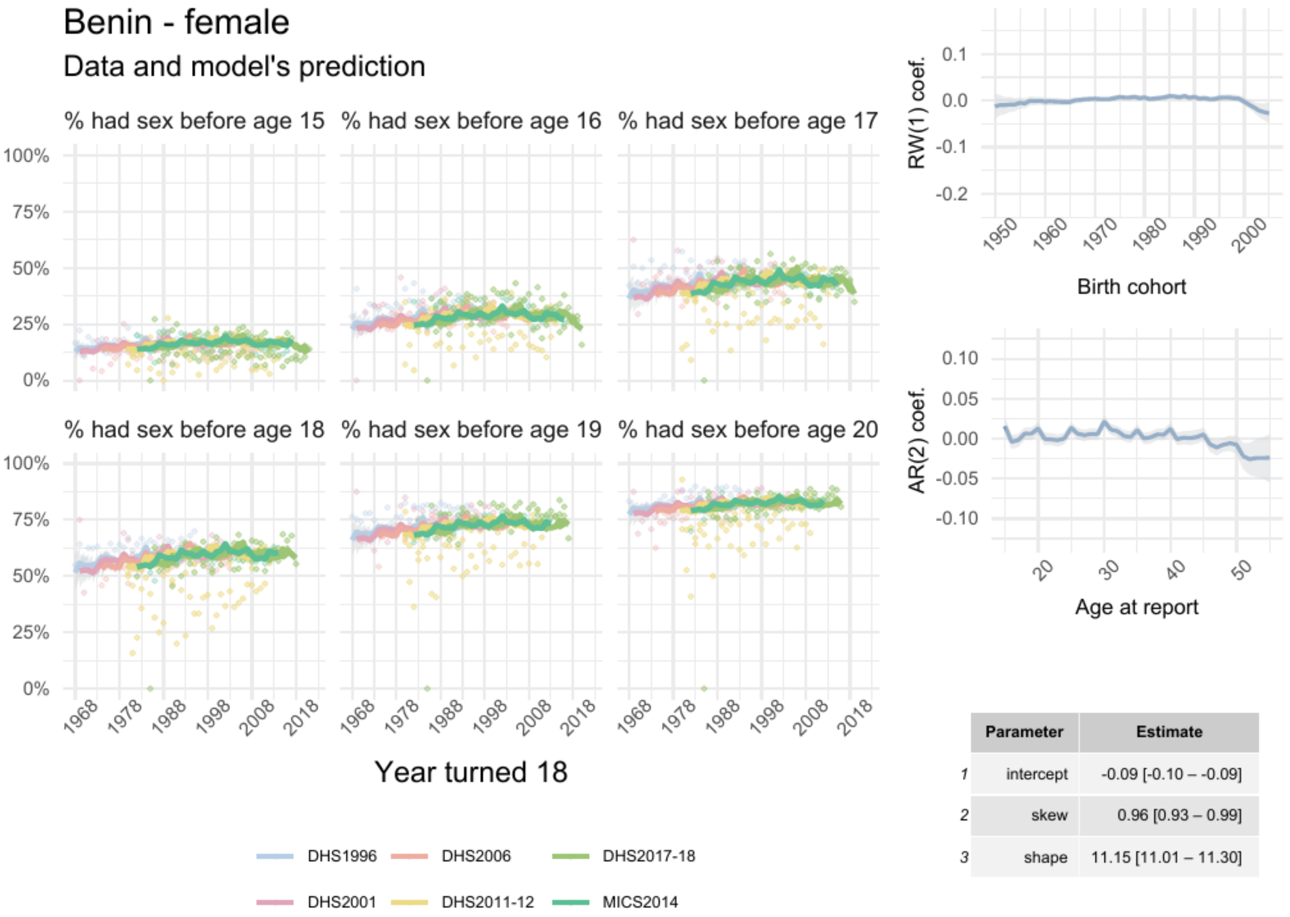

	Parameter	Estimate
1	intercept	-0.09 [-0.10 – -0.09]
2	skew	0.96 [0.93 – 0.99]
3	shape	11.15 [11.01 – 11.30]

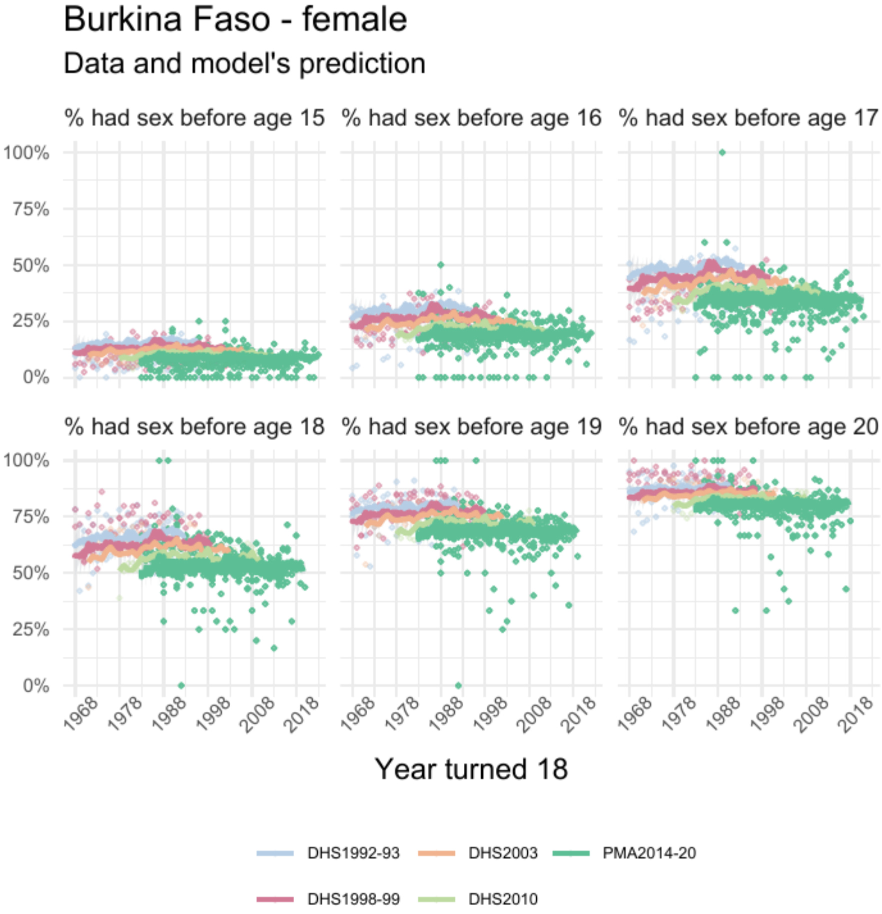

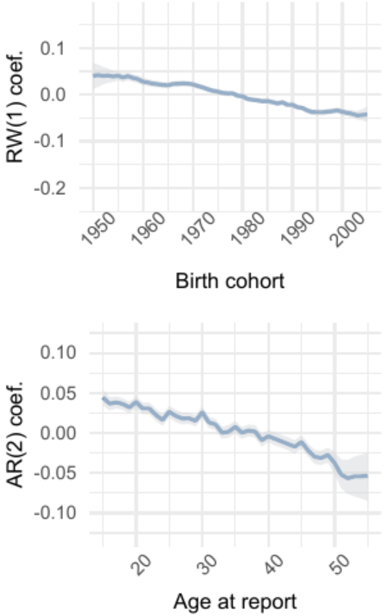

	Parameter	Estimate
1	intercept	-0.08 [-0.09 – -0.07]
2	skew	1.21 [1.17 – 1.25]
3	shape	12.16 [12.01 – 12.32]

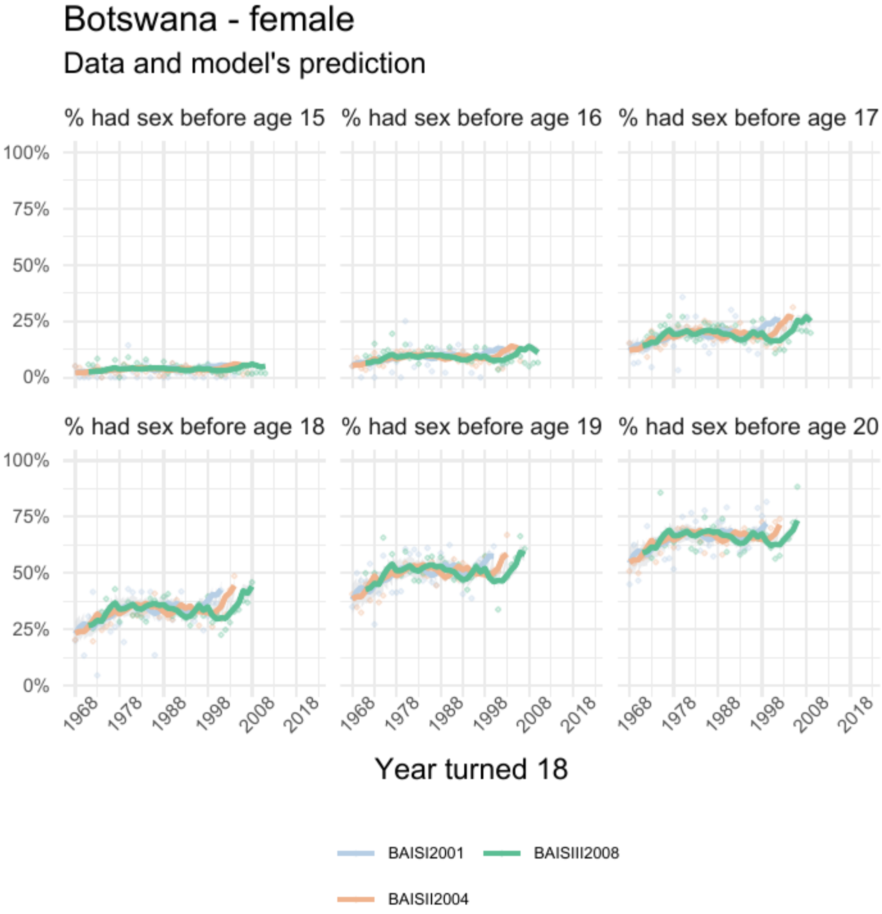

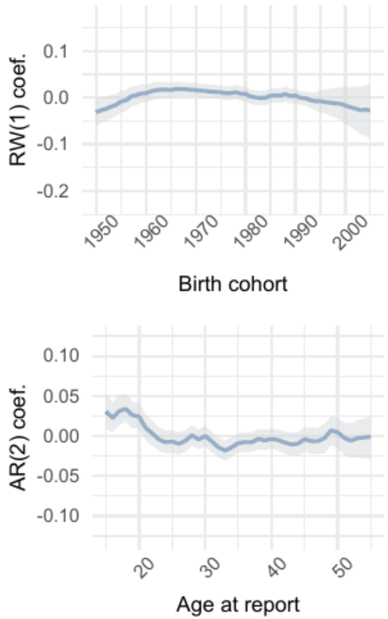

	Parameter	Estimate
1	intercept	-0.14 [-0.15 – -0.13]
2	skew	1.31 [1.23 – 1.40]
3	shape	11.92 [11.64 – 12.21]

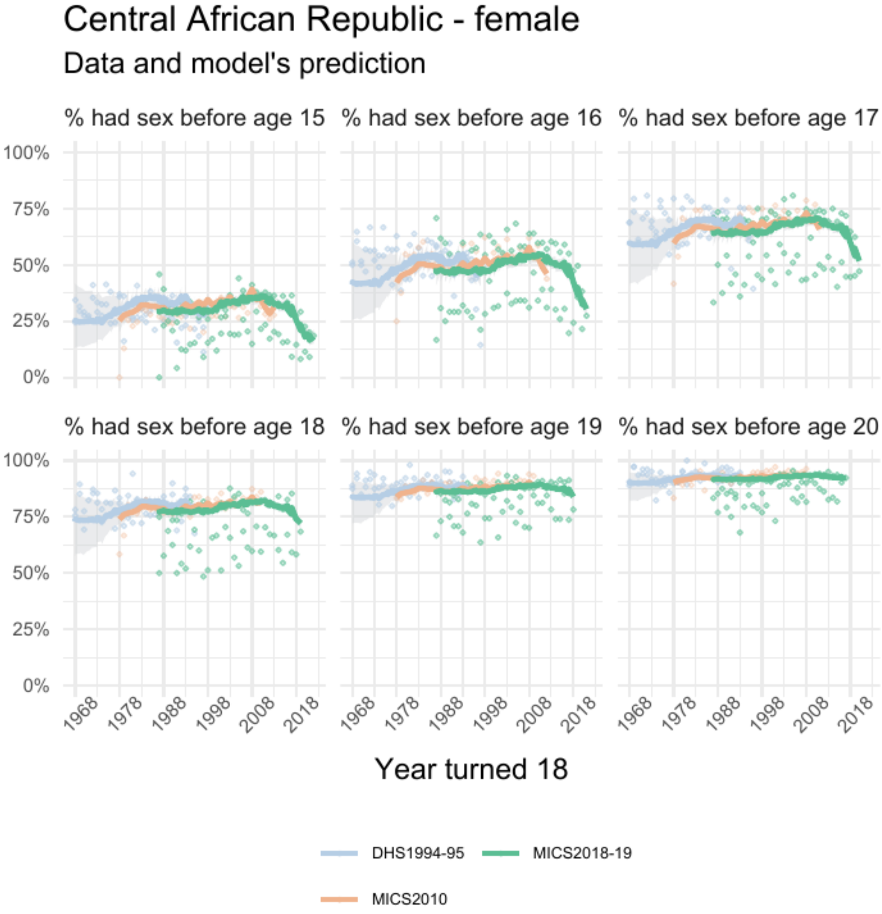

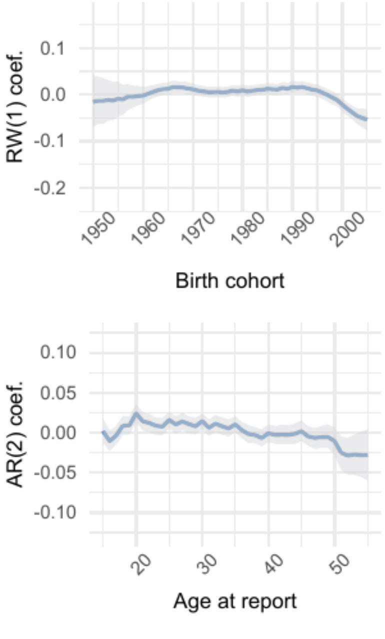

	Parameter	Estimate
1	intercept	0.016 [0.004 – 0.028]
2	skew	1.24 [1.17 – 1.30]
3	shape	10.95 [10.73 – 11.18]

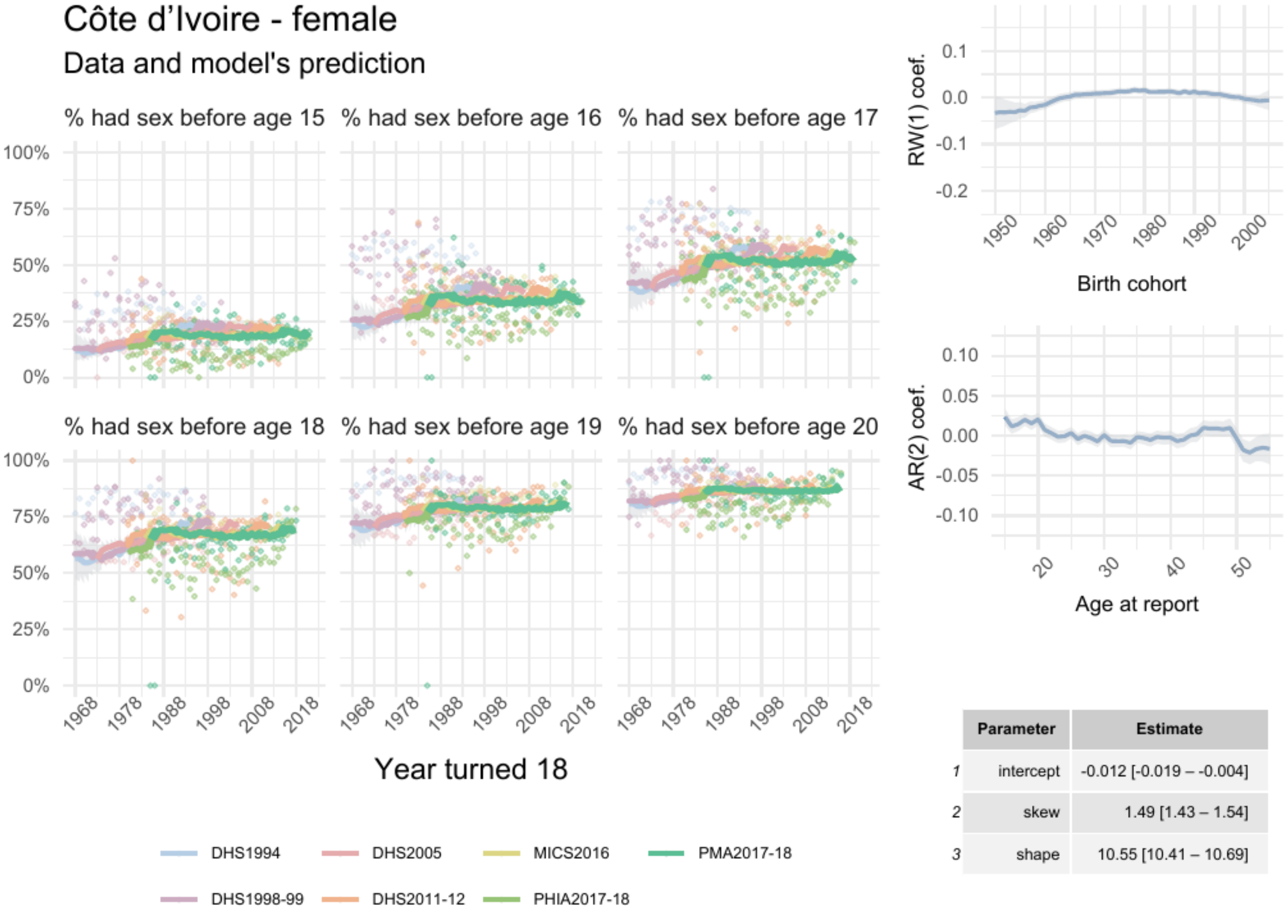

	Parameter	Estimate
1	intercept	-0.012 [-0.019 – -0.004]
2	skew	1.49 [1.43 – 1.54]
3	shape	10.55 [10.41 – 10.69]

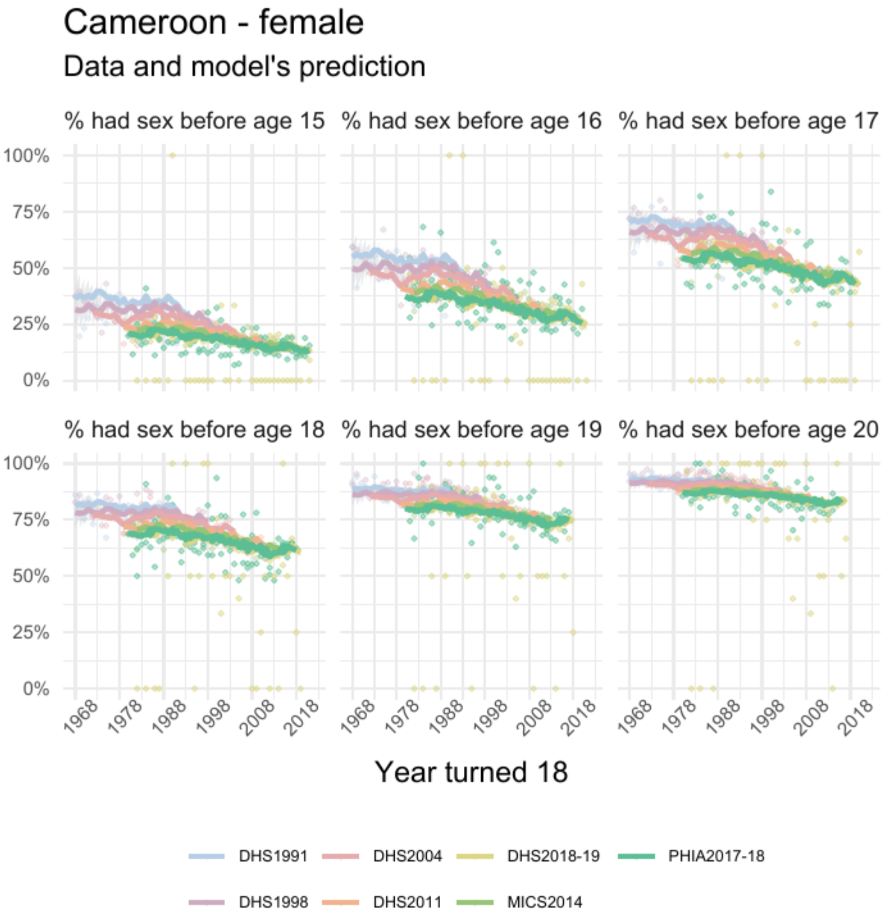

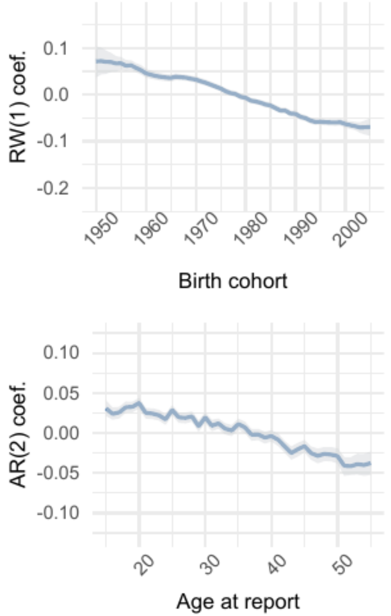

	Parameter	Estimate
1	intercept	0.03 [0.02 – 0.03]
2	skew	1.70 [1.64 – 1.76]
3	shape	10.04 [9.93 – 10.17]

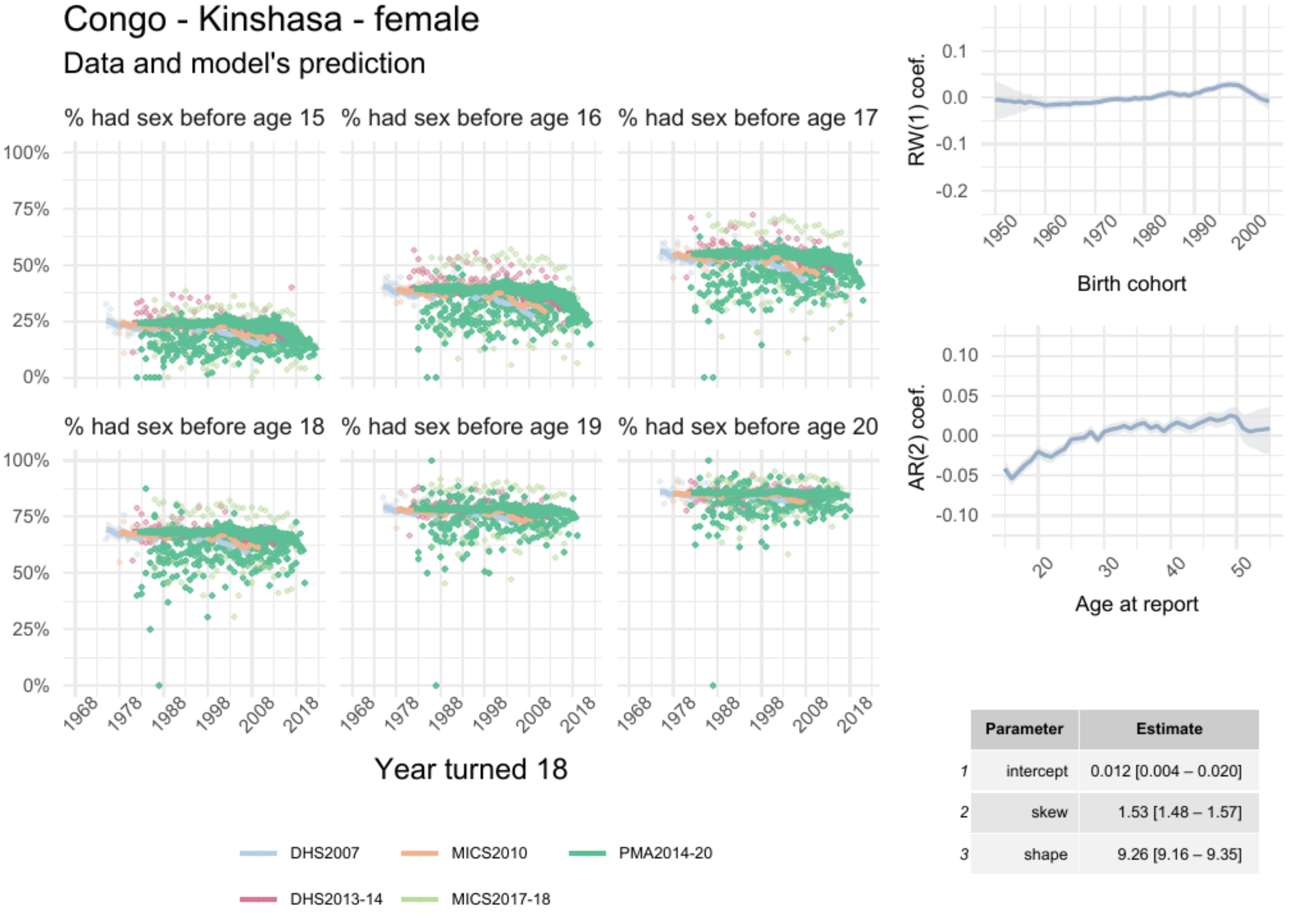

	Parameter	Estimate
1	intercept	0.012 [0.004 – 0.020]
2	skew	1.53 [1.48 – 1.57]
3	shape	9.26 [9.16 – 9.35]

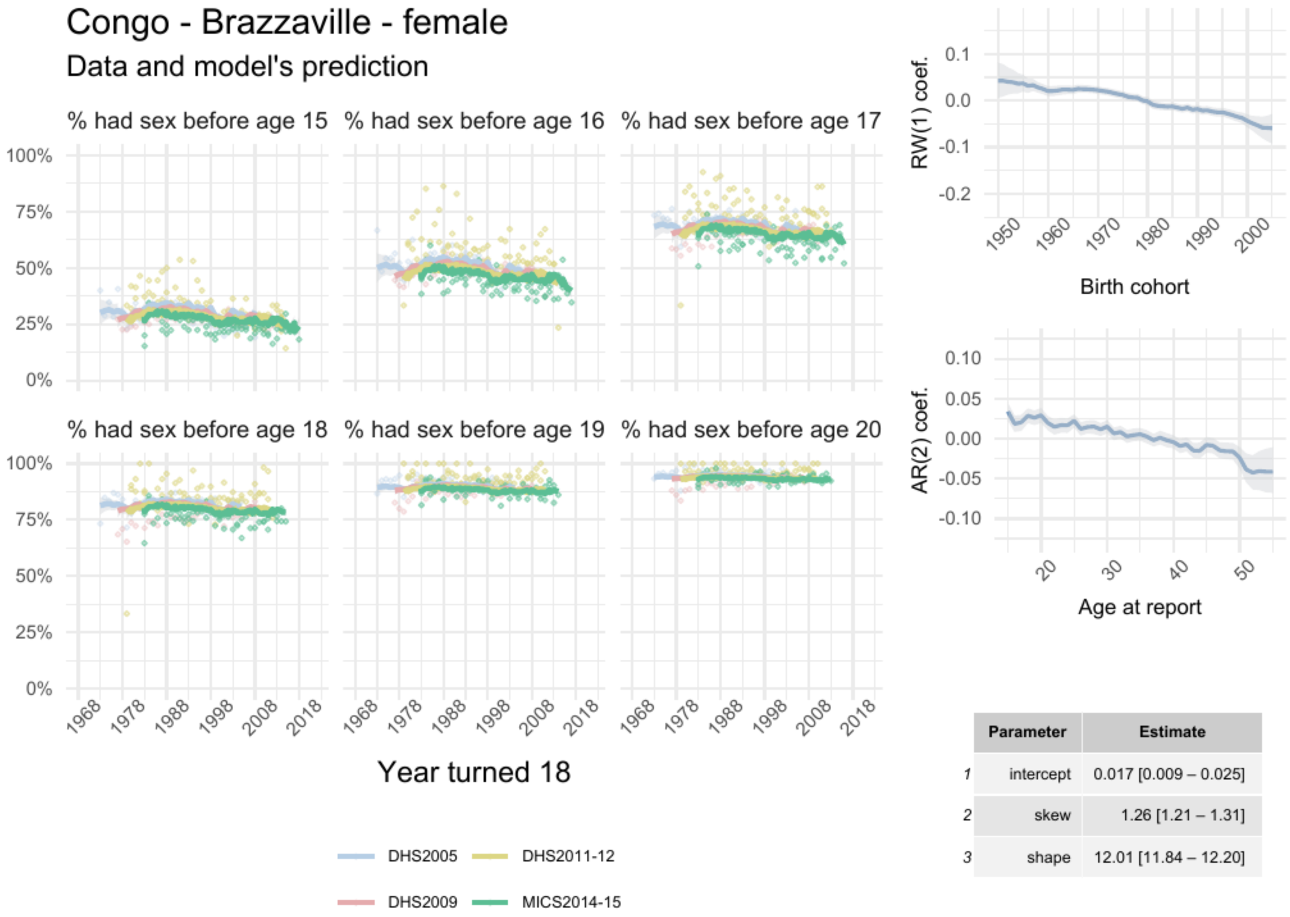

	Parameter	Estimate
1	intercept	0.017 [0.009 – 0.025]
2	skew	1.26 [1.21 – 1.31]
3	shape	12.01 [11.84 – 12.20]

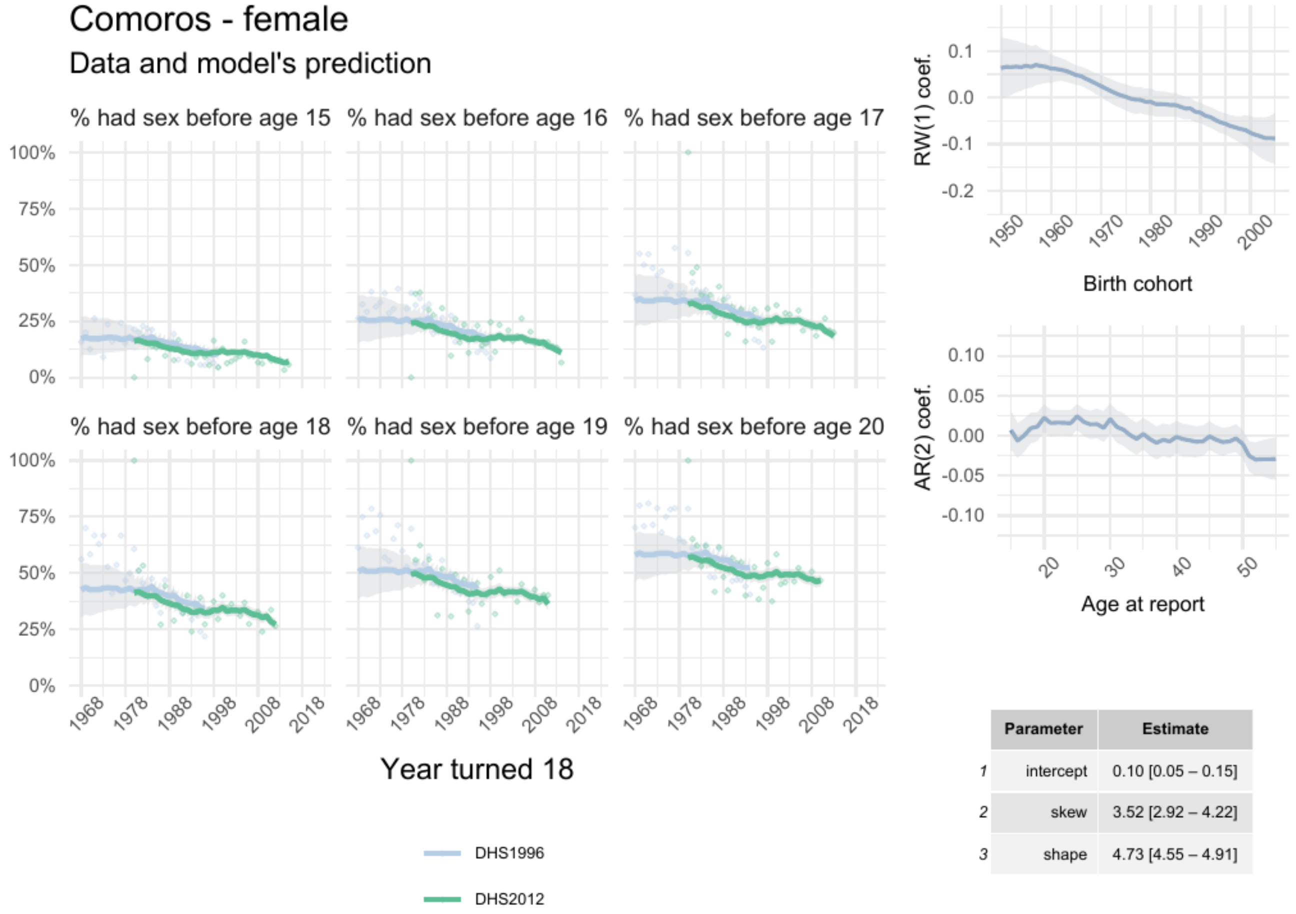

	Parameter	Estimate
1	intercept	0.10 [0.05 – 0.15]
2	skew	3.52 [2.92 – 4.22]
3	shape	4.73 [4.55 – 4.91]

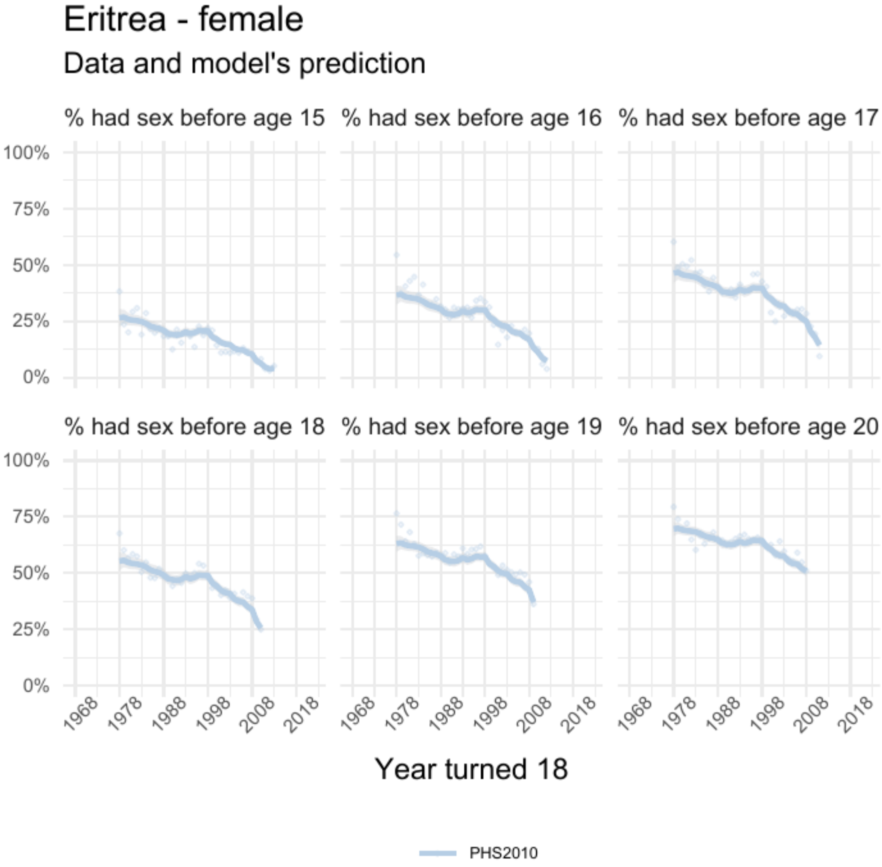

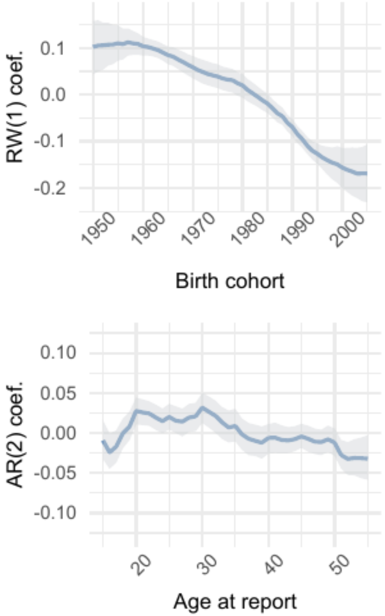

	Parameter	Estimate
1	intercept	0.16 [0.12 – 0.21]
2	skew	3.98 [3.43 – 4.66]
3	shape	4.96 [4.83 – 5.10]

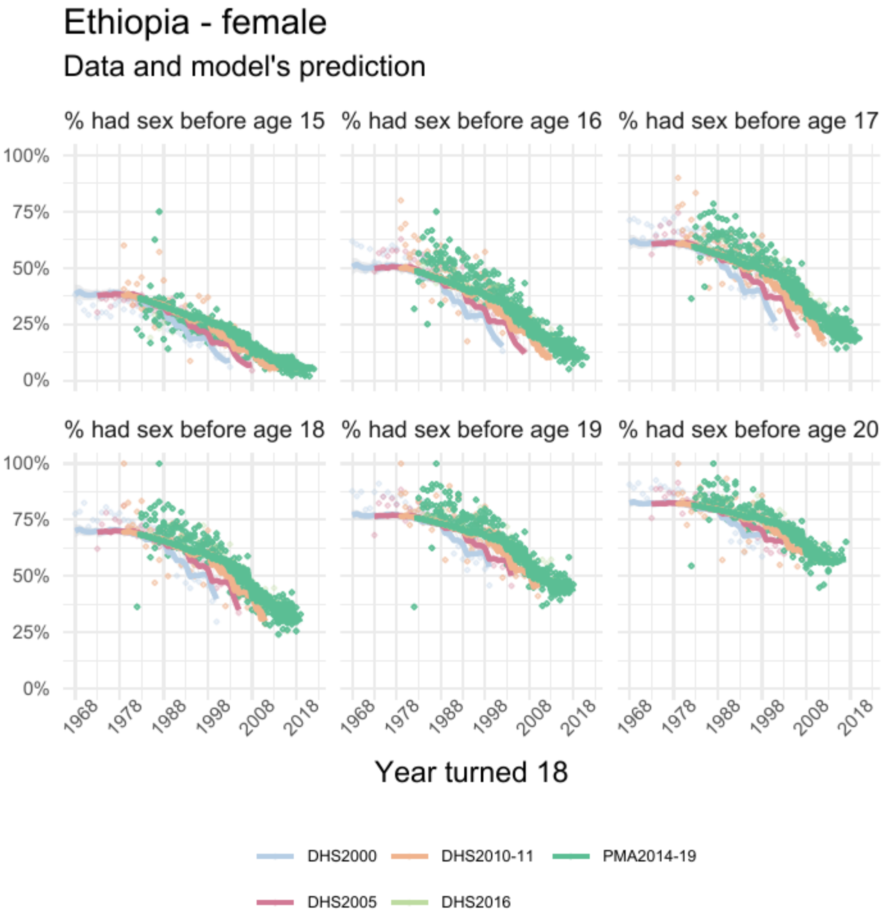

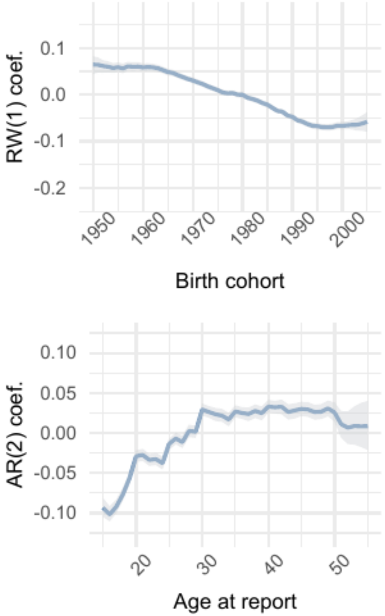

	Parameter	Estimate
1	intercept	0.10 [0.09 – 0.11]
2	skew	2.44 [2.37 – 2.52]
3	shape	6.13 [6.07 – 6.18]

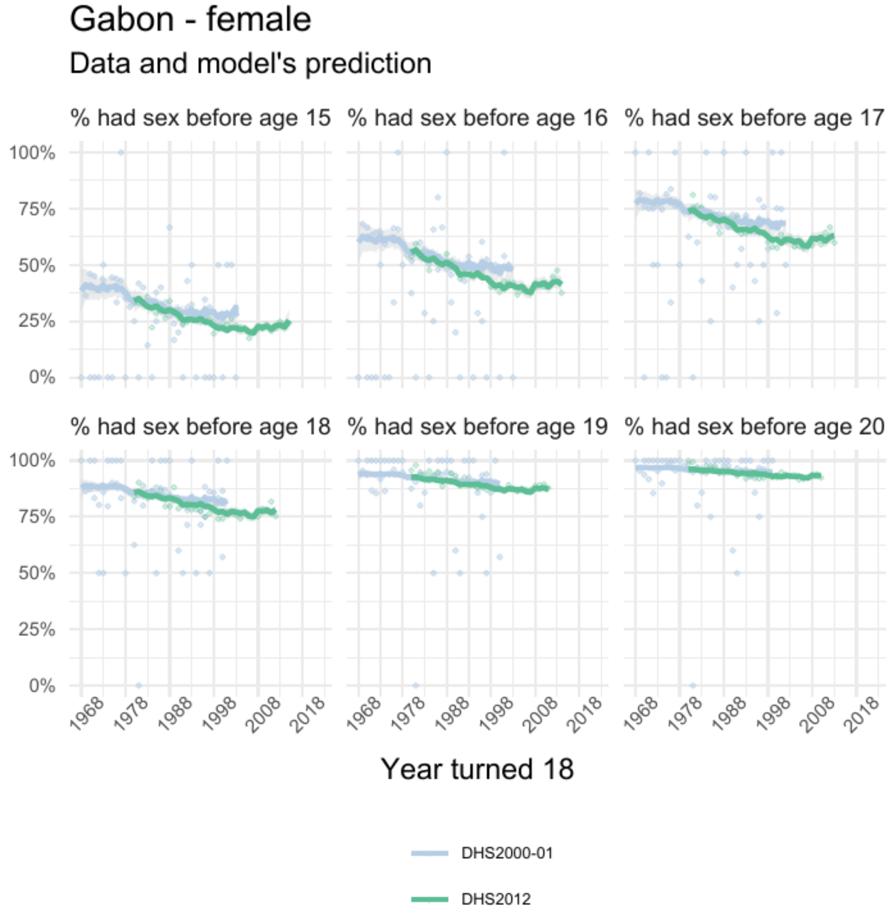

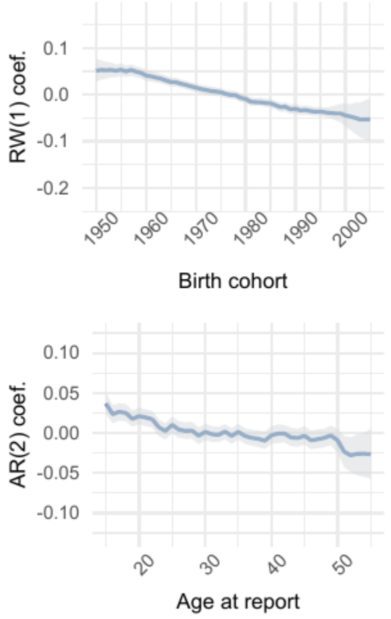

	Parameter	Estimate
1	intercept	0.007 [-0.004 – 0.016]
2	skew	1.15 [1.08 – 1.22]
3	shape	13.07 [12.77 – 13.41]

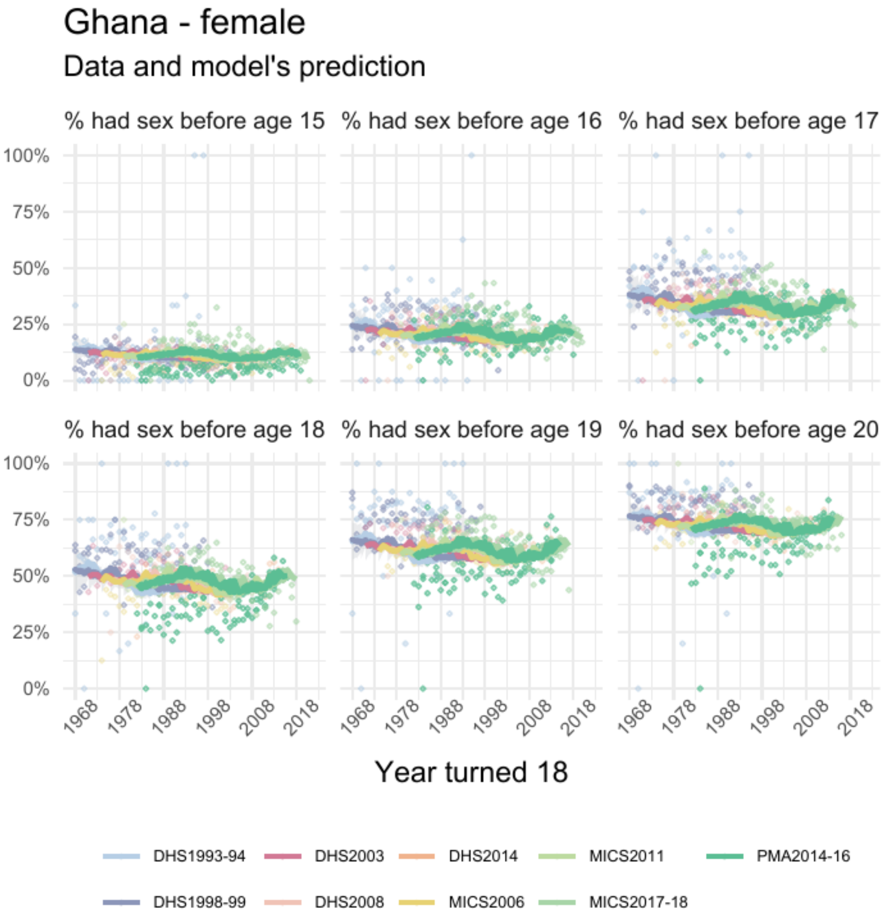

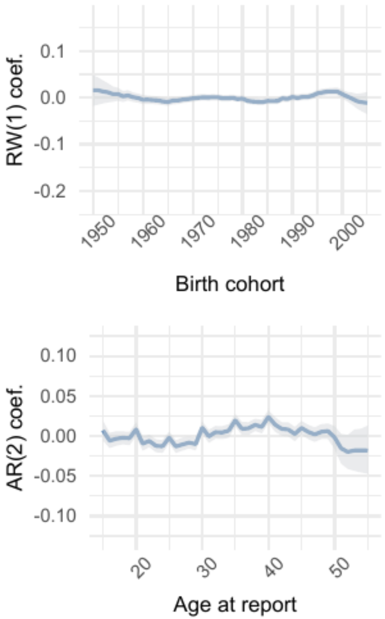

	Parameter	Estimate
1	intercept	-0.10 [-0.11 – -0.09]
2	skew	1.19 [1.16 – 1.23]
3	shape	9.88 [9.77 – 10.02]

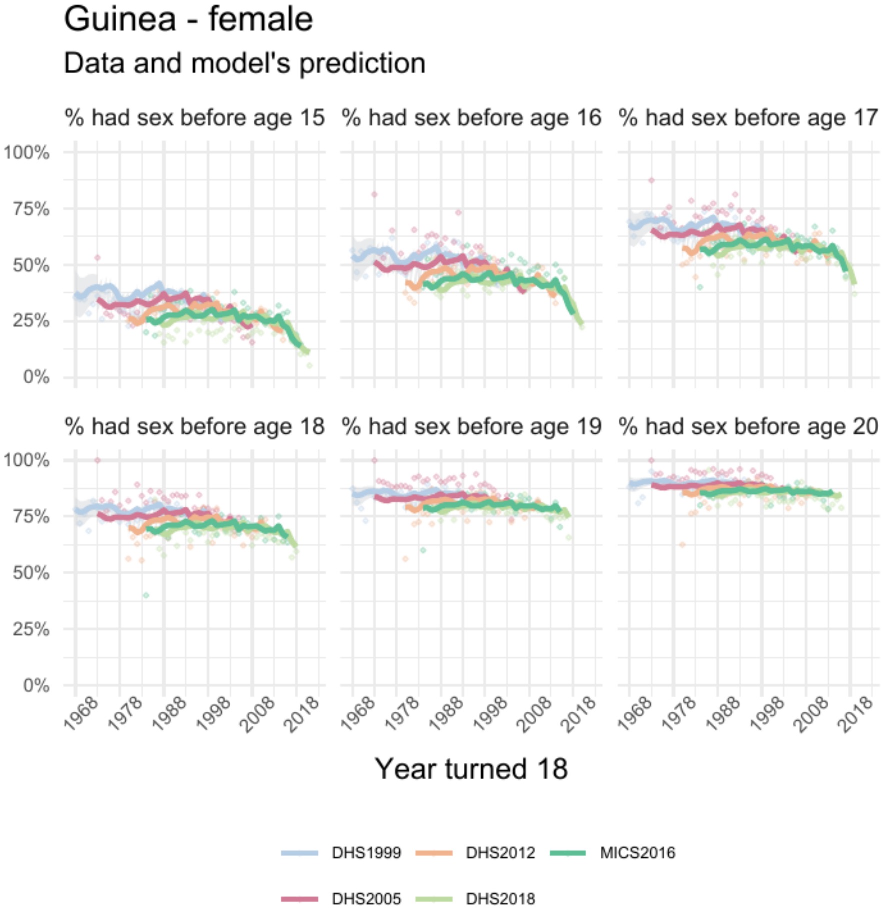

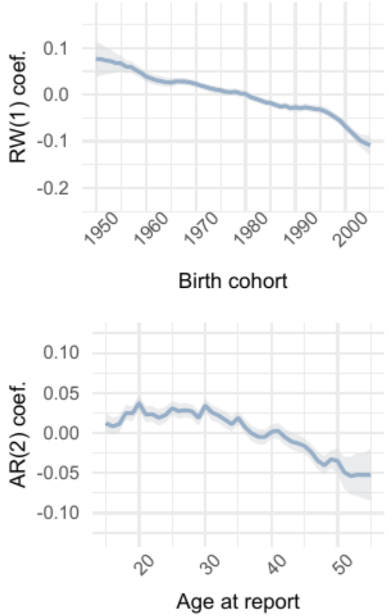

	Parameter	Estimate
1	intercept	0.11 [0.10 – 0.12]
2	skew	2.45 [2.32 – 2.61]
3	shape	8.41 [8.29 – 8.53]

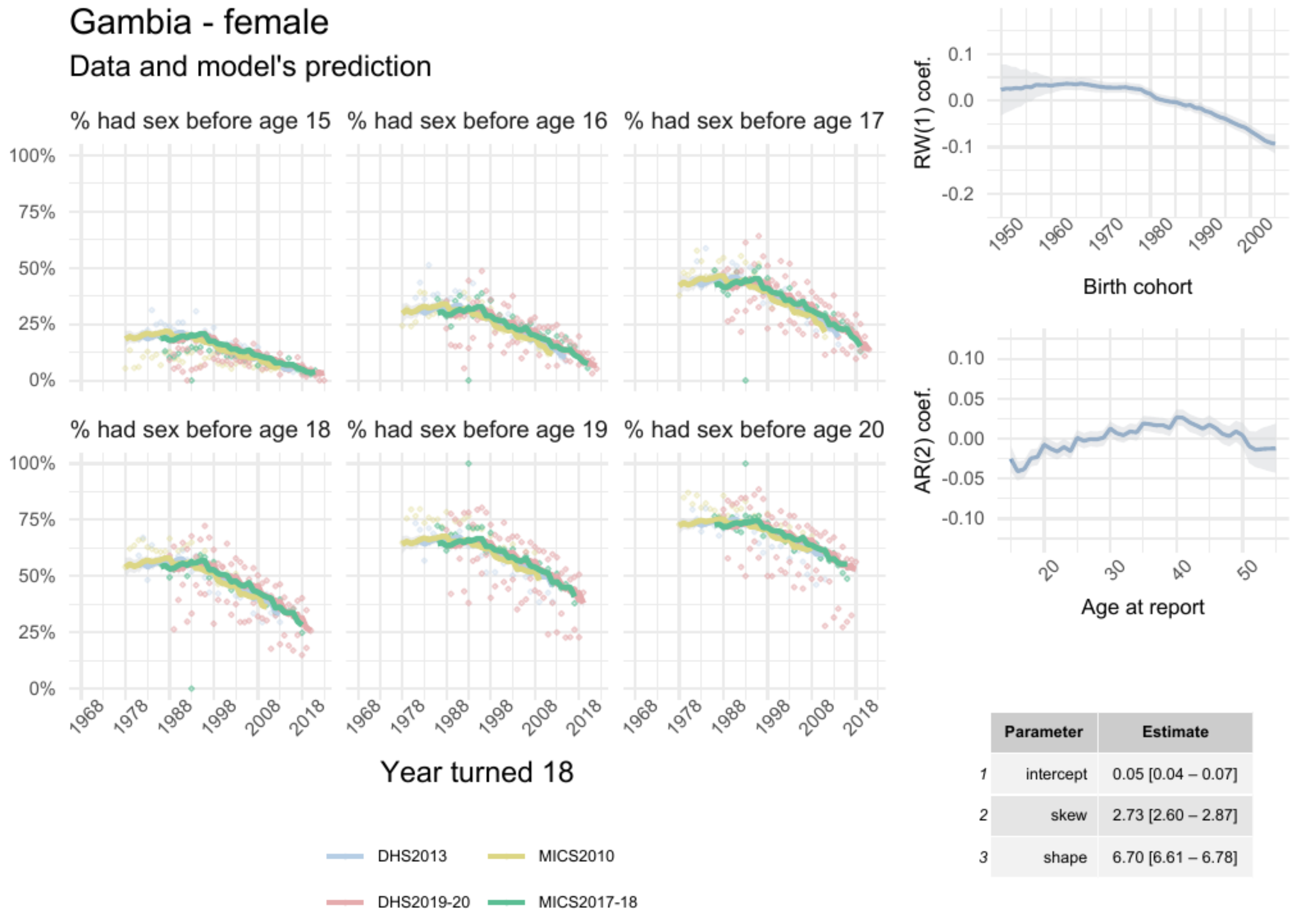

	Parameter	Estimate
1	intercept	0.05 [0.04 – 0.07]
2	skew	2.73 [2.60 – 2.87]
3	shape	6.70 [6.61 – 6.78]

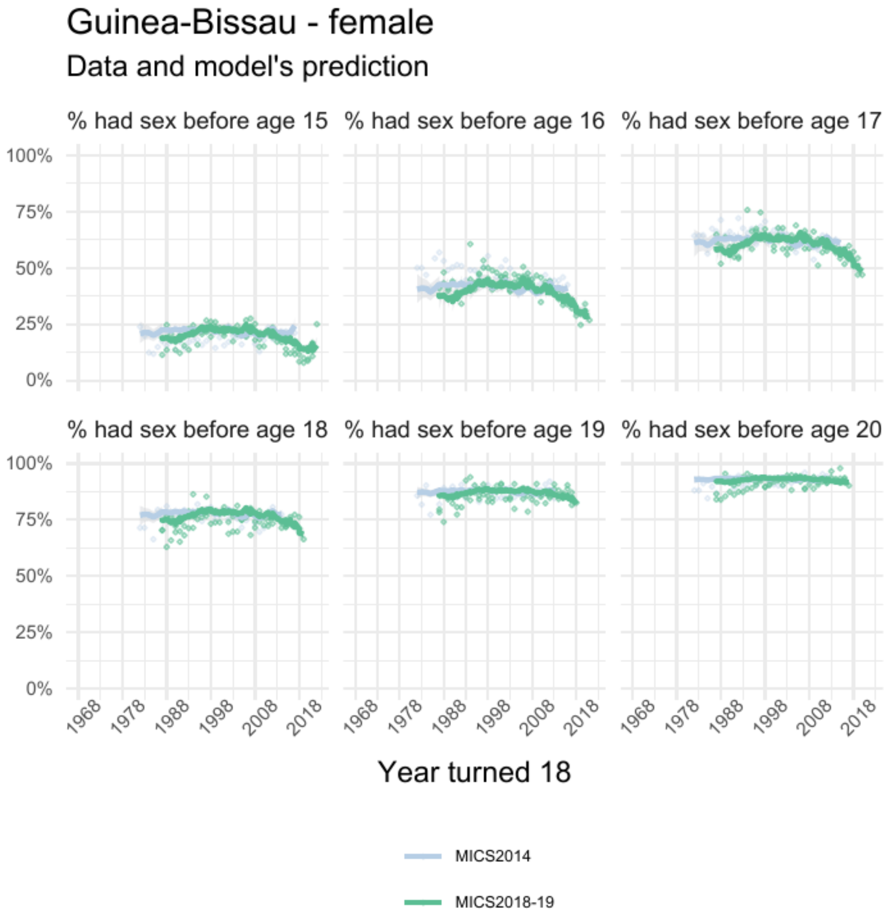

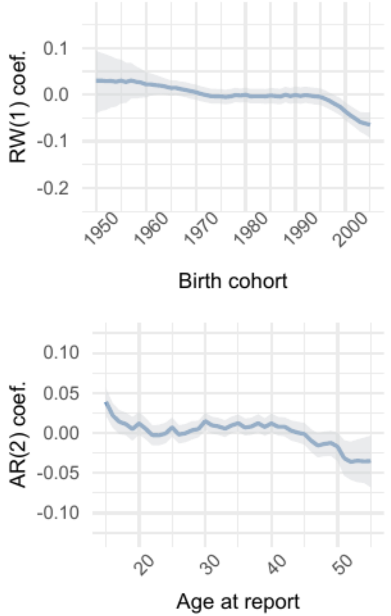

	Parameter	Estimate
1	intercept	0.013 [-0.002 – 0.026]
2	skew	1.42 [1.35 – 1.49]
3	shape	12.62 [12.39 – 12.86]

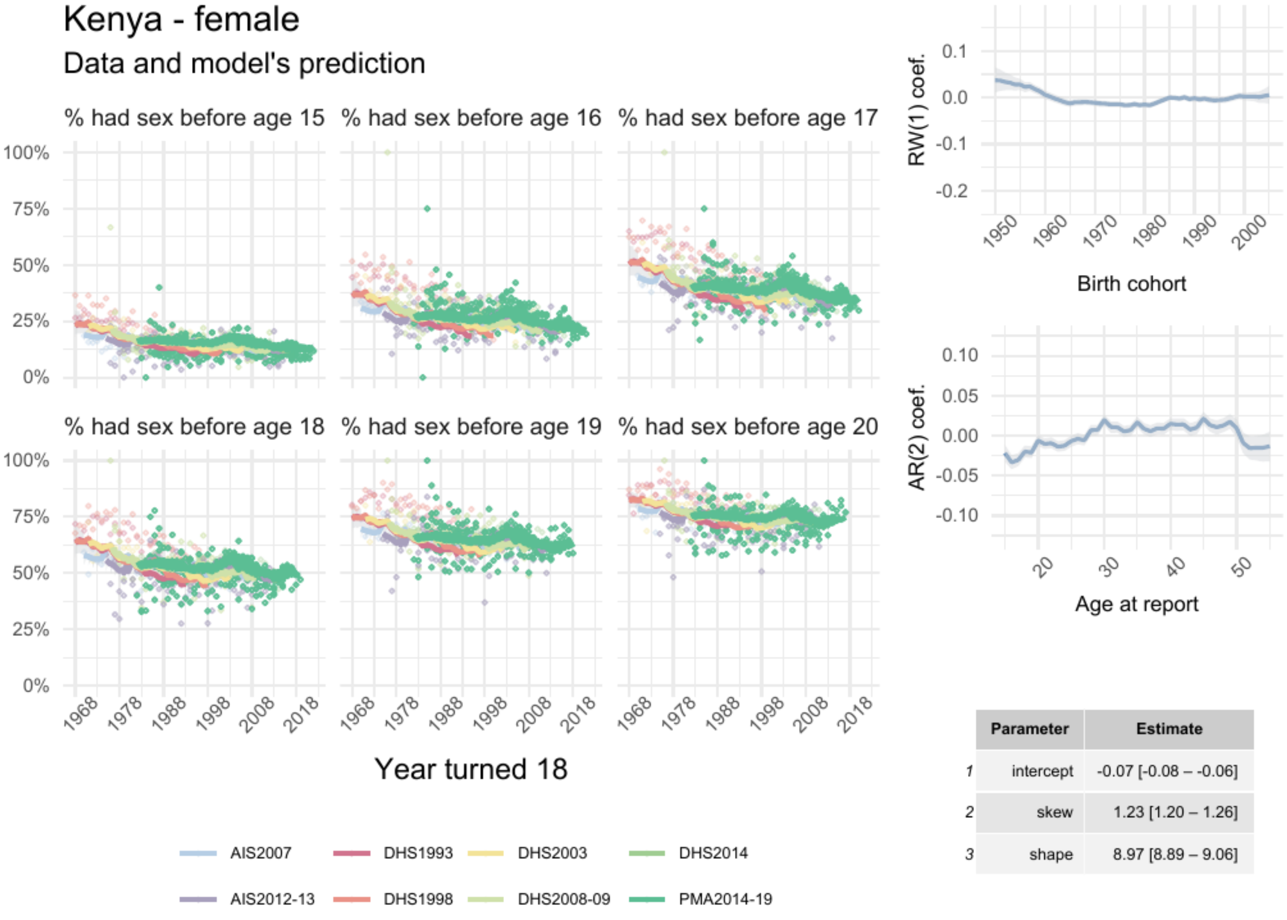

	Parameter	Estimate
1	intercept	-0.07 [-0.08 – -0.06]
2	skew	1.23 [1.20 – 1.26]
3	shape	8.97 [8.89 – 9.06]

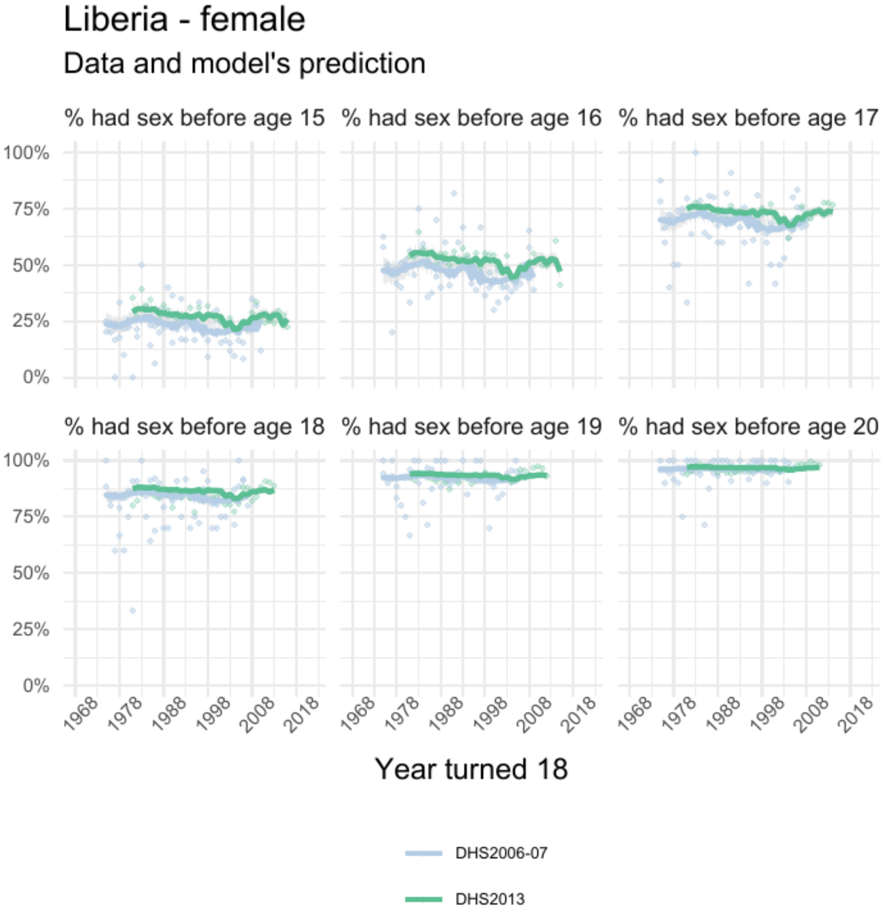

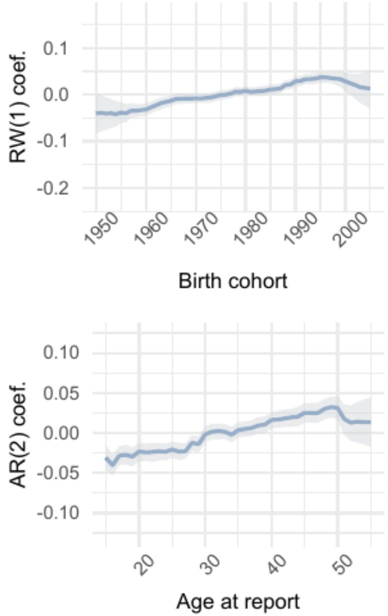

	Parameter	Estimate
1	intercept	0.03 [0.02 – 0.04]
2	skew	1.42 [1.33 – 1.52]
3	shape	14.48 [14.17 – 14.81]

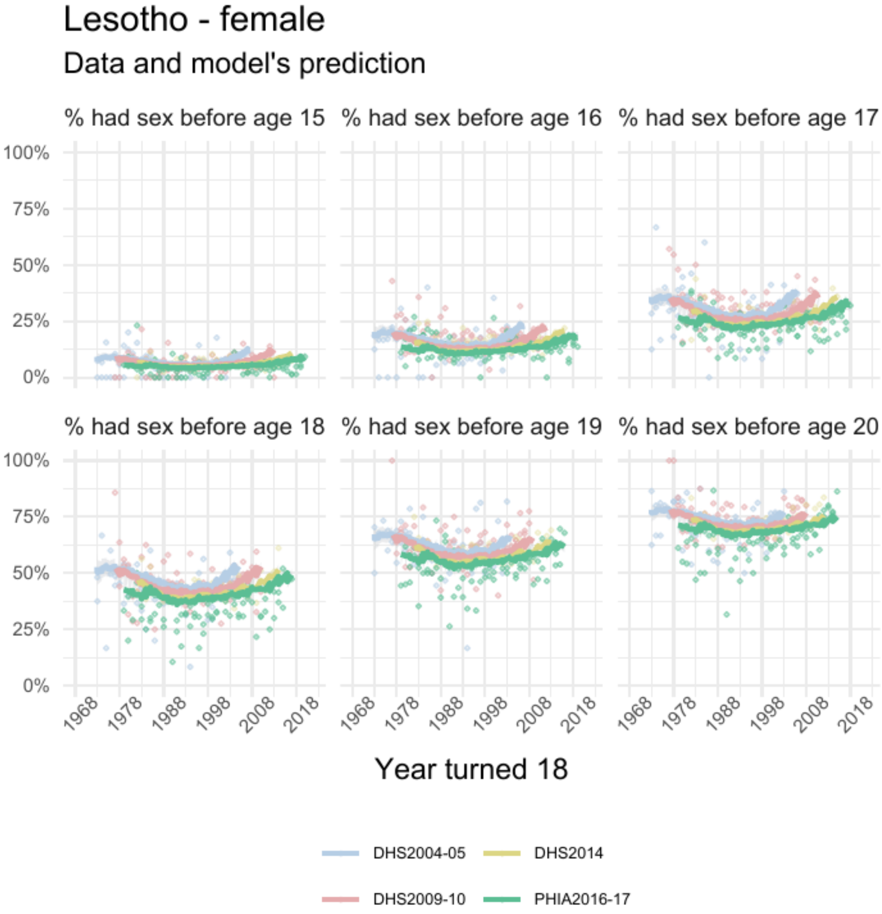

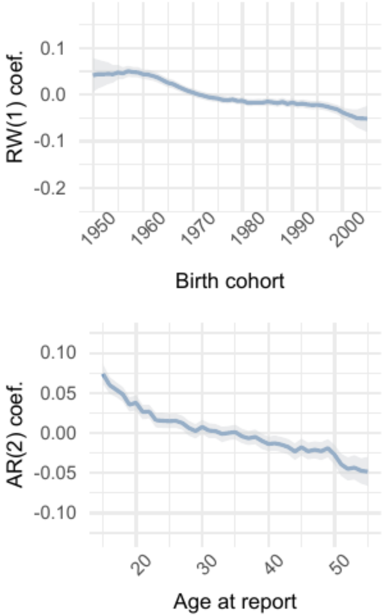

	Parameter	Estimate
1	intercept	-0.05 [-0.06 – -0.04]
2	skew	1.89 [1.78 – 2.00]
3	shape	10.12 [9.95 – 10.31]

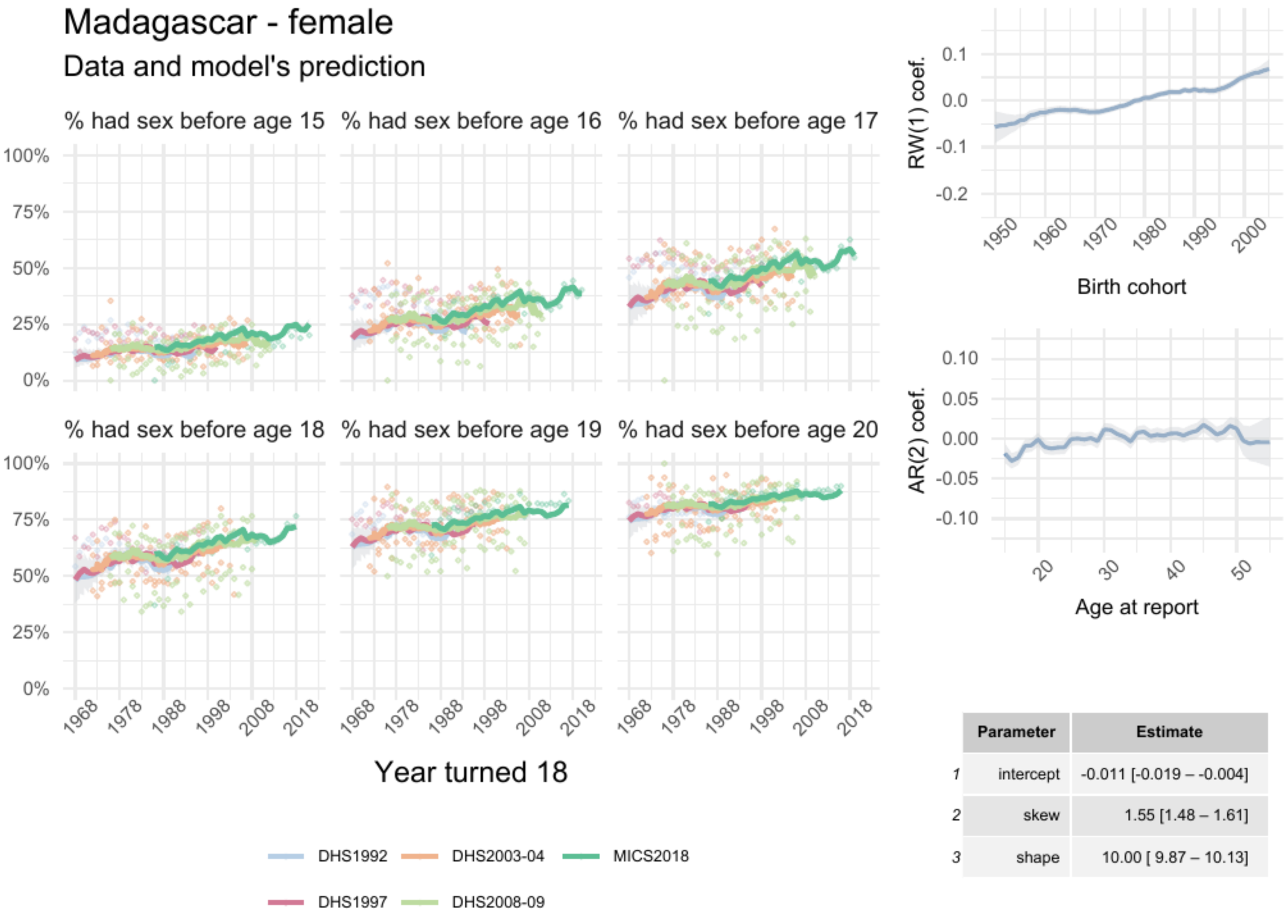

	Parameter	Estimate
1	intercept	-0.011 [-0.019 – -0.004]
2	skew	1.55 [1.48 – 1.61]
3	shape	10.00 [9.87 – 10.13]

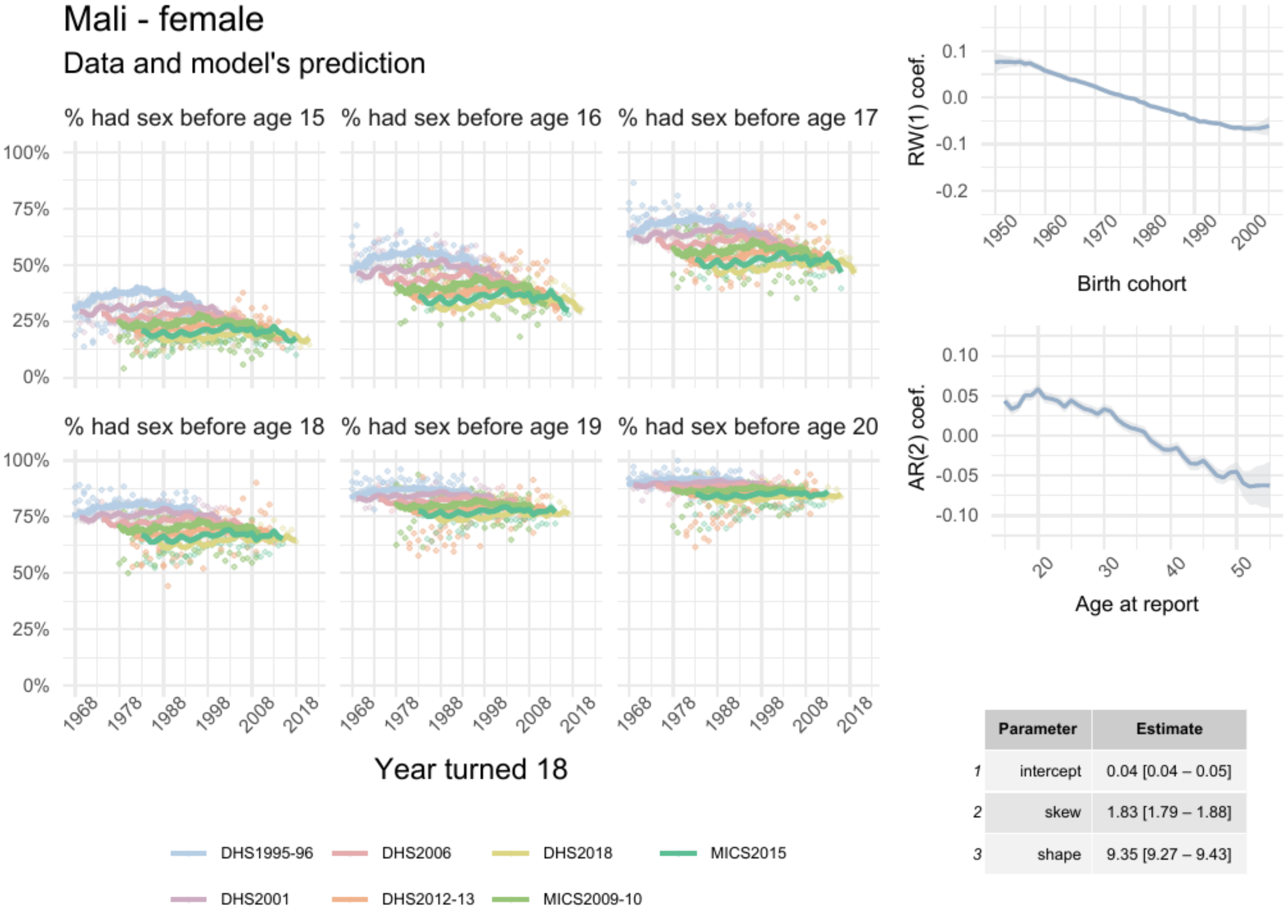

	Parameter	Estimate
1	intercept	0.04 [0.04 – 0.05]
2	skew	1.83 [1.79 – 1.88]
3	shape	9.35 [9.27 – 9.43]

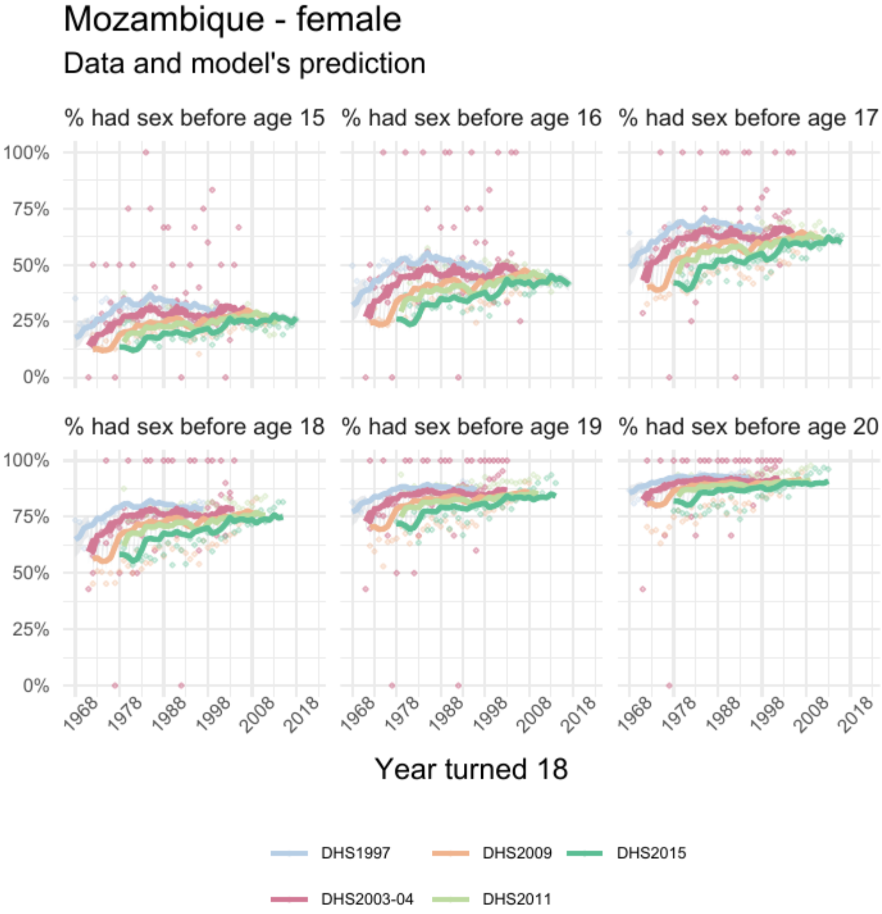

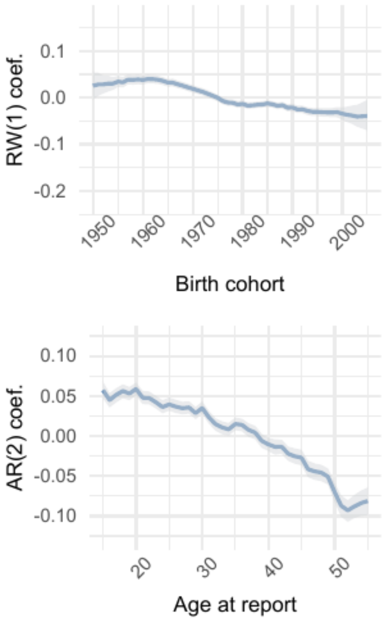

	Parameter	Estimate
1	intercept	-0.009 [-0.017 – -0.001]
2	skew	1.29 [1.24 – 1.35]
3	shape	10.77 [10.61 – 10.94]

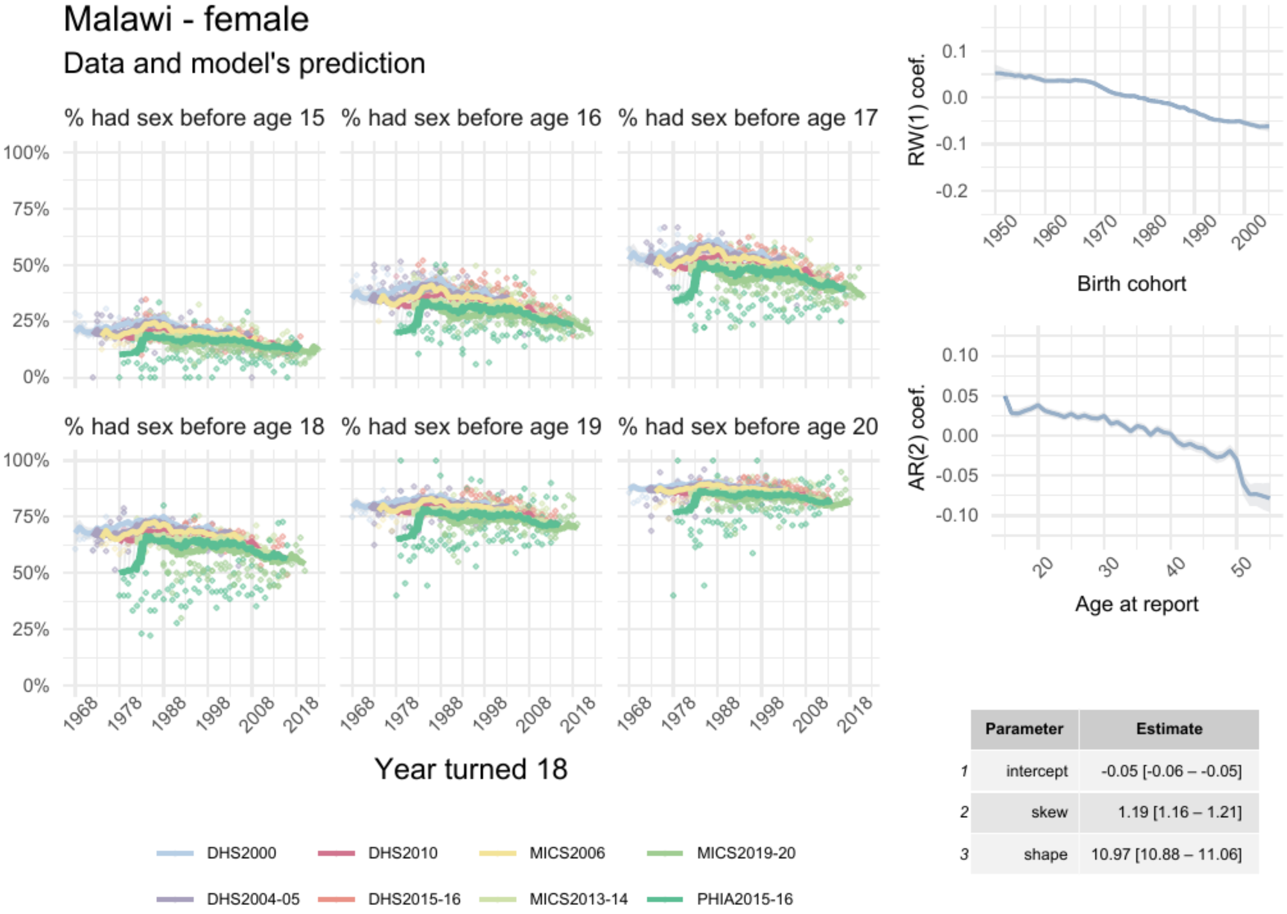

	Parameter	Estimate
1	intercept	-0.05 [-0.06 – -0.05]
2	skew	1.19 [1.16 – 1.21]
3	shape	10.97 [10.88 – 11.06]

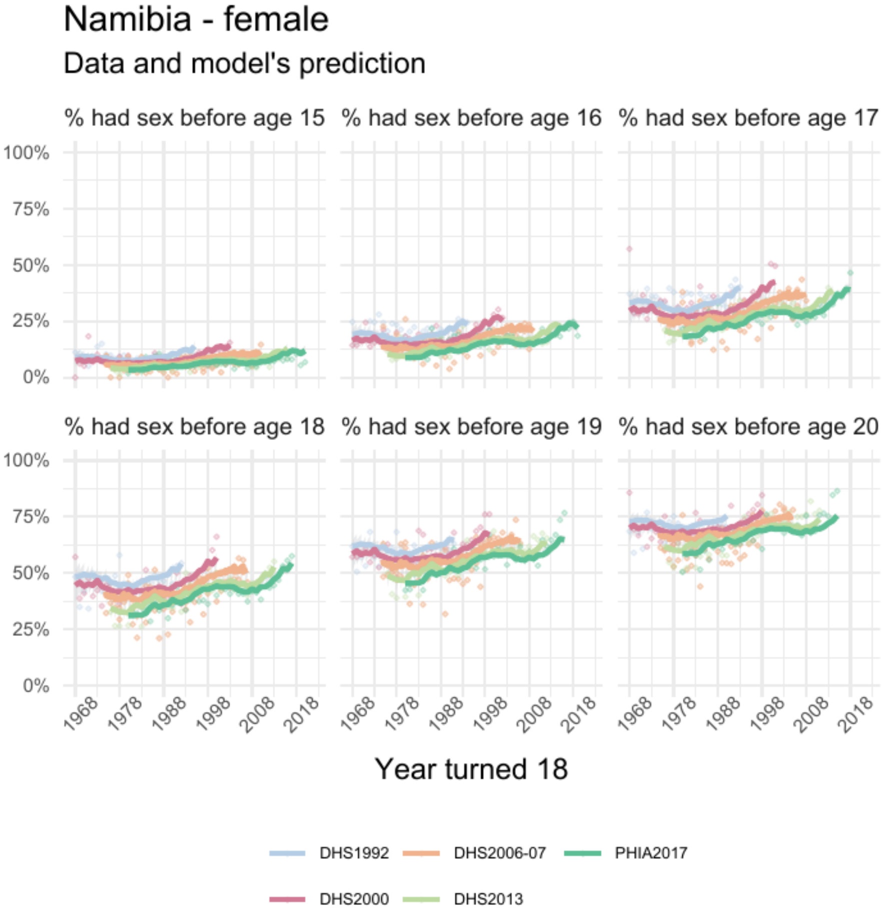

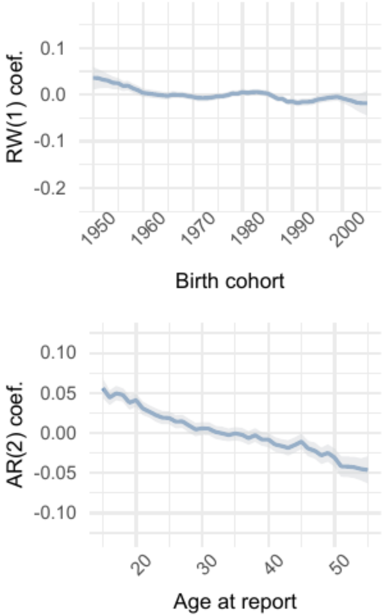

	Parameter	Estimate
1	intercept	-0.016 [-0.027 – -0.006]
2	skew	2.38 [2.24 – 2.53]
3	shape	8.76 [8.61 – 8.89]

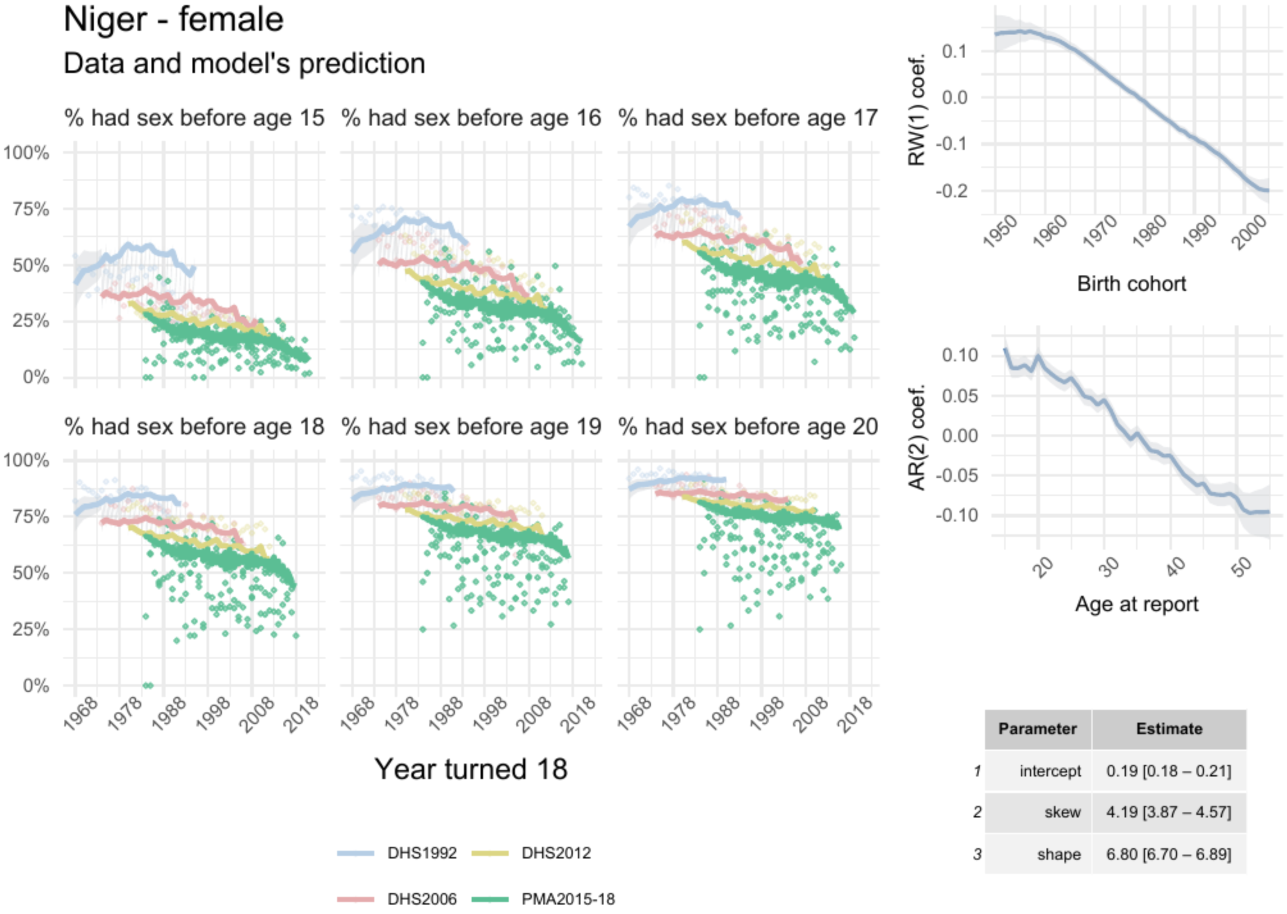

	Parameter	Estimate
1	intercept	0.19 [0.18 – 0.21]
2	skew	4.19 [3.87 – 4.57]
3	shape	6.80 [6.70 – 6.89]

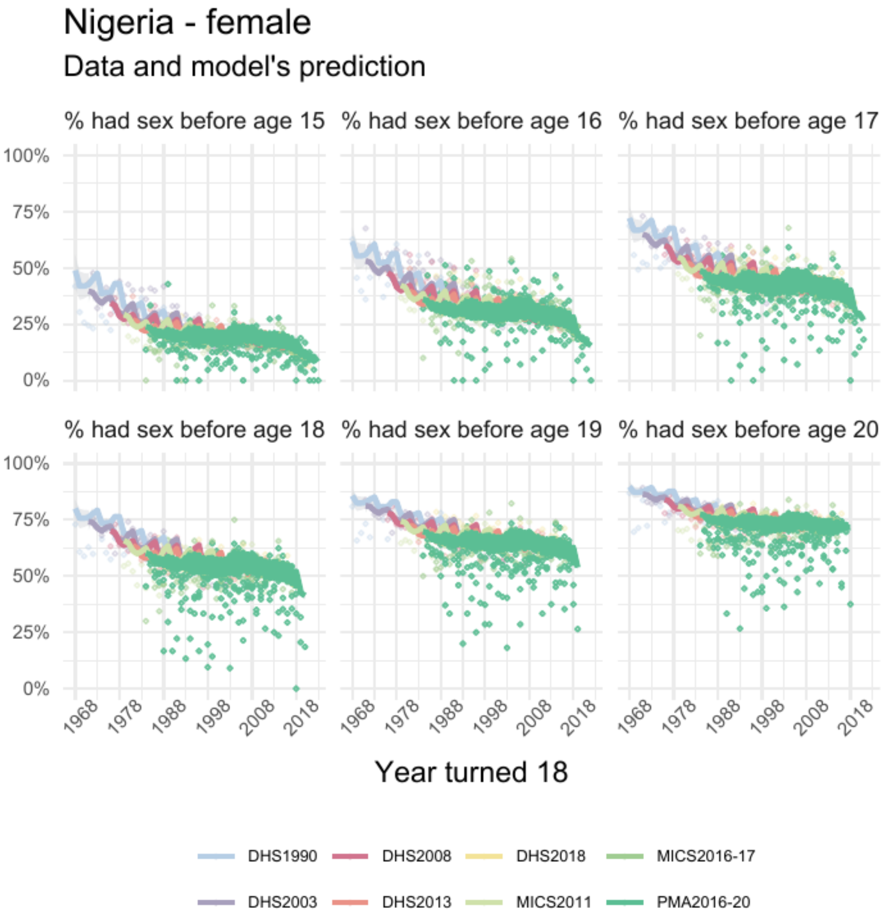

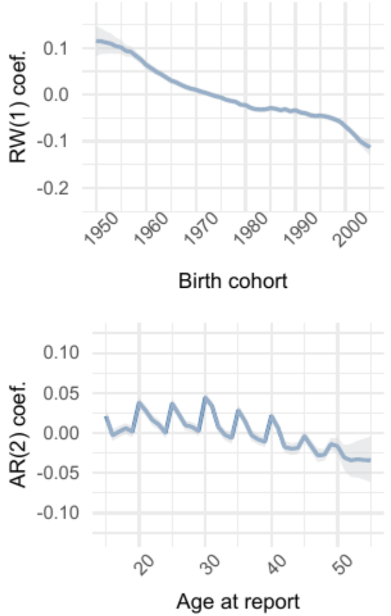

	Parameter	Estimate
1	intercept	0.04 [0.03 – 0.04]
2	skew	1.87 [1.83 – 1.90]
3	shape	7.16 [7.11 – 7.20]

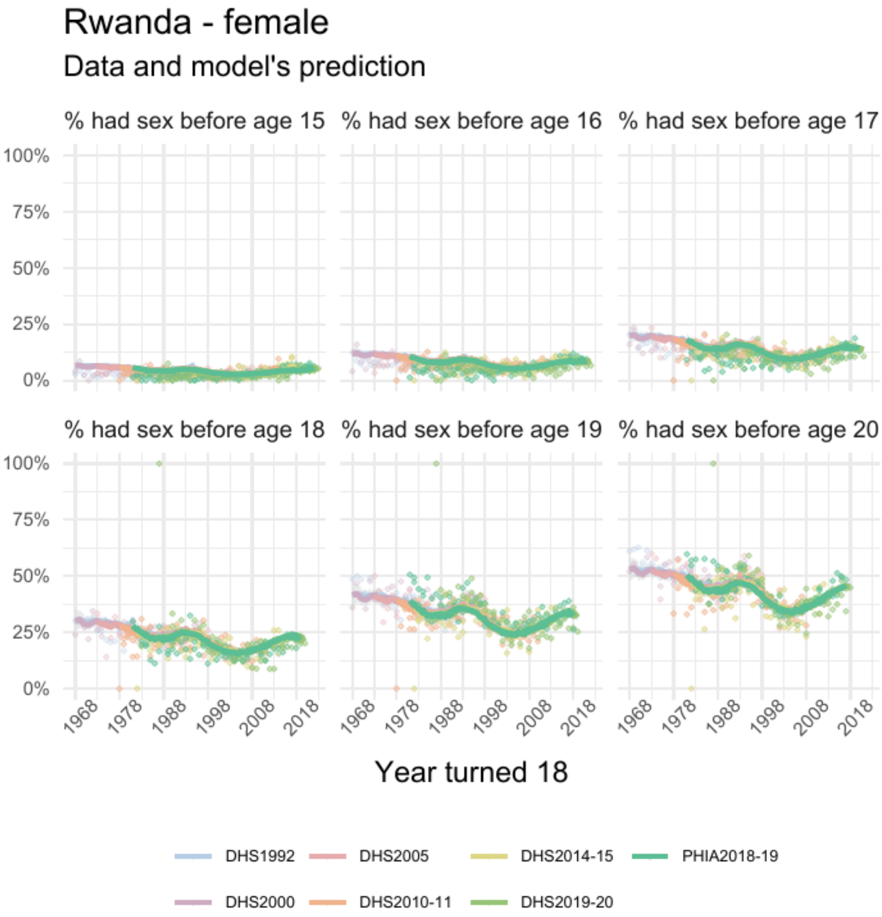

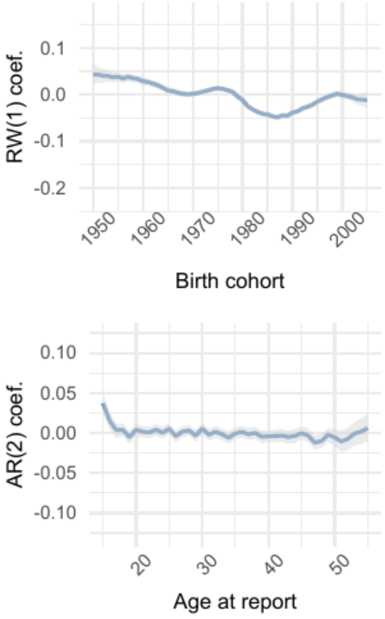

	Parameter	Estimate
1	intercept	-0.19 [-0.19 – -0.18]
2	skew	1.41 [1.37 – 1.45]
3	shape	8.15 [8.06 – 8.25]

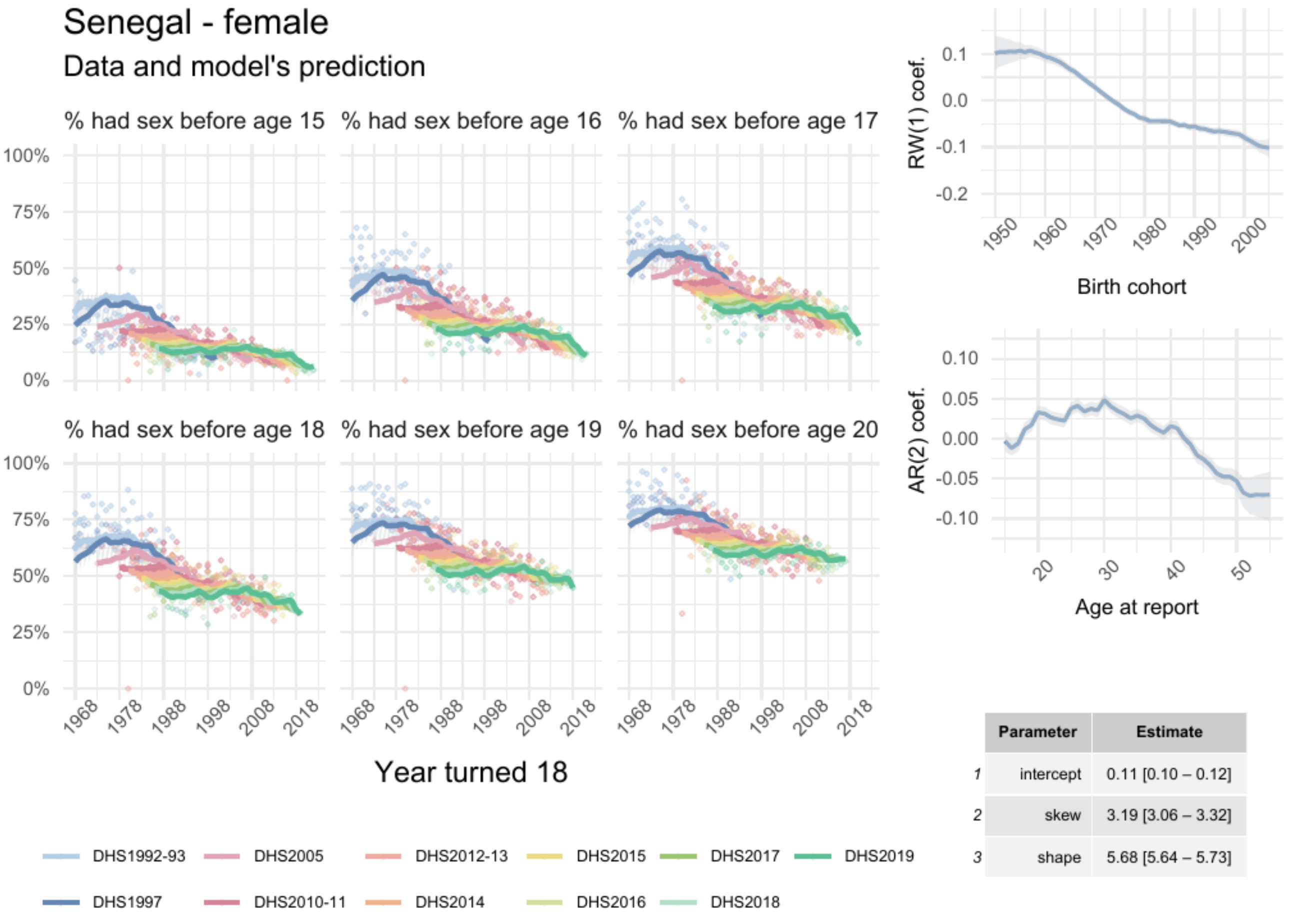

	Parameter	Estimate
1	intercept	0.11 [0.10 – 0.12]
2	skew	3.19 [3.06 – 3.32]
3	shape	5.68 [5.64 – 5.73]

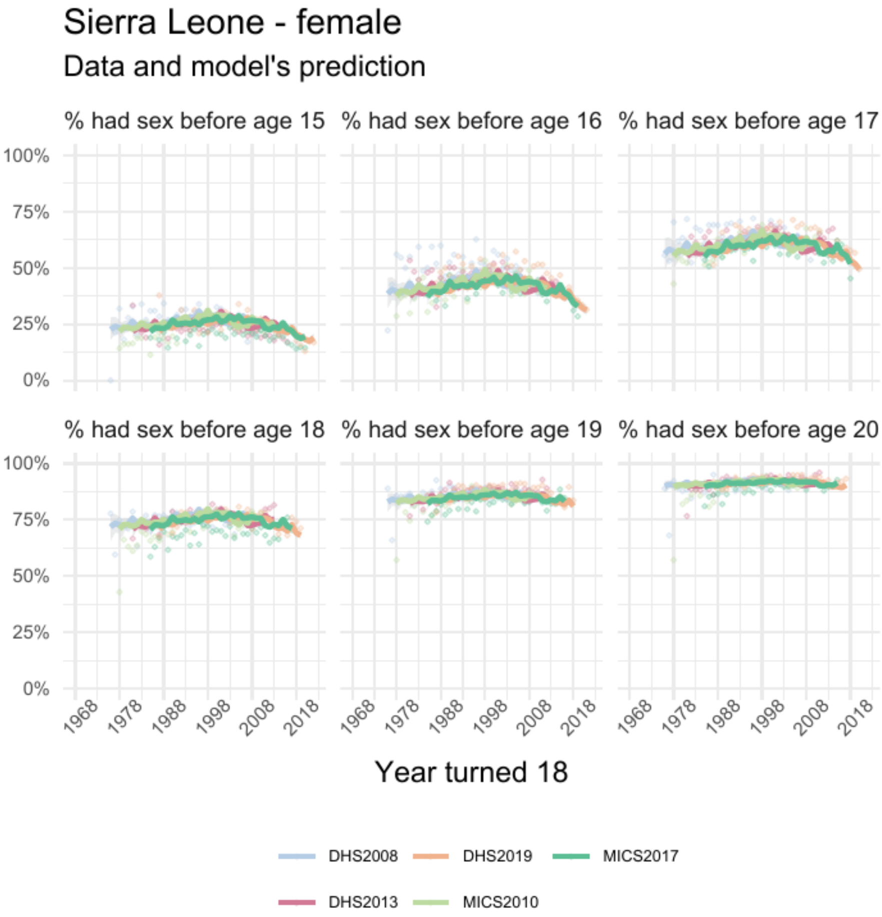

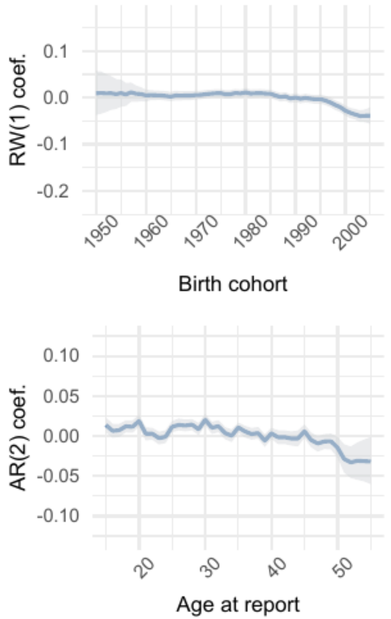

	Parameter	Estimate
1	intercept	-0.04 [-0.04 – -0.03]
2	skew	0.96 [0.93 – 0.98]
3	shape	12.06 [11.93 – 12.19]

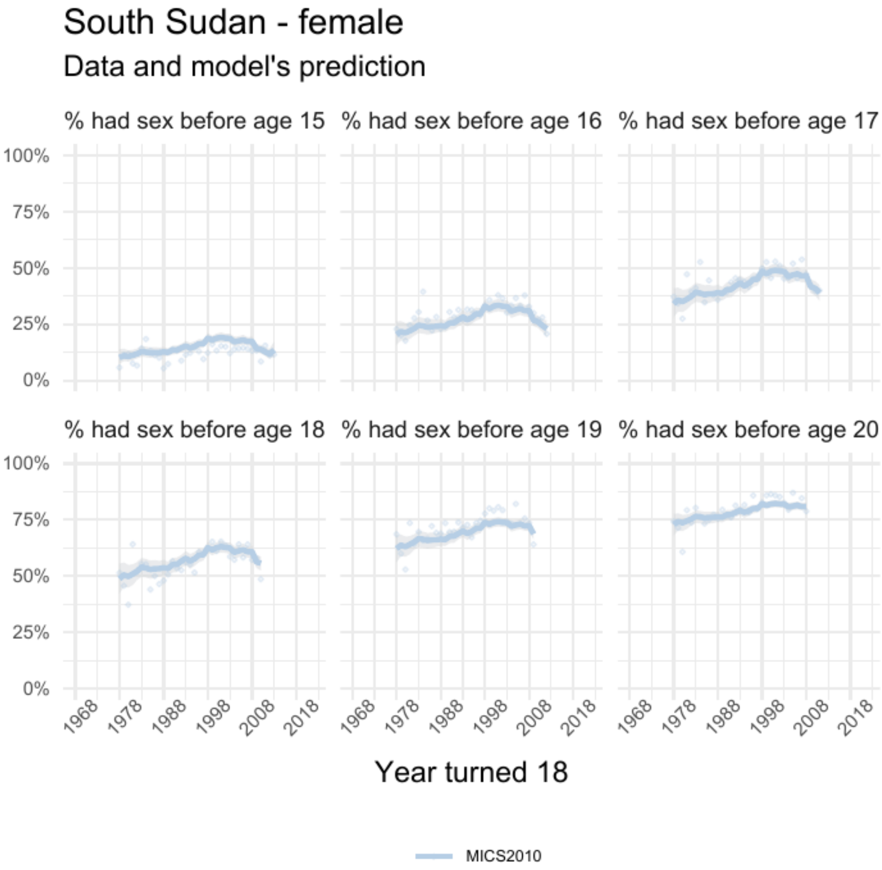

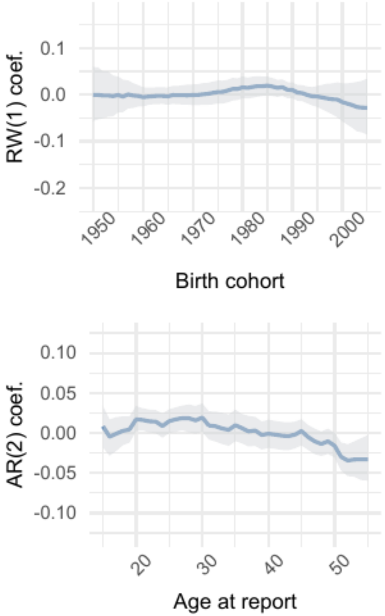

	Parameter	Estimate
1	intercept	0.002 [-0.020 – 0.023]
2	skew	1.99 [1.79 – 2.20]
3	shape	8.80 [8.53 – 9.06]

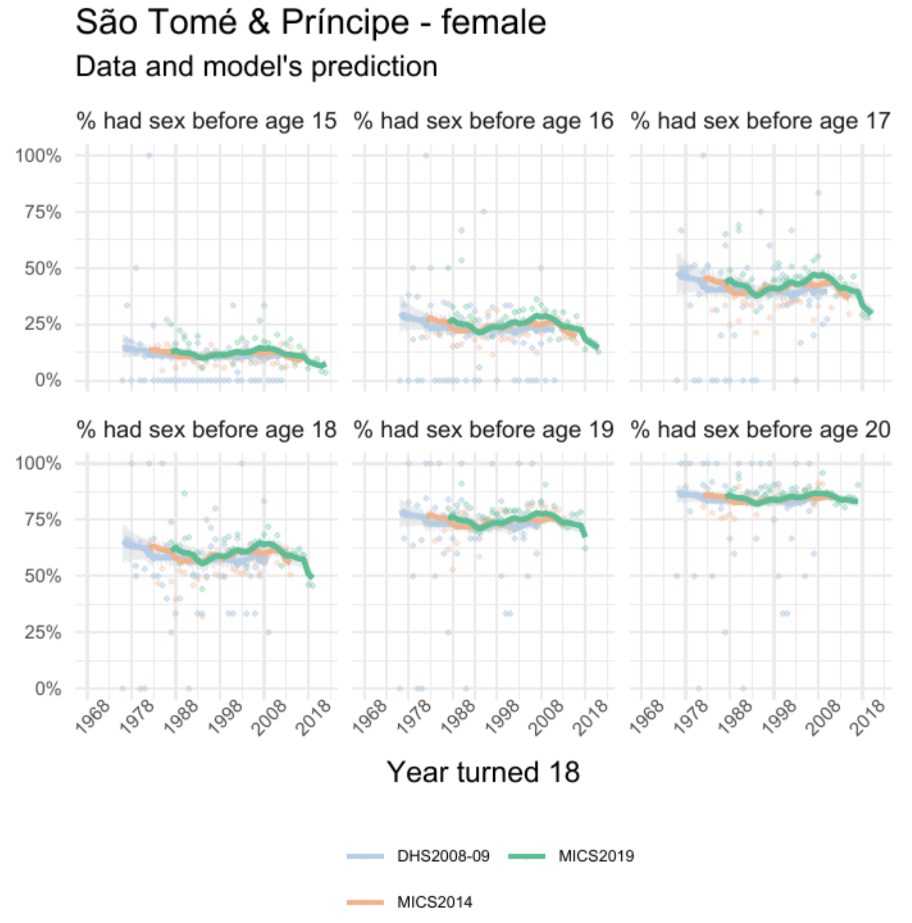

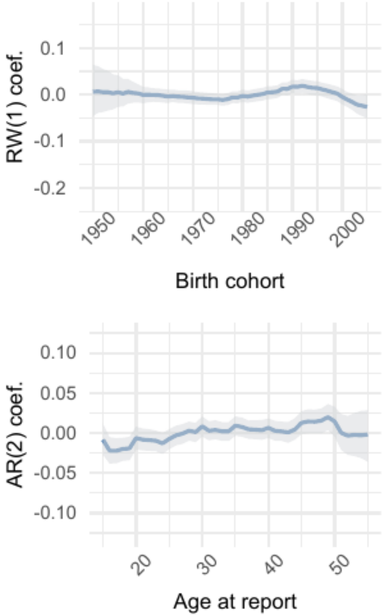

	Parameter	Estimate
1	intercept	-0.06 [-0.07 – -0.05]
2	skew	1.27 [1.17 – 1.38]
3	shape	11.88 [11.47 – 12.30]

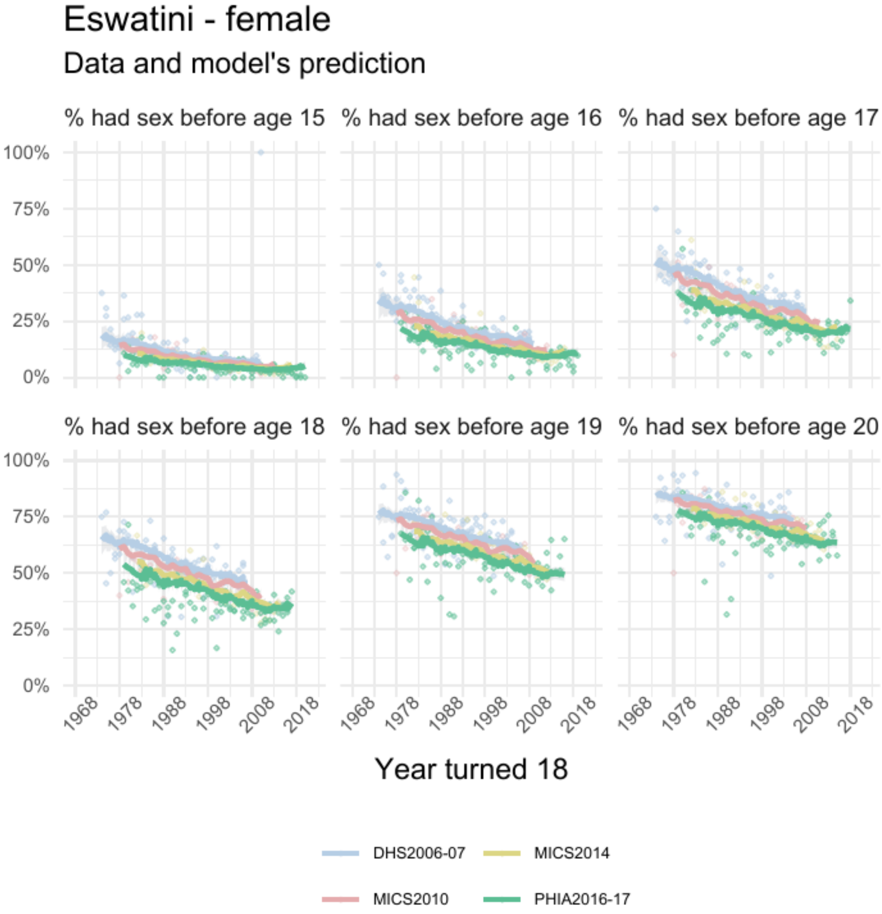

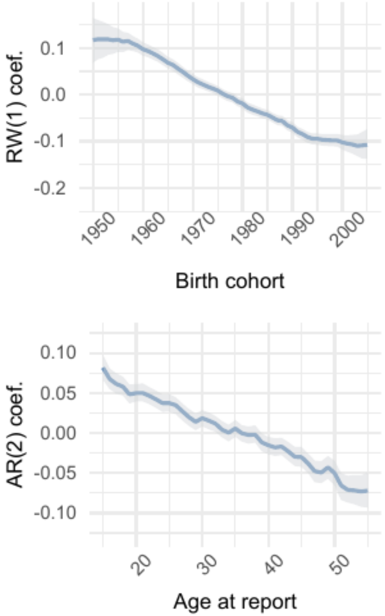

	Parameter	Estimate
1	intercept	-1e-02 [-3e-02 – 7e-04]
2	skew	2.27 [2.10 – 2.44]
3	shape	9.45 [9.26 – 9.64]

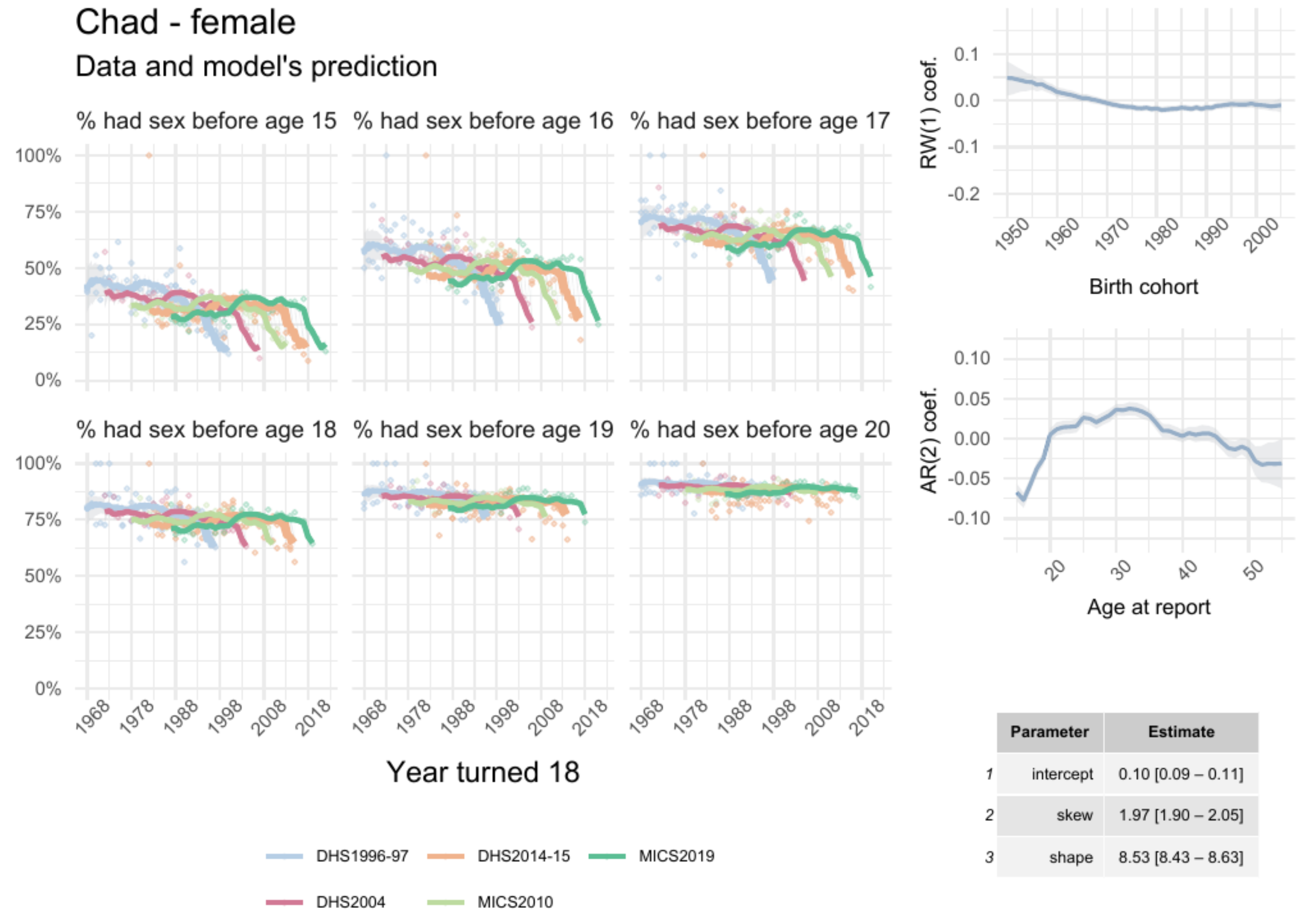

	Parameter	Estimate
1	intercept	0.10 [0.09 – 0.11]
2	skew	1.97 [1.90 – 2.05]
3	shape	8.53 [8.43 – 8.63]

Togo - female

Data and model's prediction

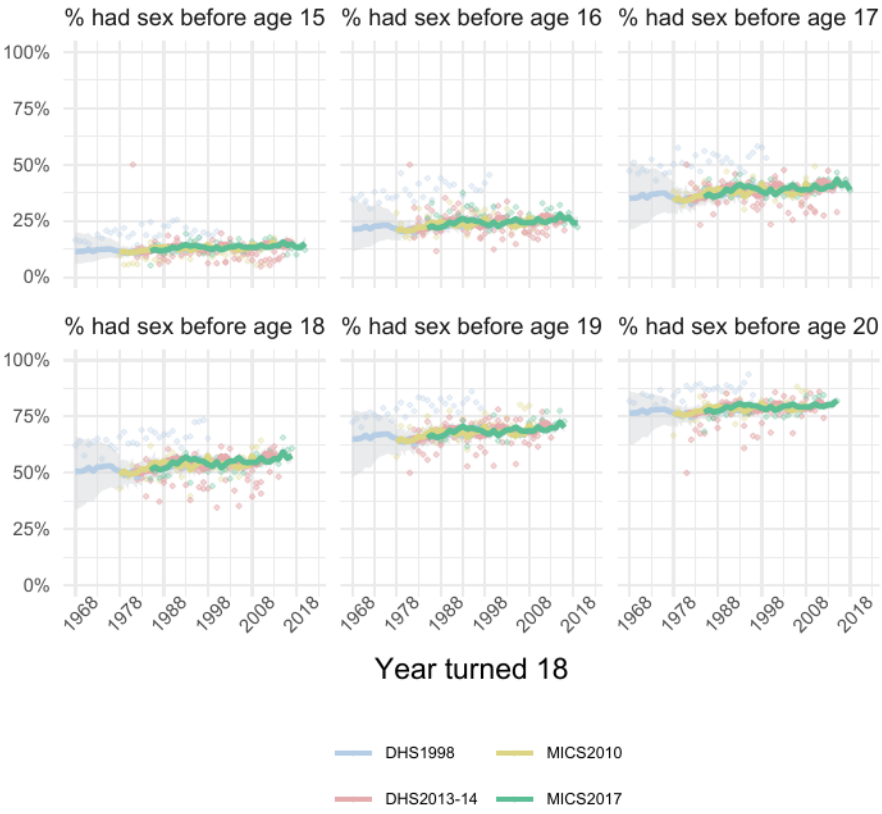

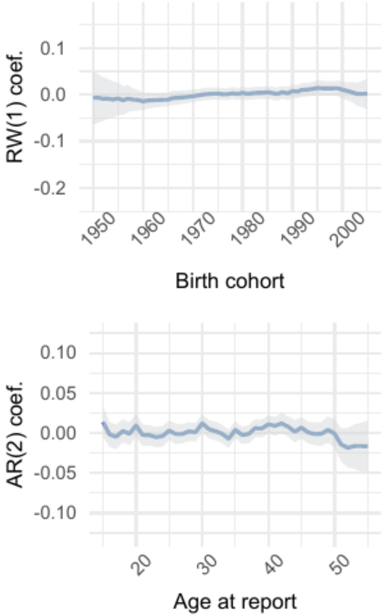

	Parameter	Estimate
1	intercept	-0.09 [-0.10 – -0.08]
2	skew	1.11 [1.06 – 1.17]
3	shape	10.78 [10.57 – 10.99]

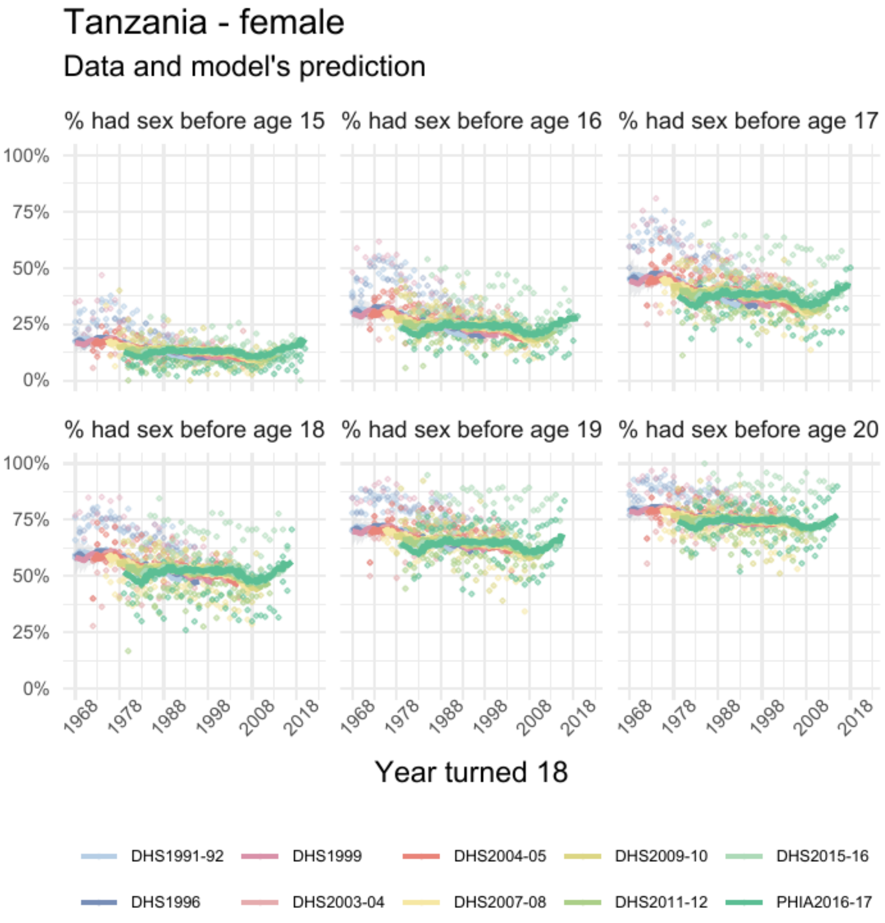

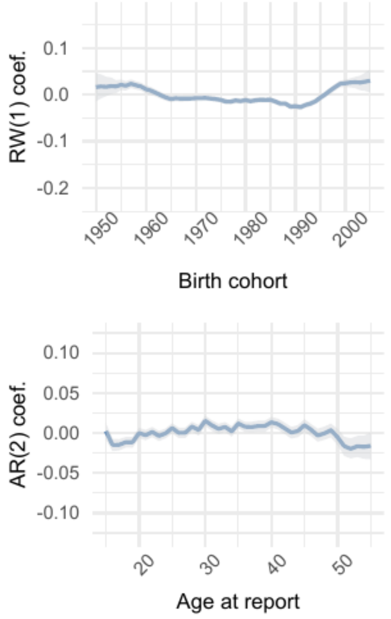

	Parameter	Estimate
1	intercept	-0.006 [-0.013 – 0.001]
2	skew	1.87 [1.81 – 1.92]
3	shape	8.57 [8.50 – 8.65]

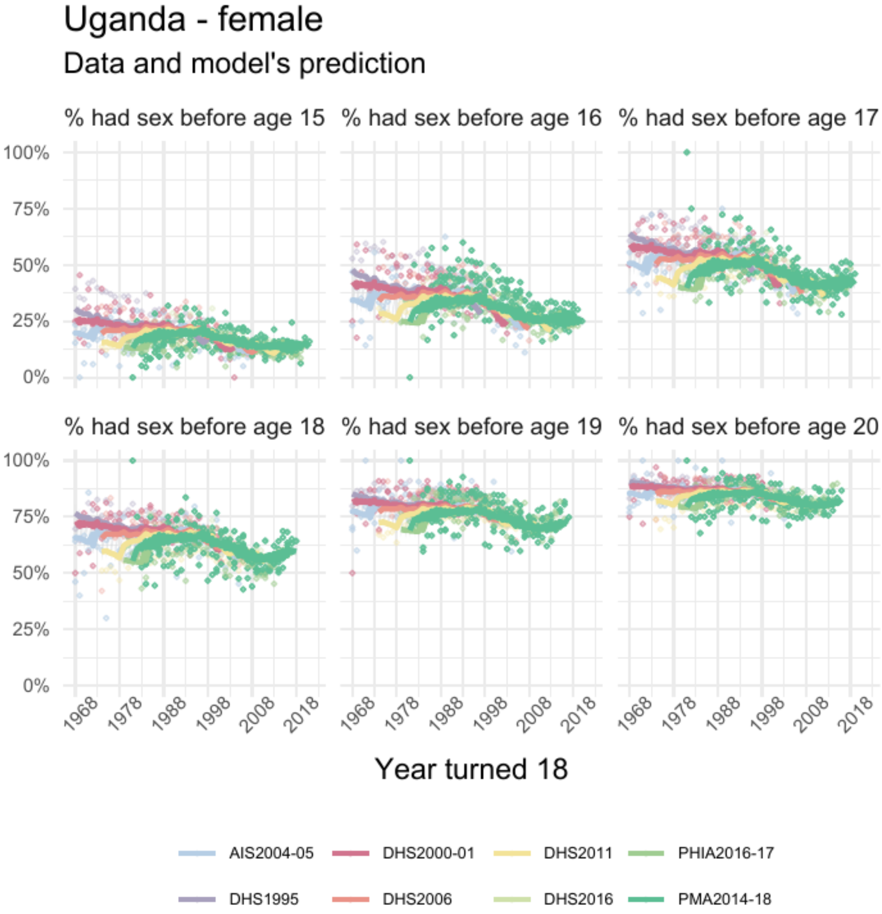

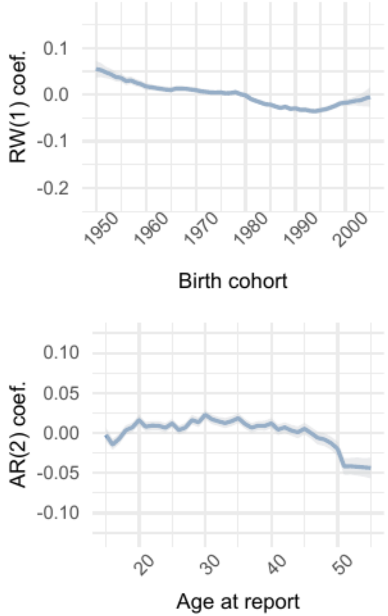

	Parameter	Estimate
1	intercept	-0.03 [-0.04 – -0.02]
2	skew	1.32 [1.29 – 1.35]
3	shape	10.25 [10.15 – 10.34]

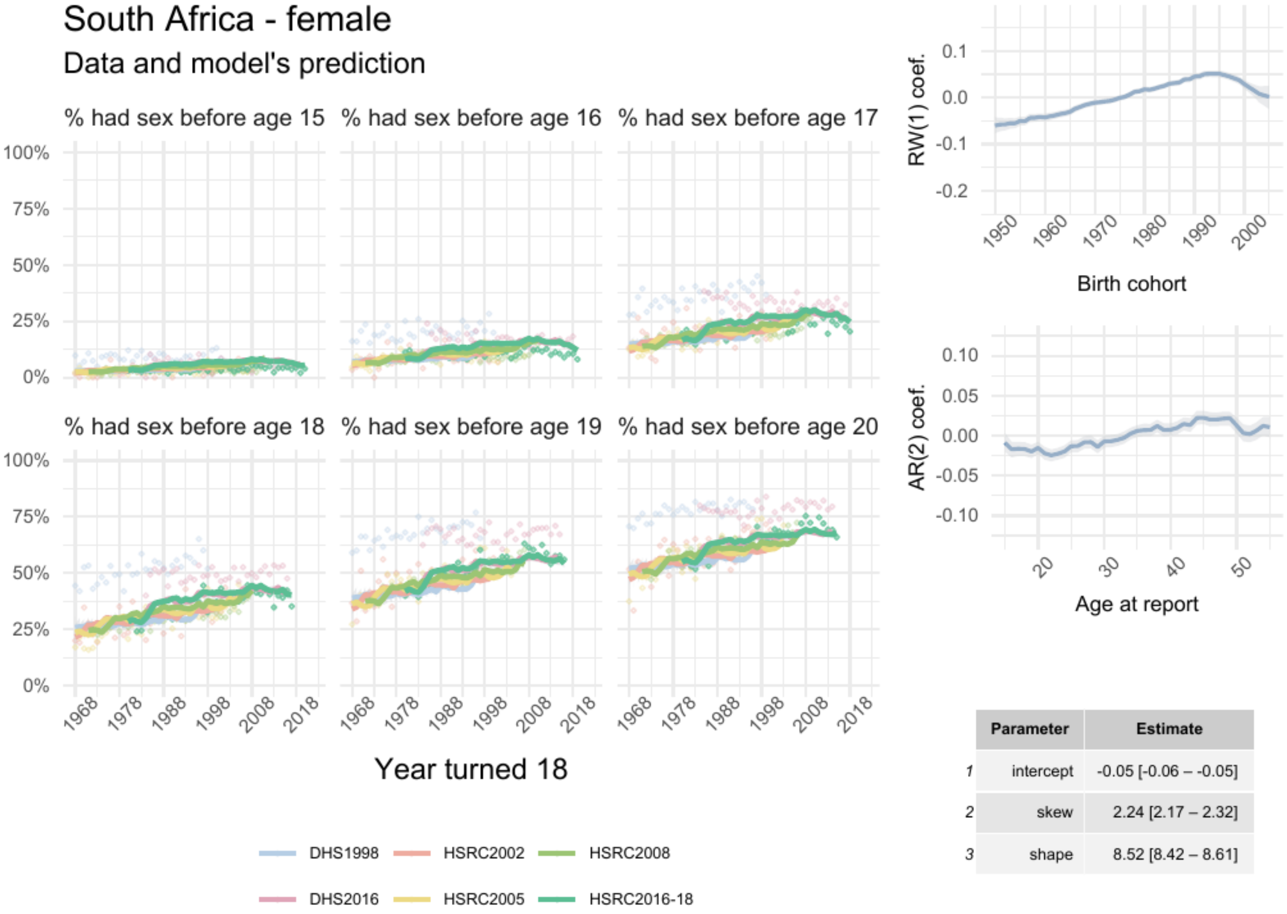

	Parameter	Estimate
1	intercept	-0.05 [-0.06 – -0.05]
2	skew	2.24 [2.17 – 2.32]
3	shape	8.52 [8.42 – 8.61]

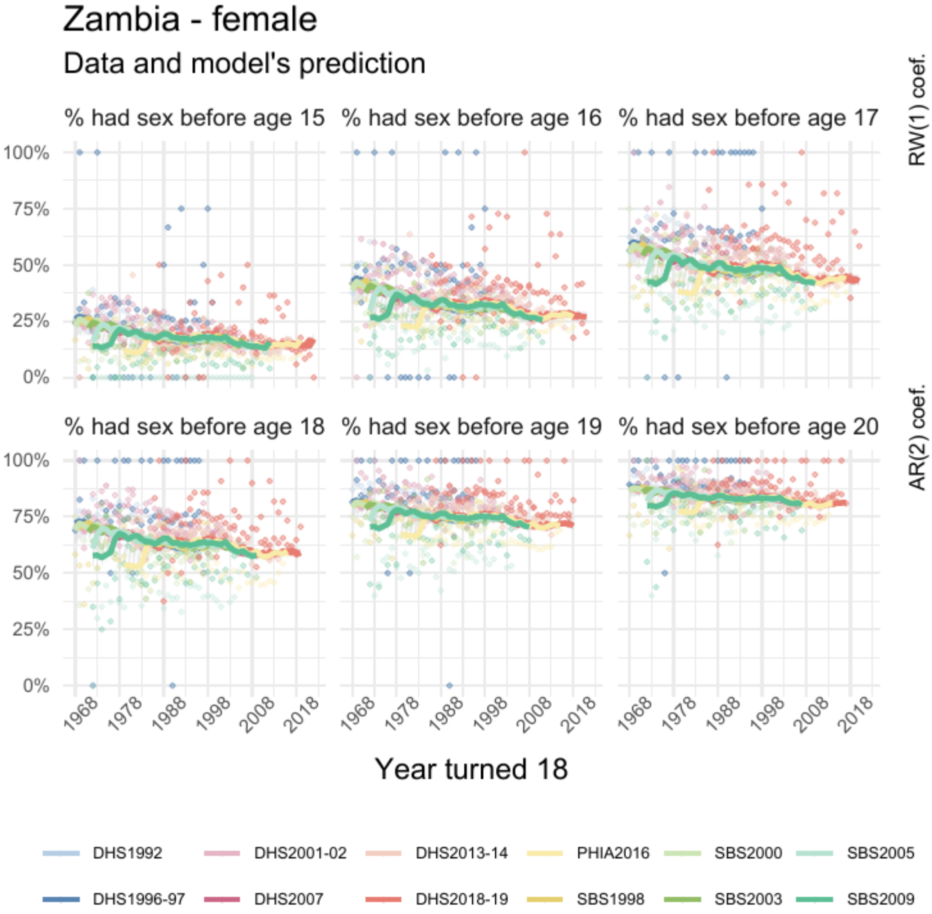

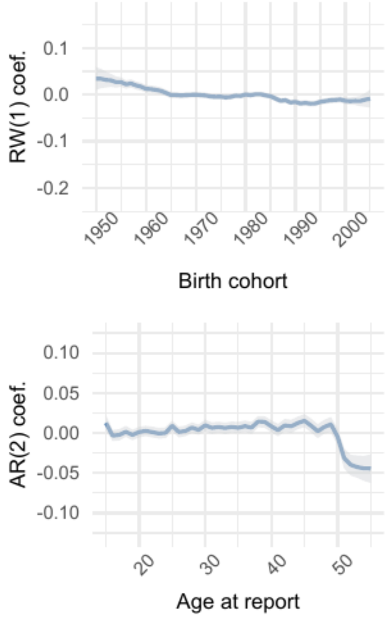

	Parameter	Estimate
1	intercept	0.02 [0.01 – 0.02]
2	skew	1.86 [1.79 – 1.93]
3	shape	9.38 [9.27 – 9.49]

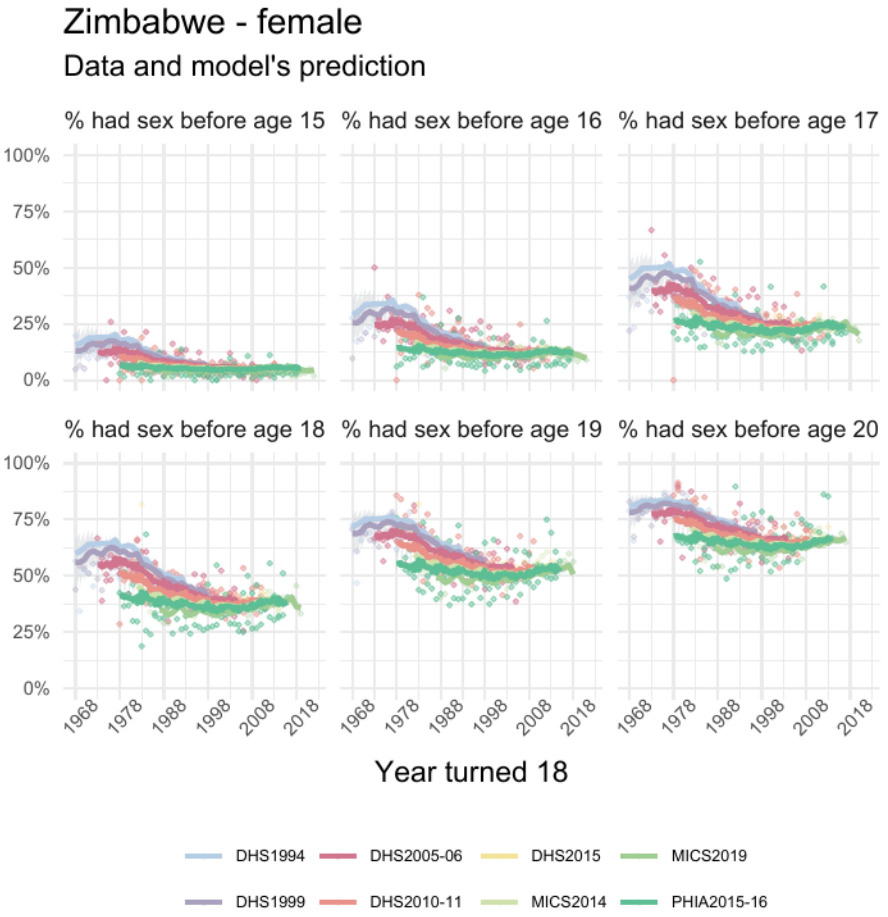

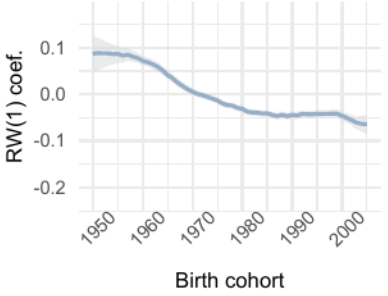

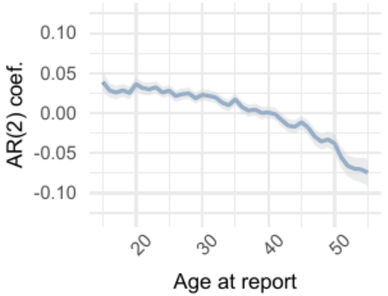

	Parameter	Estimate
1	intercept	-0.04 [-0.05 – -0.03]
2	skew	2.10 [2.01 – 2.19]
3	shape	8.99 [8.88 – 9.10]
